# A comparison of single-domain and multidomain executive functions cognitive training for enhancing cognition and well-being in older adults

**DOI:** 10.1007/s00426-026-02249-x

**Published:** 2026-03-07

**Authors:** Lan Nguyen, Karen Murphy, Glenda Andrews, Amanda Duffy

**Affiliations:** School of Applied Psychology, Griffith University, Gold Coast, QLD 4222 Australia

## Abstract

This study investigated the efficacy of single-domain (working memory; WM) and multidomain core executive functions (EFs; cognitive flexibility, inhibitory control, WM) cognitive training programs in enhancing cognitive performance (measured objectively and subjectively) and well-being. Participants comprised 66 healthy older adults (*M*_age_ = 69.58, *SD* = 7.04) who were assigned to a 4-week single-domain WM, multidomain EF, or active control program. Bayesian analyses yielded ambiguous evidence regarding near-transfer to global executive functioning for both training groups. However, findings revealed substantial evidence for training-related far-transfer (prospective memory) for the multidomain EF group and mixed evidence for the single-domain WM group. No far-transfer was observed for fluid intelligence in either training group. Post-test performance for the multidomain EF group was comparable to that of an untrained young adult sample (*M*_age_ = 22.42, *SD* = 5.14) on some trained and near-transfer executive functioning measures (*n*-back, mixing costs, global EF accuracy), though evidence for comparable performance on other outcomes was ambiguous. The single-domain WM group was comparable to young adults on 1-back and 2-back levels of the trained WM updating measure (*n*-back). Neither group demonstrated comparable performance to young adults on the far-transfer task (Virtual Week). Both multidomain and single-domain programs, but not the control, elevated participants’ subjective perceptions of their own cognitive abilities (particularly attention and memory); however, improvements in well-being were observed across all groups. These findings provide initial support for multidomain training (targeting core EFs) over single-domain cognitive training in promoting transfer to objective and subjective measures of cognition. However, considering the ambiguous evidence across numerous outcomes, further research applying Bayesian analyses is warranted to evaluate the strength of evidence regarding cognitive training-related changes and provide clearer insight into the potential that cognitive training might offer.

**Supplementary Information:**

The online version contains supplementary material available at 10.1007/s00426-026-02249-x.

## Introduction

Population aging reflects the achievements of society’s medical, technological, and public health advancements, with the United Nations estimating that one in six people in the global population will be 65 years or older by 2050 (United Nations, 2022). Aging, however, is associated with a higher likelihood of physical and cognitive decline (World Health Organization, 2022), which may adversely impact quality of life and psychological well-being (Steptoe et al., 2015). The simultaneous increase in the proportion of older adults in the population and declines in physical, cognitive, and mental health have profound implications for healthcare, social services, policy-making, and the global economy, which subsequently impacts health and well-being for the entire population (Mason et al., 2022; Wang et al., 2022). Thus, efforts to slow or reverse the progression of age-related health declines to foster healthy aging have been a prominent focus in aging research, as even small improvements can yield large-scale societal benefits (Au et al., 2015; Dougherty et al., 2016).

A prominent intervention that has been investigated in relation to improvements in cognition and well-being in the older adult population is cognitive training, which involves the structured practice of cognitively challenging tasks (Klimova, 2016; Lampit et al., 2014). In theory, repeated practice on a cognitive task should improve the efficiency of neurocognitive systems in performing the basic skills or processes exercised; these improvements consequently facilitate the efficiency of carrying out other tasks or everyday activities that also utilize these skills or processes (Gathercole et al., 2019; Taatgen, 2021). This has implications for individuals’ quality of life as enhanced cognitive functioning is associated with better psychological well-being (Bures et al., 2016; Zhang et al., 2022). However, the concept of *cognitive transfer* has been a source of contention within the cognitive training literature (Harvey et al., 2018), with debate regarding the extent of possible skills transfer and what is classified as near (or narrow) transfer versus far (or broad) transfer (see Noack et al., 2009 and Schmiedek, 2021 for a discussion). The most common definition of near-transfer represents the generalization of training-related gains to outcomes closely related to the trained task (e.g., tasks tapping the same or similar cognitive construct), whereas far-transfer involves generalizations to more distantly related outcomes (e.g., tasks tapping a different cognitive construct, and subjective outcomes and well-being) (Gobet & Sala, 2023; Green et al., 2019). Whilst near-transfer has often been demonstrated, clear evidence of far-transfer has been more elusive (Gobet & Sala, 2023; Sala & Gobet, 2019; Turnbull et al., 2022). As the goal of cognitive training for older adults is to achieve gains beyond the training context (i.e., enable more efficient execution of everyday functional activities and improved well-being), further research is needed to understand whether, how, and under what conditions training-related transfer may occur. This study seeks to address this issue.

One key element that substantially impacts the potential for transfer is the cognitive domain(s) trained (Walton et al., 2019), with working memory (WM) being a popular candidate. WM is a limited-capacity executive function allowing for the simultaneous maintenance and processing of information (Diamond, 2013). In particular, WM updating reflects the ability actively monitor and replace no-longer-relevant information with task-relevant content (Nyberg & Eriksson, 2016). This WM system is integral for carrying out other cognitive tasks and represents a key predictor of fluid intelligence, making it an ideal function to target for cognitive enhancement (Könen et al., 2021). Despite its significance, numerous meta-analyses have reported limited or no far-transfer effects as a function of training targeting WM alone (Dougherty et al., 2016; Melby-Lervåg et al., 2016; Sala et al., 2019).

WM is only one of a “core” set of executive functions (EFs). Miyake et al.’s (2000) influential framework identified three core EFs: WM updating, cognitive flexibility (the ability to switch between tasks, mental sets, or perspectives), and inhibitory control (the ability to suppress dominant responses or irrelevant information). Diamond (2013) similarly described WM, cognitive flexibility, and inhibitory control as core EFs, but conceptualized WM more broadly (encompassing both maintenance and manipulation), rather than focusing specifically on updating, as Miyake et al. do. Together, these EFs form the foundations of higher-order cognitive processing and everyday functioning. These EFs are also interconnected and often co-activated when executing complex tasks (Diamond, 2013). Thus, training all three core EFs has the potential to produce greater cognitive transfer and well-being than training WM alone.

Training multiple cognitive domains (henceforth referred to as *multidomain training*^1^) can, in theory, increase the generalization of training by exercising a broader range of cognitive processes and activating a larger network of neural circuitry, thereby improving numerous skills and processes (Cochrane & Green, 2021; Guye et al., 2021). This is supported by the findings of meta-analyses reporting transfer to more cognitive domains in studies that employed multidomain training programs compared with studies that employed single-domain training (Lampit et al., 2014; Nguyen et al., 2019). Whilst this provides an indication of the potential for transfer in multidomain training, direct comparisons between multidomain and single-domain training within individual studies are few. The potential of multidomain training may be further bolstered by targeting multiple core EFs given their strong connections to critical brain regions associated with aging and well-being, including the prefrontal cortex and anterior cingulate cortex (e.g., Goldstein & Naglieri, 2014; King, 2019). Despite the theoretically-based recommendations for multidomain training, very few studies have directly compared the efficacy of multidomain training to single-domain training within an older adult population, and no studies, to the authors’ knowledge, have compared multidomain EF training to single-domain WM training. Comparing multidomain and single-domain training is important for the understanding of the optimal conditions that may promote transfer and will therefore be a focus of this study.

Of the few studies that have compared multidomain and single-domain cognitive training, the results appear to favor multidomain training, reporting improved performance on many far-transfer tasks. For instance, Karbach and Kray (2009) reported stronger improvements in task-switching performance and transfer to other EF tasks as well as fluid intelligence within task-switching training compared to single-task training. Using a bespoke game program, *Hotel Plastisse*, Binder et al. (2016) compared a multidomain condition (targeting inhibition, visuomotor functions, and spatial navigation), to three separate single-domain conditions, each targeting one of the cognitive domains included in the multidomain program. After training, the multidomain group demonstrated an increase in performance for visuomotor function and spatial navigation tasks (comparable to corresponding single-domain groups). Further, only the multidomain group demonstrated a significant gain in far-transfer measures of attention.

Anguera et al. (2013) also demonstrated that multi-task training was more effective than single-task training for older adults in their videogame, *Neuroracer*. The multi-task training group exhibited improved sustained attention and WM performance and neural efficiency compared to the single-task training group and a passive control group. Additionally, after training, the multi-task group demonstrated comparable behavioral performance and neural activity on some aspects of the trained task to that demonstrated by untrained young adults. This is consistent with the findings of Das and Steyvers (2023) who modelled cognitive performance of cognitive training users, indicating that training may help older adults “catch up” to young adults on cognitive tasks. Including a young adult comparison group therefore provides an important benchmark for evaluating the extent of training-related gains in older adults, clarifying whether improvements reflect meaningful restoration of performance toward younger adult levels. Such evidence would strengthen claims about the potential of cognitive training to counteract age-related decline and promote resilience in aging.

Despite these promising findings, previous work has not specifically targeted core EFs and directly compared single-domain WM training with multidomain EF training in older adults, nor has it tested whether such training can elevate performance toward that of younger adults. The present study therefore examined the extent of behavioral transfer across older adult training groups and benchmarked their post-training performance against a group of untrained young adults.

Furthermore, despite self-perceptions of aging and cognitive functioning having significant implications for promoting healthy cognitive and physical aging (e.g., Cosentino et al., 2018), these outcomes have been overlooked in the cognitive training literature. Subjective measures of functioning are essential to consider as self-appraisals of cognitive abilities and affective states, such as mood and well-being, correlate with objective cognitive performance, physical health, and longevity (Goghari & Lawlor-Savage, 2018; Westerhof et al., 2023). These subjective beliefs therefore hold clinical importance for preserving functional independence in old age and are essential to evaluate in cognitive training studies (Nguyen et al., 2021). Thus, this study also included objective and subjective measures of transfer to elucidate the relationship between these measures in relation to cognitive training.

### The current study

The present study aimed to directly compare the efficacy of single-domain WM training and multidomain EF training (targeting WM, inhibitory control, and cognitive flexibility) in healthy older adults. By contrasting these two approaches, this study addresses a key gap in the literature, as no prior work has explicitly tested whether multidomain EF training produces broader or stronger transfer effects than WM training alone in this population. In addition to evaluating transfer effects on objective measures of cognition, we also examined subjective outcomes related to perceived cognitive functioning and well-being, which have often been overlooked despite their importance for healthy aging. Finally, by including a comparison with untrained young adults, the study provides novel insights into whether cognitive training can support meaningful restoration of cognitive performance toward levels observed in younger adults. This study contributes to the literature by clarifying the conditions under which cognitive training promotes transfer, advancing theoretical understanding of EF training, and informing the design of interventions that support cognitive health and well-being in aging.

Based on these aims and the existing literature, the following hypotheses were proposed:

- Multidomain EF training will be effective in improving performance on trained and transfer (objective and subjective) outcomes compared to an active control group.
-  After training, performance on cognitive tasks in the multidomain EF training group will be comparable to that of untrained young adults.
- Single-domain WM training will be effective in improving performance on memory-related (objective and subjective) outcomes compared to an active control group.
- After training, performance on memory measures in the WM training group will be comparable to that of untrained young adults.
- Multidomain EF training will elicit larger improvements in objective and subjective outcomes compared to single-domain WM training in older adults.

## Method

### Participants

Participants were recruited through community advertising, with posters and flyers displayed in public community areas (e.g., shopping centers, retirement villages, independent living communities). Interested individuals were invited to register their interest by completing a contact form provided with the flyer. A researcher then contacted prospective participants to provide further information about the study and to conduct a brief initial screen (e.g., self-reported cognitive impairment, mobility restrictions). Eligible individuals were subsequently invited to an in-person screening session, during which they completed assessments for colorblindness (Ishihara Test; Ishihara, 1918), cognitive impairment (Mini-Mental State Examination [MMSE]; Folstein et al., 1975; cut-off criterion < 27), and depression (Geriatric Depression Scale [GDS-15]; Yesavage & Sheikh, 1986; cut-off criterion < 8, e.g., Lampit et al., 2015; Park et al., 2009). Of the individuals who expressed interest, one was not enrolled due to suspected cognitive impairment based on confusion during the initial screening call. The final sample comprised 66 community-dwelling, healthy older adults (60 + years)^2^ with no physical or cognitive impairment (see Table 1 for a summary of participant characteristics). A gift voucher (AUD$20) was offered to participants in appreciation of their time participating in the study.

**Table 1:** Descriptive statistics and group comparisons on demographic and control measures for older adults

					Group Differences	
Variable	Total (*n* = 66)	EF (*n* = 22)	WM (*n* = 22)	AC (*n* = 22)	*Bayes Factor (error %)*	Interpretation
Age (years)	69.58 (7.04)	69.86 (7.19)	69.27 (7.09)	69.59 (7.17)	*BF*_incl_ = 0.13 (0.007%)	Substantial evidence for no difference
Gender (% male)	51.5%	59.1%	50.0%	45.5%	*BF*_incl_ = 0.17	Substantial evidence for no difference
Handedness (% right-handed)	97.0%	95.5%	100.0%	95.5%	*BF*_incl_ = 0.05	Strong evidence for no difference
MMSE (/30)	29.80 (0.40)	29.73 (0.46)	29.77 (0.43)	29.91 (0.29)	*BF*_incl_ = 0.32 (0.009%)	Substantial evidence for no difference
GDS (/15)	2.65 (1.10)	3.05 (1.46)	2.45 (0.86)	2.45 (0.80)	*BF*_incl_ = 0.64 (0.010%)	Ambiguous evidence for no difference
Education (years)	13.33 (2.79)	13.23 (2.18)	13.59 (3.43)	13.18 (2.72)	*BF*_incl_ = 0.14 (0.007%)	Substantial evidence for no difference
QPAR	48.06 (19.34)	46.55 (22.26)	47.73 (20.54)	49.91 (15.30)	*BF*_incl_ = 0.14 (0.007%)	Substantial evidence for no difference
Technology Confidence (/10)	7.08 (2.06)	6.73 (2.23)	7.36 (1.94)	7.14 (2.03)	*BF*_incl_ = 0.19 (0.008%)	Substantial evidence for no difference
Simple Reaction Time (ms)	371 (63)	370 (70)	374 (67)	369 (54)	*BF*_incl_ = 0.13 (0.007%)	Substantial evidence for no difference

Descriptive values represent mean and standard deviation, or frequency percentage. Bayes Factors (*BF*_incl_) presented to evaluate differences across groups. *QPAR* = Quick Physical Activity Rating scale, *GDS =* Geriatric Depression Scale, *MMSE* = Mini-Mental State Examination

A group of 33 untrained young adults (*M*_*age*_ = 22.42 years, *SD* = 5.14, range = 18–35; 60% females) was also recruited to compare cognitive performance across age groups. Young adult participants were undergraduate students recruited from a research participation pool who received partial course credit for participation. The testing of young adults’ performance on cognitive tests was conducted separately from the cognitive training study (but during the same time period). Ethical approval for this study was obtained from Griffith University’s Human Research Ethics Committee (GU Ref No: 2022/064).

### Study design and protocol

A pre-test—intervention—post-test (with 1-month follow-up) design was utilized for cognitive training of the older adult participants. Participants who expressed interest in the study attended a screening session at Griffith University. Those who were eligible were assigned to a training condition and completed the pre-test outcome measures. The method of assignment applied simple randomization by obtaining a randomized list of groups (using a free online program: https://www.random.org/lists/). Based on the a priori sample size analysis, the list included 22 instances of each condition: (i) multidomain-EF, (ii) WM-only, (iii) active control. As eligible participants signed up for the study, they were assigned to the next condition on the list. Exceptions occurred where participants within couples or groups (e.g., social group, living community) participated simultaneously, in which case, they were allocated to the same condition to strengthen the blinding of conditions within the study.^3^

At the end of the pre-test session, participants were provided with an 11-inch computer tablet to complete the training and a printed journal to record the details of their training (session start and end time, checklist of games to play each session). The journal also included questions about perceived level of enjoyment, motivation, and challenge at the end of each training week. The researcher demonstrated tablet use and explained each training game to ensure participants understood the training requirements. After completing the 4-week training program, participants completed the post-test measures. Each testing session lasted approximately 1.5–2 h. An online survey comprising the subjective outcomes was sent to participants one month following post-test completion.

**Fig. 1:**
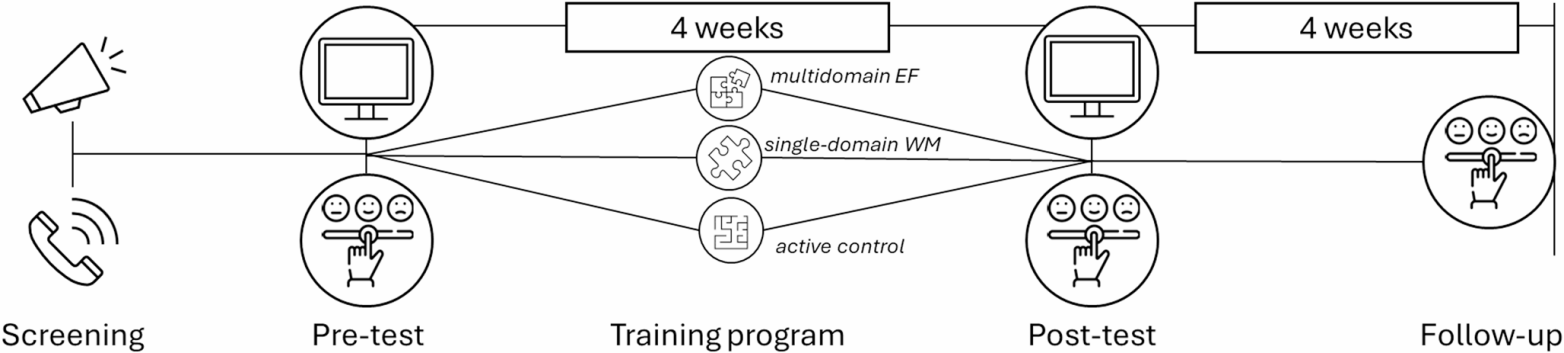
Schematic overview of study design. Note. After expressing interest in the study, participants were contacted via telephone to undertake a brief screening to determine eligibility. Eligible participants underwent pre-test (computerized cognitive tasks and self-report survey questions), followed by a 4-week intervention (either multidomain EF training, single-domain WM training, or active control), then post-test (computerized cognitive tasks and self-report survey questions). Four weeks after post-test, participants were sent an email to complete the self-report survey questions online

Adherence was facilitated by several features of the study design, including weekly researcher contact (check-in calls/texts, depending on participants’ preference), use of training journals (to log sessions), accessible support channels (researcher contact details prominently displayed in training app and training journal), provision of a loan tablet to complete training intervention on (participants were required to return), and the relatively short 4-week duration of the program. No participants withdrew from the study. Figure 1 presents a schematic overviewing the study design.

### Training programs

Based on previous meta-analyses regarding optimal doses for eliciting cognitive training and transfer effects for older adults (e.g., Lampit et al., 2014; Nguyen et al., 2019), each training program comprised 9 h of training spanning 4 weeks (45 min sessions, 3 sessions/week). Participants were given flexibility regarding the specific days and times for completing their training sessions. All participants adhered to this schedule. One participant completed their post-test assessment after 6 weeks (rather than 4) due to travel, but all training sessions were completed.

#### EF training program

The EF training program was designed and developed by the researchers and involved gamified versions of EF tasks to make the training more appealing (e.g., colorful animations, scoring, engaging interfaces). Each game was independently adaptive in difficulty, utilizing the staircase algorithm as recommended by Mishra et al. (2013) (i.e., increase a level after ≥ 85% of trials were correct, or decrease a level after ≥ 15% of trials were incorrect). As summarized in Table 2, this adaptivity was operationalized differently across domains (e.g., increasing stimuli to remember in WM tasks, reducing time to respond and increasing interference in inhibitory control tasks, and increasing switch frequency in cognitive flexibility tasks). Beyond tick symbols (for correct responses) and cross symbols (for incorrect responses), no other explicit feedback was provided to participants; rather, progression through levels served as implicit feedback. The final level reached in one session became the starting level in the next. For further details regarding the design and development of the program, see Nguyen et al. (2025).

**Table 2:** Brief descriptions of the EF training games

Cognitive Domain	Game Name	Increasing Difficulty	Brief Description
Working Memory	Dynamic Doors	More objects to remember and update; less time to respond	Based on the *n-*back (WM updating), players must determine whether the current object matches the object presented *n* trials before it. As the difficulty increases, *n* (the number of objects that players must maintain and update in working memory) increases. For instance, easier levels start with the 1 back where players must determine whether the object presented matches the object from one trial ago.
	Treasure Trail	Larger grid size; more stimuli to remember	Based on the Corsi Block task (visuospatial WM), a grid of cells is initially presented. Within certain cells, a dragon, treasure chest, knight, or nothing is shown. The contents of cells are then hidden. Players are prompted to select stimuli in a specific order (e.g., locate all knights, then dragons, then chests). As game difficulty increases, the grid becomes larger and there are more of each stimulus type to remember.
	Puzzling Potions	Longer sequence length	Based on WM span tasks, six different colored potions are aligned in a row. Colored potions will sequentially ‘glow’. After the sequence has completed, the location of each potion is randomly shuffled along the row. Players must tap on the potions in the same order in which they glowed (based on the color rather than the initial location of the potion). The sequence length increases as players progress through the levels.
Inhibitory Control	Celestial Colors	Stimuli with more interference; less time to respond	Based on the Stroop task, players are asked to determine whether the ink color of the stimulus shown matches a specific color. Stimuli include shapes (no interference), non-color words (e.g., PITCH), and color words (e.g., PURPLE – high interference). As the difficulty increases, players must appraise stimuli that involve higher levels of interference and receive less time to respond.
	Meteor Madness	Stimuli with more interference (more similarity between go and no-go stimuli); less time to respond (stimuli move faster)	Based on the Go/No-Go task, objects move across the screen from right to left. Some objects are meteors whilst some are aliens. Players must tap on the meteors (go) before they move to the other side of the screen and avoid tapping on the aliens (no go). As players progress through the levels, the speed at which objects move across the screen increases. Additional types of aliens (similar in color to the meteors) also appear.
	Roaming Rockets	More interference (added distractors with similar appearance to target); less time to respond	Based on the Flanker task, a row of three to five objects appears on the screen. The middle object is always a rocket. Players must swipe the screen in the direction in which the middle rocket is pointing in. The game begins with little interference (rocket flanked by four meteors) and increases in difficulty by introducing more and more interference on the screen (rocket flanked by other rockets, rockets will start to move, other unrelated pointed objects [missiles] will fly across the screen).
Cognitive Flexibility	Baffling Biscuits	More tasks to switch between; higher task switch frequency; less time to respond	Based on the cued-task-switching paradigm, players are presented with a biscuit with a colored letter-number combination (e.g., 8 K [in red text]) printed on it. This biscuit will appear under one of three columns: letter, number, color. Each column defines a rule which is used to appraise the letter-number combination: • Letter: is the letter a vowel? • Number: is the number odd? • Color: is the color red? For example, if 8 K was presented under the Letter column, players must press ‘no’ as the letter in this letter-number combination does not have a vowel. Only one rule is active at a time during the easiest levels (no switch). As the difficulty increases, more rules become active whereby biscuits could appear under any of the active rules (switch), and less time is provided to give a response.
	Ludicrous Lollies	Higher task switch frequency; more interference (more distractors compared to target stimuli); less time to respond	Based on set-shifting tasks, candies appear on the screen, all pointing and moving in the same direction (though, the orientation and direction of movement are not always congruent). When the candies are pink, players must swipe the screen in the direction that the candies are moving (ignoring the pointing direction). When the candies are orange, players must swipe in the direction that the candies are pointing (ignoring direction of movement). As players progress through the levels, distractor candies are presented on the screen (first stationary, then moving in a random direction) and time to respond is reduced.
	Striking Sweets	More response options; higher rule switch frequency; less time to respond	Based on the Arrows and Colors Cognitive Test, a target candy of a specific color and orientation is presented (e.g., blue candy pointing left). An array of candies is presented below this target. Above the target is a rule (e.g., same color, different direction). Players must select the candy from the array that adheres to the rule, with reference to the target candy (i.e., a candy within the array that is blue but not pointing left). Only one candy in the array will be correct. This rule changes throughout the game (same color, same direction; same color different direction; different color, same direction; different color, different direction). As the difficulty increases, more candies appear in the array (ranging from two to five), the rule will change more often, and less time is provided to give a response.

The *multidomain EF program* comprised three sets of mini-games, with each set targeting a different core EF (WM, inhibitory control, cognitive flexibility). Each set comprised three mini-games. Participants assigned to this program were asked to focus on one set (domain) of games each session and to cycle through each set throughout the week (e.g., WM-Session 1, inhibitory control-Session 2, cognitive flexibility-Session 3). Each mini-game lasted 3 min, and participants were asked to play each game 5 times during each training session (constituting 45 min of training per session). The *single-domain WM program* only included the three WM games from the multidomain EF program. Participants assigned to this program were asked to play the same three WM games each session. The other sets of games were not visible in the WM training app. Table 2 presents an overview of the games within the EF training program.

#### Active control program

The active control (AC) game program was designed to elicit the same experience and non-focal effects (e.g., enjoyment, motivation, expectancy to improve) as the EF and WM training programs, but without the cognitive ‘ingredient’ of interest—EFs (Schmiedek, 2021). Three sets of mini-games were included in the AC program, with each set comprising three mini-games involving number-based puzzles, word-based puzzles, and visuospatial-based puzzles. These genres were chosen in accordance with findings from Boot et al. (2016), who found that older adults reported such games to be enjoyable and as challenging as “brain training” games. The games for this program required minimal usage of core EFs^4^ and increased in difficulty as the game progressed (though the difficulty was not adaptive). Games were sourced from the Google Play store. See Supplementary Materials (Table S1) for an overview of each game in the AC training program.

### Measures

#### Control measures

As cognitive reserve, physical activity, confidence using technology, expectancy effects, and processing speed have been linked to cognitive performance and/or cognitive training gains, these outcomes were measured to ensure parity across training groups on these factors. Years of education was used as a proxy for cognitive reserve (e.g., Dorbath et al., 2013). Physical activity was assessed using the Quick Physical Activity Rating (QPAR) scale (Galvin et al., 2020). Technological confidence was assessed by asking participants about their confidence in using technology (1 = *not at all confident* to 10 = *very confident*). Participants’ expected cognitive benefits from numerous popular activities (including cognitive/brain training, crosswords, physical exercise, etc.) were assessed using items from the perceptions of brain training survey (Ng et al., 2020) (i.e. participants were asked to rate how helpful they believe each activity is for improving cognitive functioning from 1 = *not at all helpful* to 5 = *extremely helpful*). Processing speed was assessed using a computerized Simple Reaction Time task in which participants were required to press the spacebar as soon as the target stimulus (blue star) appeared on the screen. In each trial, the stimulus appeared on a black background after a random delay interval (ranging from 1,000 to 3,000 ms). The stimulus remained on the screen until participants responded or until 3 s had elapsed (whichever occurred first). The task comprised 5 practice trials followed by 30 experimental trials. The outcome assessed was average reaction time (ms).

#### Training-Experience related outcomes

To assess participants’ experience of the intervention, user-experience and perceived improvements were assessed via an online questionnaire at post-test.

##### **Game User Experience Satisfaction Scale (GUESS)**

Three subscales from the GUESS (Phan et al., 2016) were administered to explore participants’ experience of the cognitive training program in relation to enjoyment (perceived fun), usability/playability (perceived ease of use and clean user interface), and gratification (perceived sense of accomplishment and desire to succeed). These subscales demonstrated strong internal consistency in the current study (enjoyment α = 0.91, usability/playability α = 0.90, gratification α = 0.82), and in previous research (α = 81, 0.84, 0.77, respectively; Phan et al., 2016). Responses (ranging from 1 = *strongly disagree* to 7 = *strongly agree*) were averaged for each subscale due to the unequal number of items in each subscale (enjoyment = 5, usability/playability = 11, gratification = 6). Higher scores represent higher satisfaction with the training games.

##### **Subjective Training-Related Improvements**

Adapted from Boot et al. (2013) who originally assessed participants’ beliefs about the utility of training across perceptual and cognitive domains, the current study asked participants about the extent to which they believed that the training improved their: (i) memory, (ii) attention, (iii) perception, (iv) hand-eye coordination, (v) speed and reaction time, (vi) reasoning, (vii) multitasking ability, and (viii) everyday abilities. Responses were rated on a 5-point scale (1 = *strongly disagree* to 5 = *strongly agree*), with higher scores representing stronger belief of improvement.

#### Cognitive outcome tasks

The presentation order of the following cognitive tasks was randomized (using an online program: https://www.random.org/lists/) for each participant and each testing occasion to control for carryover and learning effects (Gobet & Sala, 2023). Cognitive outcomes were assessed at pre-test and post-test^5^ and tasks were run via the OpenSesame V3.2 program on a 17.3-inch laptop.

##### ***N*-Back Task (WM Updating**,** Trained Task)**

White-colored shapes (circle, diamond, pentagon, square, triangle) were presented sequentially in the center of a black background. Participants were asked to determine whether the current shape matched the shape presented *N* trials back (*N* ranged from 1 to 3). Response-mapping was counterbalanced across testing occasion and participants: the right arrow key was pressed for a match and the left arrow key for a non-match (or vice versa). Each condition (1-, 2-, 3-back) comprised 10 practice trials followed by two blocks of 40 trials (80 trials/condition, 240 trials total). The order of stimuli was pseudorandomized so that each block incorporated a 2:3 ratio of match to non-match trials. Stimuli were presented on screen for 500ms, followed by an interstimulus-interval (ISI) of 1,500ms regardless of whether a response was made. Performance was assessed by number of hits, correct rejections, misses, false alarms, and reaction time. Use of the discrimination index (derived from signal detection theory) as an outcome measure of the *N*-back task has been widely recommended as this index accounts for most of the aforementioned performance indices during the task (Meule, 2017). The non-parametric discrimination index (*A՛*) was therefore calculated. Similar to past cognitive training research (Tusch et al., 2016; Vermeij et al., 2017), a Composite *A՛* score was computed (using *A՛* and mean reaction time in response to target stimuli) to account for speed/accuracy trade-offs (see Vermeij et al., 2017 for detailed formulas). The Composite *A’* score was calculated for each *N-*back condition, with higher scores reflecting better *N*-back performance.

##### **Flanker Task (Inhibitory Control**,** Trained Task)**

On each trial, a row of five white arrows pointing left or right was presented in the center of the screen (on a black background). Participants indicated the pointing direction of the middle arrow (using the left or right arrow key) whilst ignoring the flanking arrows. The flanking arrows either pointed in the same direction as the middle arrow (congruent trial) or in the opposite direction relative to the middle arrow (incongruent trial). There were 10 practice trials followed by 120 trials (3 blocks of 40 trials) which were presented in a pseudorandomized order to ensure a 2:3 congruent-to-incongruent ratio. Each trial presented a fixation cross for 200ms, followed by the arrows for 500ms, and an ISI of 1,300ms. Only correct trials were used to calculate the Flanker Effect (RT_IncongruentTrials_ – RT_CongruentTrials_), with lower scores indicating better performance. Accuracy was not analyzed as an outcome due the presence of a ceiling effect in which participants, on average, achieved near perfect accuracy.

##### **Cued Task-Switching Paradigm (Cognitive Flexibility**,** Trained Task)**

This task was adapted from task-switching paradigms by Gajewski and Falkenstein (2012) and Karbach and Kray (2009). A letter-number combination (e.g., E7) was presented in white font in the center of a black background (letters used: A, E, I, U, G, K, N, P; numbers used: 2, 4, 6, 8, 3, 5, 7, 9). Participants were asked to complete two tasks: a letter-evaluation task (is the letter shown a consonant or a vowel? ) and a number-evaluation task (is the number shown odd or even? ).

During single-task blocks, participants only completed trials pertaining to one task (number-evaluation only or letter-evaluation only). In mixed-task blocks, trials involved both number- and letter-evaluation tasks. To indicate the task to be completed in each trial, a cue was presented prior to the letter-number combination (“NUMBER” for the number-evaluation task, and “LETTER” for the letter-evaluation task). Participants were asked to press the left arrow key for consonants and odd numbers, and the right arrow key for vowels and even numbers (or vice versa). Response-mapping remained consistent for single- and mixed-task blocks but was counterbalanced across participants and testing occasions. Participants first received two single-task blocks (letter task, then number task), followed by three mixed-task blocks—10 practice trials were presented prior to starting the first block of each task. Each block comprised 40 trials which were pseudorandomized to ensure a 1:1 ratio for odd/consonant to even/vowel trials during single-task blocks and a 1:1 ratio for switch to nonswitch trials during mixed-task blocks. Each trial started with a fixation cross in the center of the screen (200ms) followed by the cue (1,000ms). The stimulus (letter-number combination) was then presented for 2,500ms or until a response was made, followed by a delay of 200ms prior to the start of the next trial. The outcomes of interest included switch-costs (RT_SwitchTrials_ – RT_NonswitchTrials_ during mixed-task blocks) and mixing-costs (RT_MixedTask_ – RT_SingleTask_) (Kray et al., 2019). Lower scores (smaller switch/mixing-costs) reflect better performance. Due to the presence of a ceiling effect, no analysis was conducted on accuracy data.

##### **Wisconsin Card Sorting Task (WCST; Global Executive Functioning**,** Near-Transfer Task)**

On each trial, a row of four cards differing in color (red, green, blue, yellow), shape (triangle, cross, diamond, circle), and number of shapes (1, 2, 3, 4) was presented on a black background. Below this row of cards, a target card was presented. Participants were asked to sort the target card into one of the four decks above it by using a mouse to click on the card into which it should be sorted. Cards were sorted based on one of three rules (color, shape, or number of shapes), and the sorting rule changed after 10 consecutive correct responses. To determine how to sort the card, participants needed to use the feedback provided after each response (“CORRECT” for a correct sort, and “INCORRECT” for an incorrect sort). For each trial, the cards were presented until the participant responded and feedback was then provided for 1,000ms prior to the commencement of the next trial. A series of 10 practice trials was presented in which the sorting rule changed after 3 correct responses, followed by the experimental block comprising 64 trials. Outcomes measured included total accuracy, perseveration errors, and categories completed (Miles et al., 2021), where lower errors, higher accuracy, and more categories completed indicate better performance.

##### **Virtual Week (Prospective Memory**,** Far-Transfer Task)**

Virtual Week is a computerized task resembling a board game (Rendell & Craik, 2000). To move around the board, participants clicked the die in the middle of the board, simulating a die roll. Each circuit of the board represented one virtual day from 7:00AM to 10:00PM. In this study, a practice day in addition to three full virtual days were completed. Throughout each day, participants were required to complete tasks similar to activities performed in everyday life (e.g., taking medication, calling a friend, running errands). Tasks were to be performed either at specified events (after passing the relevant “Event” square on the board, e.g., “do [task] during breakfast”) or at a specific time of the virtual day (e.g., “do [task] at 5:00PM”) or on the stop-clock (timer indicating how much real-time has elapsed since starting the virtual day, e.g., “do [task] after 2 minutes has elapsed”). These tasks were either regular (i.e., to be performed at the same time/event during each virtual day) or irregular (i.e., specific to a particular day). On each day, 10 tasks needed to be performed: 6 regular tasks (2 time-check tasks using the stop-clock, 2 event-based tasks, 2 time-based tasks) and 4 irregular tasks (2 event-based and 2 time-based tasks). To “perform” these tasks, participants needed to click the “Perform Task” button and select the required task from the list of tasks. The proportion of correct (on-time) responses for time-check, time-based, and event-based tasks were assessed, with higher accuracy indicating better performance (see Rendell and Henry (2009) for a review of Virtual Week).

##### **Cattell Culture Fair Intelligence Test (CFIT) – Scale 3 (Fluid Intelligence**,** Far-Transfer)**

Similar to previous studies exploring cognitive training for older adults (e.g., Borella et al., 2014; Maraver et al., 2022), fluid intelligence was measured using Scale 3 of the CFIT (Cattell & Cattell, 1960). This measure comprises four subtests: series (identify option that completes the series of abstract figures/patterns), classifications (identify two figures that differ from the rest), matrices (identify the option that completes the matrix of abstract figures/patterns), and conditions (identify the option where the relationship between the dot(s), lines, and figures is the same as the target image). Participants were required to select the correct answer(s) for each item within a specified time limit according to the test manual (ranging from 2.5 to 4 min). Two parallel forms (A and B) were utilized in the study and were counterbalanced across participants and testing occasions. The outcomes measured were accuracy (proportion correct) and completion (proportion of questions completed), with higher accuracy and completion rate reflecting better performance.

#### Subjective cognitive and Well-Being outcomes

The following outcome measures were administered to older adults using an online questionnaire at pre-test, post-test, and at a 1-month follow-up.

##### **Subjective Ratings of Cognitive Abilities**

Adapted from similar subjective measures of cognitive functioning (e.g., Boot et al., 2013; Goghari et al., 2020; Rabipour et al., 2018), participants were asked to rate how they perceived their cognitive abilities from 1 (*very poor*) to 10 (*excellent*) for each of the following domains: memory, attention, speed and reaction time, reasoning, multi-tasking, and everyday functioning. To ensure clarity, each domain was accompanied by a brief definition (e.g., attention was described as “your ability to concentrate or stay focused on tasks”). For each domain, higher ratings indicate more positive perceptions of that cognitive ability.

##### **Perceived Deficits Questionnaire (PDQ)**

The 20-item PDQ (Sullivan et al., 1990) was used to assess perceived cognitive deficits in prospective and retrospective memory, attention, and organization/planning. Responses were rated on a 5-point scale (0 = *never* to 4 = *almost always*) and summed to generate a score for each domain (4 items per domain; maximum 20), with higher scores reflecting more perceived deficits. This scale has demonstrated strong reliability in the current study (α = 0.85) and past research (α = 0.87; Henneghan et al., 2017).

##### **Control**,** Autonomy**,** Self-Realization**,** and Pleasure (CASP-19) Scale**

The CASP-19 (Hyde et al., 2003) is a self-report measure of quality of life in older adulthood, assessing control, autonomy, self-realization, and pleasure. The 19-item scale uses a 4-point response format (ranging from 0 = *never* to 3 = *often*) summed to calculate a total score (maximum 57) where higher scores reflect higher perceived quality of life. The CASP-19 demonstrated strong reliability within the current sample (α = 0.84) and has previously demonstrated strong reliability (α = 0.83 to 0.86) and validity (strong associations with the Life Satisfaction Index – Well-being; Kim et al., 2015).

##### **Geriatric Depression Scale (GDS-15)**

The GDS-15 was used to assess subjective mood (depression symptoms) in older adults (Yesavage & Sheikh, 1986). The 15-item scale utilizes a binary response format (0 = *no*, 1 = *yes*) summed to form a total score, where lower scores reflect fewer depression symptoms. The GDS-15 demonstrated adequate reliability in the current sample (α = 0.79), as well as adequate reliability (α = 0.75) and strong convergent validity in past research (e.g., Friedman et al., 2005).

### Statistical analyses

Bayesian inference was conducted to quantify the evidence for or against the presence of training and transfer effects (e.g., Goghari & Lawlor-Savage, 2017; Guye & von Bastian, 2017; Harrell et al., 2023). The inclusion Bayes Factor (*BF*_incl_) was reported, which compares the predictive adequacy of models that include a given effect (e.g., a main effect or interaction) with models that exclude that effect, averaged across the entire model space. The strength of evidence, expressed by the Bayes Factor (*BF*_incl_), was reported. A *BF*_incl_ > 3 provides substantial support for the presence of an effect, whereas a *BF*_incl_ < 0.33 provides substantial support for the absence of an effect (Table 3 provides common labels for interpretation of Bayes Factors, adapted from De Simoni & von Bastian, 2018; Wagenmakers et al., 2018).^6^

**Table 3:** Labels for interpreting Bayes factors adapted from De Simoni and von Bastian (2018) and Wagenmakers et al. (2018)

Bayes Factor (BF_incl_)		Interpretation
Presence of Effect	Absence of Effect	
> 100	< 0.01	Decisive Evidence
30.01–99	0.01–0.03	Very Strong Evidence
10.01–30	0.04–0.10	Strong Evidence
3.01–10	0.11–0.32	Substantial Evidence
1.01–3	0.33–0.99	Ambiguous Evidence
1	1	No Evidence

One-way analyses of variance (ANOVAs) and chi-squared tests were conducted to evaluate pre-test differences between groups. To examine cognitive training efficacy (Hypotheses 1a and 2a), data were analyzed through mixed-factorial ANOVAs, with group as the between-groups factor (EF [multidomain EF], WM [single-domain working memory], AC [active control]) and time as the within-subjects factor (pre-test, post-test, 1-month follow-up [for subjective outcomes only]). Change scores (e.g., post-test minus pre-test) were also assessed to determine whether any training programs elicited larger pre-post changes than others (Hypothesis 3). Additionally, one-way ANOVAs and planned group comparisons were conducted for cognitive outcomes to compare performance of each older adult training group (at pre-test and post-test) to untrained young adults (Hypotheses 1b and 2b). For ANOVAs, where there was at least substantial evidence for main effects and/or interactions, pairwise comparisons were further explored through Bayesian *t*-tests adjusted for multiplicity. Specifically, posterior odds were reported for Bayesian post-hoc comparisons as they reflect multiplicity-adjusted values (this statistic is the result of multiplying the prior odds by the uncorrected Bayes Factor, reflecting the relative probability of the models after observing the data; Goss-Sampson et al., 2020).

Bayesian analyses were conducted using JASP Version 0.18.1.0 (JASP Team, 2023). Bayes Factors were computed using the widely accepted default prior settings for ANOVAs (fixed effects: *r* = .50, random effects: *r* = 1.00, scale covariates: *r* = .354) and *t*-tests (Cauchy prior with a medium scaling factor, *r* = .707) (Rouder et al., 2012) as these parameters have been used in previous cognitive training studies (e.g., Goghari & Lawlor-Savage, 2017; Guye & von Bastian, 2017; Katz et al., 2018; Ripp et al., 2022).

To minimize potential bias from participant attrition, all participants were included in the analysis based on the intention-to-treat principle (McCoy, 2017). Similar to previous cognitive training studies, the expectation-maximization method was used to impute missing data (Nouchi et al., 2016). This work was pre-registered on the Open Science Framework registry prior to data collection and updated prior to data analysis: 10.17605/OSF.IO/PZJAQ. De-identified data are linked to the project on the Open Science Framework repository: 10.17605/OSF.IO/KBFX3. Please refer to the Supplementary Materials for all statistics including any statistics not reported here (e.g., additional descriptive statistics, post-hoc tests, Frequentist statistics [e.g., *p*-values, *F-*statistics], baseline performance, and pre-post change scores).

## Results

### Participant and group characteristics

No differences were observed between training groups for any demographic or participant characteristics (see Table 1), or performance/scores at pre-test (see Supplementary Materials, Table S2). In general, participants’ perceptions of the usefulness of brain training games in improving cognitive functioning (prior to training) were moderately positive. Overall, participants perceived physical exercise to be the most helpful activity and action video games to be the least helpful. No group differences in ratings were evident across any activities (see Supplementary Materials, Table S3).

Each participant completed all training and testing sessions; however, one participant in the EF group did not complete the follow-up survey. The subjective follow-up data were therefore imputed for this participant. The average time between pre- and post-test sessions was 32.92 days (*SD* = 3.07), with all participants completing post-test four weeks after pre-test, except for one participant in the EF group who completed post-test after six weeks (due to travel).

### Perceptions of training

**Fig. 2:**
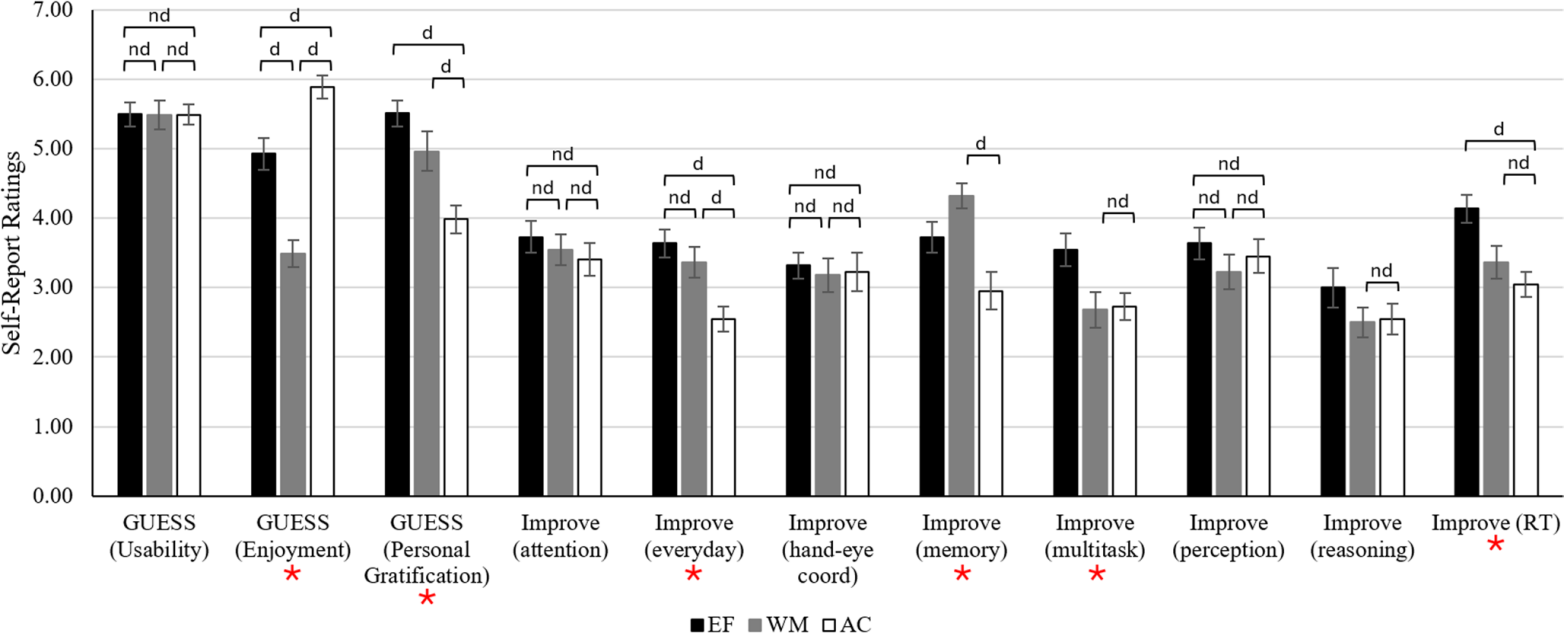
Perceptions of training across training groups. Note. d = at least substantial evidence for difference between groups (BFincl ≥ 3), nd = at least substantial evidence for no difference between groups (BFincl ≤ 0.33). Unmarked comparisons indicate ambiguous evidence. EF = multidomain EF group, WM = single-domain WM group, AC = active control group. Error bars represent 95% confidence interval. Outcomes marked with an asterisk (*) indicate at least substantial evidence for group differences

Figure 2 summarizes participants’ perceptions of training across training groups. See Supplementary Materials for ANOVA statistics (Table S4) and post-hoc group comparisons (Table S5).

#### Game user experience satisfaction scale (GUESS)

A one-way ANOVA was run for each subscale of the GUESS to explore group differences in experiences of the training program. Findings revealed substantial evidence for no group differences in the ratings of *usability/playability* of the assigned training program. There was decisive evidence for group differences in the *enjoyment* of the training program, with the AC group reporting higher levels of enjoyment than both the EF and WM groups. The EF group also reported higher enjoyment than the WM group. Decisive evidence for group differences was also observed in *personal gratification* derived from training, with the AC group reporting lower gratification than both EF and WM groups, and ambiguous evidence for no difference between the EF and WM groups.

#### Subjective Training-Related improvements

To examine group differences in perceptions of training-related cognitive improvement at post-test, one-way between-groups ANOVAs were conducted for each domain: attention, everyday abilities, hand-eye coordination, memory, multitasking, perception, reasoning, and speed/reaction time. There was substantial evidence for no group differences in the domains of attention, hand-eye coordination, perception, and reasoning.

There was very strong evidence for group differences in perceived training-related improvements in everyday abilities, such that the AC group reported less improvement than both EF and WM groups. There was no difference between the EF and WM groups.

Additionally, there was strong to very strong evidence for group differences in perceived training-related memory improvements and improvements in speed/reaction time. The WM group reported stronger memory improvements than the AC group; however, there was ambiguous evidence for differences between the EF and both WM and AC groups.

Similarly, whilst larger perceived multitasking improvements were reported in the EF group, there was ambiguous evidence for differences between the EF group and both WM and AC groups. There was no difference between the WM and AC groups.

### Group differences across cognitive outcomes

**Fig. 3:**
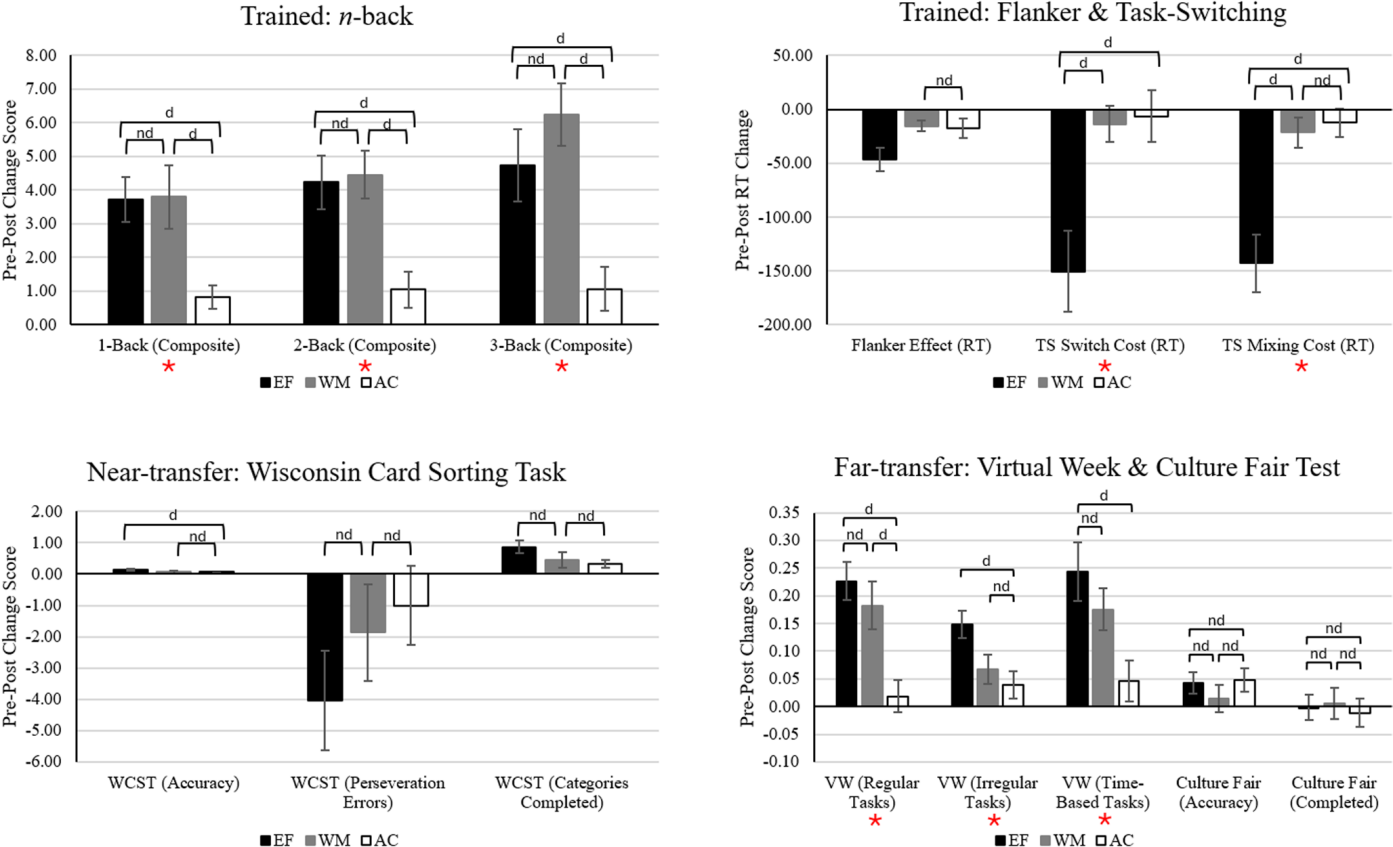
Between-groups comparisons of pre-post change scores across cognitive tasks. Note. d = at least substantial evidence for difference between groups (BFincl ≥ 3), nd = at least substantial evidence for no difference between groups (BFincl ≤ 0.33). Unmarked comparisons indicate ambiguous evidence. EF = multidomain EF group, WM = single-domain WM group, AC = active control group. Error bars represent 95% confidence interval. Negative change scores for reaction times (RT) and error indices indicate improved performance. For all other outcomes, positive change scores reflect improved performance. Outcomes marked with an asterisk (*) indicate at least substantial evidence for a time x group interaction

Analysis of pre-post training changes for cognitive measures was conducted using a 2 Time (pre-test, post-test) × 3 Group (EF, WM, AC) × 3 Condition (1-back, 2-back, 3-back) ANOVA for the *N*-back task. For other cognitive outcome measures, 2 Time (pre-test, post-test) × 3 Group (EF, WM, AC) ANOVAs were conducted. Summary statistics are presented in Table 4 (descriptive statistics) and Table 5 (inferential statistics). Group differences across tasks are illustrated in Fig. 3. See Supplementary Materials for within-groups pre-post change statistics (Table S6) and between-groups change comparisons across cognitive tasks (Table S7).

**Table 4:** Descriptive statistics for cognitive outcome measures across older adult training groups and untrained young adults

Outcomes	Young Adult Group	EF Group		WM Group		AC Group	
		Pre-Test	Post-Test	Pre-Test	Post-Test	Pre-Test	Post-Test
***N*** **-Back Task**							
1-Back (Composite)	12.96 (6.74)	8.20 (3.39)	11.77 (2.61)	7.43 (2.16)	11.22 (3.72)	7.92 (2.66)	8.74 (2.12)
2-Back (Composite)	10.26 (6.27)	5.42 (3.37)	9.32 (3.51)	4.82 (2.16)	8.92 (2.40)	5.00 (2.01)	5.85 (2.61)
3-Back (Composite)	9.87 (6.00)	3.86 (2.63)	8.05 (3.41)	3.00 (2.17)	7.53 (3.02)	4.12 (1.86)	4.16 (2.07)
**Flanker Task**							
Flanker Effect (RT, ms)	42 (14)	99 (55)	53 (26)	96 (52)	81 (43)	98 (66)	81 (61)
**Cued Task-Switching Paradigm**							
Switch Cost (RT, ms)	85 (80)	158 (44)	116 (30)	150 (43)	142 (45)	147 (38)	144 (37)
Mixing Cost (RT, ms)	201 (167)	411 (168)	268 (140)	378 (148)	356 (155)	377 (152)	365 (158)
**WCST**							
Accuracy (Proportion Correct)	0.82 (0.09)	0.64 (0.12)	0.78 (0.11)	0.66 (0.20)	0.72 (0.16)	0.62 (0.20)	0.64 (0.19)
Number of Perseveration Errors	6.61 (4.29)	13.68 (4.88)	9.64 (5.73)	11.68 (7.19)	9.82 (6.38)	12.55 (7.01)	11.55 (8.05)
Number of Categories Completed	4.45 (0.71)	3.18 (0.96)	4.05 (0.79)	3.23 (1.23)	3.68 (0.99)	3.18 (1.26)	3.50 (1.22)
**Virtual Week**							
Regular Tasks (Proportion Correctly Completed)	0.84 (0.17)	0.44 (0.23)	0.66 (0.17)	0.44 (0.15)	0.62 (0.19)	0.49 (0.15)	0.51 (0.16)
Irregular Tasks (Proportion Correctly Completed)	0.66 (0.19)	0.35 (0.17)	0.50 (0.11)	0.36 (0.17)	0.42 (0.11)	0.34 (0.17)	0.38 (0.12)
Time-Based Tasks (Proportion Correctly Completed)	0.59 (0.28)	0.35 (0.23)	0.59 (0.19)	0.37 (0.25)	0.55 (0.19)	0.46 (0.20)	0.51 (0.14)
**CFIT**							
Accuracy (Proportion Correct)	—	0.41 (0.09)	0.45 (0.08)	0.43 (0.08)	0.44 (0.09)	0.43 (0.07)	0.48 (0.08)
Completion (Proportion Completed)	—	0.72 (0.11)	0.72 (0.10)	0.72 (0.12)	0.73 (0.12)	0.72 (0.10)	0.70 (0.07)

Values represent mean and standard deviation

**Table 5:** Summary results for cognitive outcomes among older adult training groups

Outcomes	Interactions	Main Effects	Post-Hoc Comparisons	
***N*** **-Back Task**				
*N*-Back (Composite)	Time×Condition×Group: *BF*_incl_ = 0.07 – strong^nd^ Time×Group: *BF*_incl_ = 7.08 × 10^3^ – decisive^d^ Time×Condition: *BF*_incl_ = 0.06 – strong^nd^ Condition×Group: *BF*_incl_ = 0.03 – very strong^nd^	Condition: *BF*_incl_ = 3.12 × 10^28^ – decisive^d^ Time: *BF*_incl_ = 8.20 × 10^8^ – decisive^d^ Group: *BF*_incl_ = 5.60 – substantial^d^	EF improved WM improved AC no change (3back only)	EF = WM EF > AC WM > AC
**Flanker Task**				
Flanker Effect (RT, ms)	Time×Group: *BF*_incl_ = 2.32 – ambiguous	Time: *BF*_incl_ = 3.91 × 10^3^ – decisive^d^ Group: *BF*_incl_ = 0.31 – substantial^nd^	EF improved	WM = AC
**Cued Task-Switching Paradigm**				
Switch Cost (RT, ms)	Time×Group: *BF*_incl_ = 128.13 – decisive^d^	Time: *BF*_incl_ = 128.94 – decisive^d^ Group: *BF*_incl_ = 0.24 – substantial^nd^	EF improved AC no change	EF > WM EF > AC
Mixing Cost (RT, ms)	Time×Group: *BF*_incl_ = 2.55 × 10^3^ – decisive^d^	Time: *BF*_incl_ = 616.01 – decisive^d^ Group: *BF*_incl_ = 0.34 – ambiguous	EF improved	EF > WM EF > AC WM = AC
**WCST**				
Accuracy (Proportion Correct)	Time×Group: *BF*_incl_ = 1.95 – ambiguous	Time: *BF*_incl_ = 278.98 – decisive^d^ Group: *BF*_incl_ = 0.65 – ambiguous	EF improved	EF > AC WM = AC
Number of Perseveration Errors	Time×Group: *BF*_incl_ = 0.37 – ambiguous	Time: *BF*_incl_ = 4.51 – substantial^d^ Group: *BF*_incl_ = 0.19 – substantial^nd^	AC no change	EF = WM WM = AC
Number of Categories	Time×Group: *BF*_incl_ = 0.49 – ambiguous	Time: *BF*_incl_ = 847.64 – decisive^d^ Group: *BF*_incl_ = 0.28 – substantial^nd^	EF improved AC improved	EF = WM WM = AC
**Virtual Week**				
Regular Tasks (Proportion Correctly Completed)	Time×Group: *BF*_incl_ = 129.48 – decisive^d^	Time: *BF*_incl_ = 2.40 × 10^5^ – decisive^d^ Group: *BF*_incl_ = 0.24 – substantial^nd^	EF improved WM improved AC no change	EF = WM EF > AC WM > AC
Irregular Tasks (Proportion Correctly Completed)	Time×Group: *BF*_incl_ = 6.68 – substantial^d^	Time: *BF*_incl_ = 1.84 × 10^4^ – decisive^d^ Group: *BF*_incl_ = 0.48 – ambiguous	EF improved WM improved	EF > AC WM = AC
Time-Based Tasks (Proportion Correctly Completed)	Time×Group: *BF*_incl_ = 7.58 – substantial^d^	Time: *BF*_incl_ = 1.04 × 10^5^ – decisive^d^ Group: *BF*_incl_ = 0.17 – substantial^nd^	EF improved WM improved	EF = WM EF > AC
**CFIT**				
Accuracy (Proportion Correct)	Time×Group: *BF*_incl_ = 0.22 – substantial^nd^	Time: *BF*_incl_ = 6.93 – substantial^d^ Group: *BF*_incl_ = 0.32 – substantial^nd^	WM no change	EF = WM EF = AC WM = AC
Completion (Proportion Completed)	Time×Group: *BF*_incl_ = 0.16 – substantial^nd^	Time: *BF*_incl_ = 0.18 – substantial^nd^ Group: *BF*_incl_ = 0.14 – substantial^nd^	EF no change WM no change AC no change	EF = WM EF = AC WM = AC

Bayes Factors (*BF*_incl_) presented for each interaction and main effect. ^d^*BF*_incl_ > 3 (indicating at least substantial evidence for interaction or main effect), ^nd^*BF*_incl_ < 0.33 (indicating at least substantial evidence for no interaction or main effect). Post-hoc comparisons are reported only for contrasts where Bayes Factors provided at least substantial evidence. EF = multidomain EF group; WM = single-domain WM group; AC = active control group. Left post-hoc comparison column indicates pre-post change for each group. Right post-hoc comparison column indicates difference in pre-post change between groups. All descriptive statistics and post-hoc comparisons presented in Supplementary Materials

#### Working memory updating

There was strong evidence for no 3-way interaction for the *N*-back task. There was decisive evidence for a main effect of condition, with poorer performance on the 3-back compared to the 1-back (*Odds*_posterior_ = 2.84 × 10^28^) and 2-back (*Odds*_posterior_ = 4.11 × 10^9^), and for the 2-back compared to the 1-back condition (*Odds*_posterior_ = 1.61 × 10^16^). There was also decisive evidence for a Time × Group interaction. Only the EF and WM groups exhibited pre-post improvements across all *N-*back conditions (AC group ambiguous evidence for improvement on the 1-back and no change in the 2-back; substantial evidence for no change on 3-back).

Group differences in pre-post change scores were examined to determine if the magnitude of training gains differed. Training gains were larger for the EF and WM groups than for the AC group. There was substantial evidence for no difference in pre-post gains between the EF and WM groups (i.e., the magnitude of pre-post improvement was comparable for these two training groups).

#### Inhibitory control

For the Flanker task, there was ambiguous evidence for a Time × Group interaction (for descriptive statistics, see Supplementary Materials, Table S8). There was decisive evidence for a main effect of time, but not group, indicating a general diminishing of the Flanker Effect from pre- to post-test (*Odds*_posterior_ = 4.55 × 10^3^).

#### Cognitive flexibility

For both switch-costs and mixing-costs within the cued task-switching paradigm, there was decisive evidence for a Time × Group interaction (see Supplementary Materials Table S9 for descriptive statistics). Post-hoc analyses indicated reduced switch- and mixing-costs from pre- to post-test for the EF group (ambiguous evidence for no change for WM and AC groups). The magnitude of improvement (pre-post change) for switch- and mixing-costs was larger for the EF group compared to the WM and AC groups.

#### Global executive functioning

For the WCST, there was ambiguous evidence for Time × Group interactions across all outcomes: accuracy, perseveration errors, and categories completed. For each WCST outcome, there was substantial to decisive evidence for a main effect of time, but not group, indicating that participants generally increased on accuracy (*Odds*_posterior_ = 286.19) and categories completed (*Odds*_posterior_ = 927.37) and decreased perseveration errors (*Odds*_posterior_ = 3.83) from pre- to post-test.

#### Prospective memory

For the Virtual Week task, the proportion of regular, irregular, and time-based tasks completed on-time (accuracy) was examined (see Supplementary Materials Table S10 for descriptive statistics). For each task type, there was at least substantial evidence for a Time × Group interaction. Post-hoc analyses revealed the same pattern of findings across tasks, such that, only the EF and WM groups demonstrated pre-post improvements in accuracy. The magnitude of pre-post change in accuracy was not different between the EF and WM group for regular and time-based tasks (ambiguous evidence for no difference on irregular tasks). The EF group demonstrated larger improvements than the AC group for each task type, whereas the WM group demonstrated larger improvements than the AC group for regular tasks only.

#### Fluid intelligence

For the CFIT, there was substantial evidence for no Time × Group interaction for overall accuracy and completion rate. There was substantial evidence for a main effect of time for accuracy indicating that, generally, participants improved from pre-test to post-test (*Odds*_posterior_ = 4.51).

### Comparison of cognitive performance between young adults and older adults

**Fig. 4:**
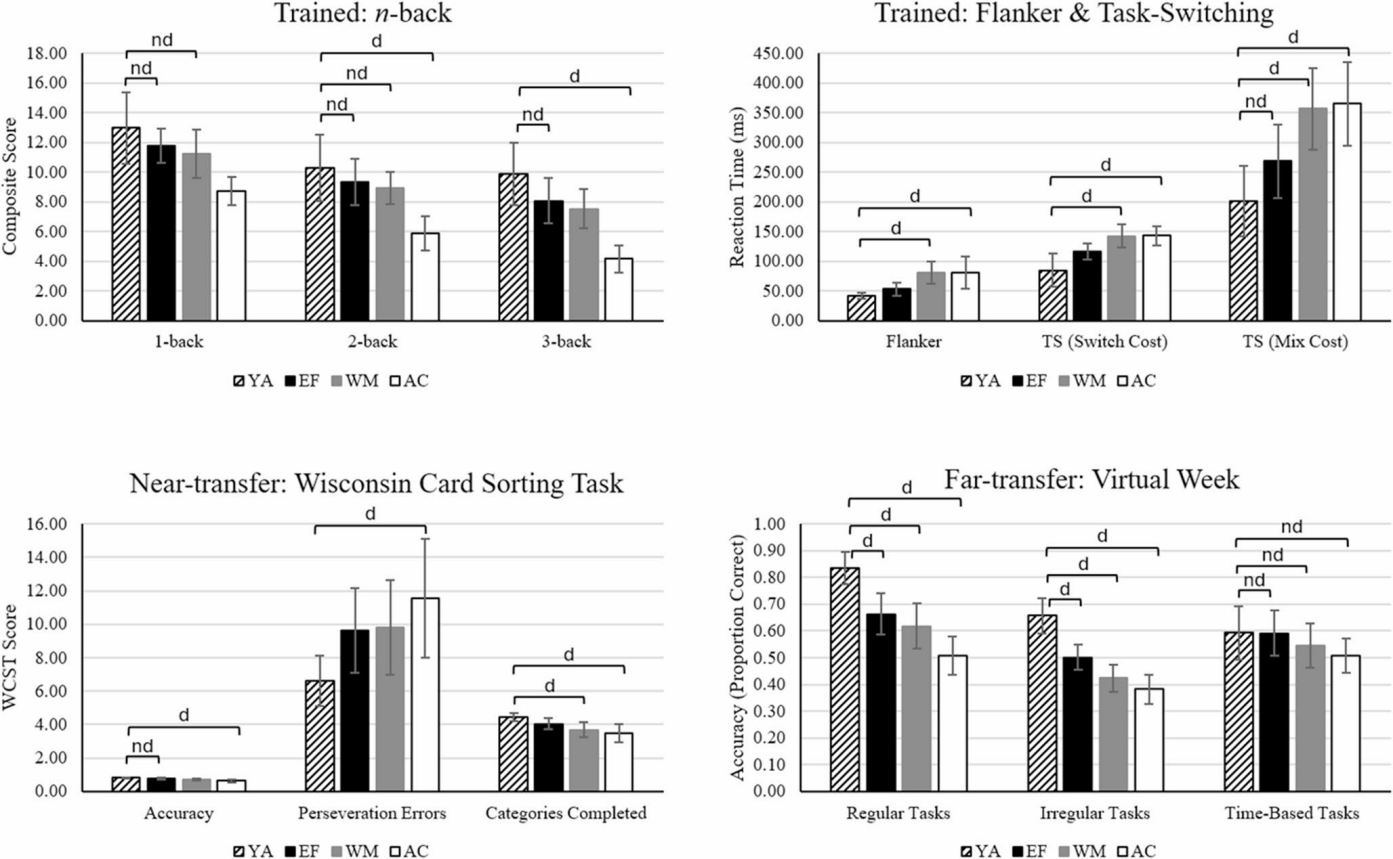
Comparison of older adults’ post-test cognitive performance to untrained young adults. Note. d = at least substantial evidence for difference between groups (BFincl ≥ 3), nd = at least substantial evidence for no difference between groups (BFincl ≤ 0.33). Unmarked comparisons indicate ambiguous evidence. YA = young adult group, EF = multidomain EF group, WM = single-domain WM group, AC = active control group. Error bars represent 95% confidence interval. Higher score for reaction times and errors reflect poorer performance. Higher scores on all other outcomes reflect better performance

One-way ANOVAs with planned group comparisons were conducted for each cognitive outcome to compare performance between each older adult training group and untrained young adults. See Fig. 4 for a visual summary of the group comparisons across tasks. See Supplementary Materials (Table S11) for post-hoc group comparisons.

#### Working memory updating

At pre-test, all older adult groups performed more poorly than young adults in each *N-*back condition. At post-test, the EF group demonstrated comparable performance to young adults on the 1-, 2-back, and 3-back conditions. The WM group also demonstrated comparable performance to young adults on the 1-back and 2-back conditions. On the 3-back condition, there was ambiguous evidence for no difference between WM and young adult groups. The AC group performed more poorly than young adults at post-test on the 2- and 3-back. There was ambiguous evidence for poorer performance in the 1-back condition.

#### Inhibitory control

All older adult groups exhibited larger Flanker Effects (poorer performance) than young adults at pre-test. At post-test, both WM and AC groups continued to exhibit larger Flanker Effects than young adults. There was ambiguous evidence for comparable performance between the EF group (at post-test) and young adults.

#### Cognitive flexibility

At pre-test, all older adult groups exhibited larger switch-costs and mixing-costs (poorer performance) compared to young adults. At post-test, the WM and AC groups maintained larger switch- and mixing-costs compared to young adults. In contrast, the EF group demonstrated comparable mixing-costs (at post-test) compared to young adults (ambiguous evidence for comparable switch-costs).

#### Global executive functioning

At pre-test, all older adult groups exhibited poorer performance than young adults on each WCST outcome (accuracy, perseveration errors, categories completed). At post-test, the AC group continued to demonstrate poorer performance on each WCST outcome compared to young adults. Similarly, the WM group maintained fewer categories completed (at post-test) than young adults. There was ambiguous evidence for comparable performance on perseveration errors and ambiguous evidence for poorer performance on accuracy. In contrast, the EF group demonstrated comparable performance to young adults on accuracy. There was ambiguous evidence for comparable performance in perseveration errors and categories completed.

#### Prospective memory

Accuracy (on-time completion) of each Virtual Week task type (regular, irregular, time-based) was lower across each older adult training group compared to the young adult group at pre-test. At post-test, each older adult group continued to exhibit poorer performance compared to young adults for regular tasks and irregular tasks. There was substantial evidence for comparable performance between each adult group and the young adult group on time-based tasks at post-test.

### Group differences in subjective outcomes

**Fig. 5:**
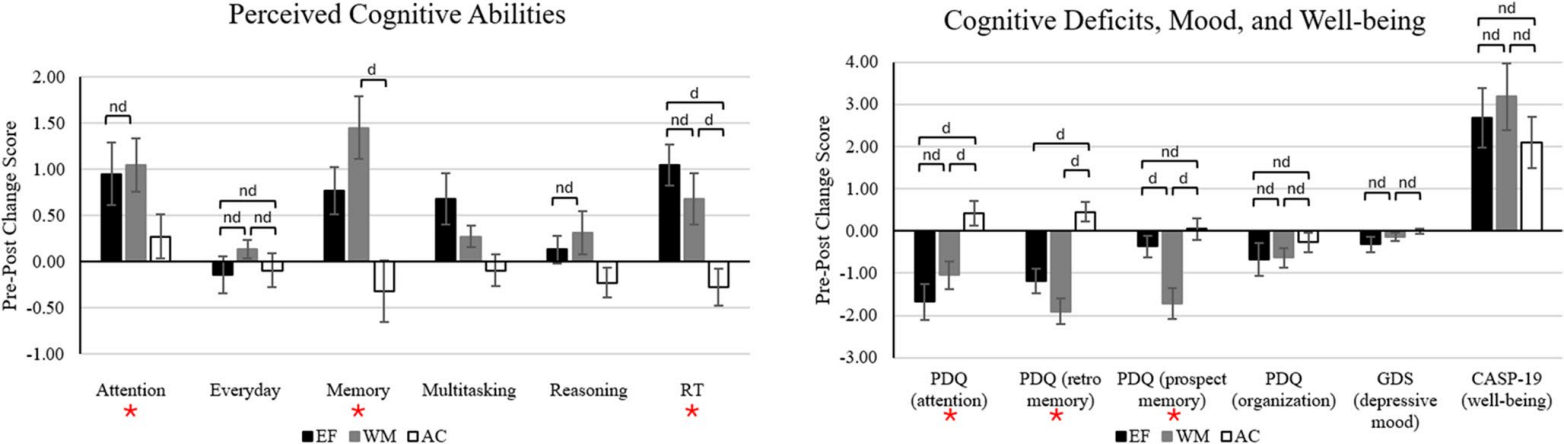
Between-groups comparisons of pre-post change scores across subjective outcomes. Note. d = at least substantial evidence for difference between groups (BFincl ≥ 3), nd = at least substantial evidence for no difference between groups (BFincl ≤ 0.33). Unmarked comparisons indicate ambiguous evidence. EF = multidomain EF group, WM = single-domain WM group, AC = active control group. Error bars represent 95% confidence interval. Outcomes marked with an asterisk (*) indicate at least substantial evidence for a time x group interaction

A series of 3 Time (pre-test, post-test, follow-up) × 3 Group (EF, WM, AC) ANOVAs were conducted to examine training group differences over the testing occasions for the subjective ratings of cognitive functioning, PDQ, CASP-19, and GDS-15. Table 6; Fig. 5 summarize the findings for subjective outcomes. See Supplementary Materials for within-groups pre-post/post-followup change statistics (Table S6) and between-groups change comparisons across subjective outcomes (Table S7).

**Table 6:** Summary results for subjective outcomes among older adult training groups

Outcomes	Interactions	Main Effects	Post-Hoc Comparison	
**Subjective Ratings of Cognitive Functioning**				
Attention	Time×Group: *BF*_incl_ = 5.41 – substantial^d^	Time: *BF*_incl_ = 9.95 × 10^3^ – decisive^d^ Group: *BF*_incl_ = 0.26 – substantial^nd^	EF pre-post increase WM pre-post increase	EF = WM (pre-post)
Everyday Tasks	Time×Group: *BF*_incl_ = 0.11 – substantial^nd^	Time: *BF*_incl_ = 0.06 – strong^nd^ Group: *BF*_incl_ = 1.02 – ambiguous	EF pre-post no change AC pre-post no change AC post-fu no change	EF = WM (pre-post) EF = AC (pre-post, post-fu) WM = AC (pre-post, post-fu)
Memory	Time×Group: *BF*_incl_ = 5.41 – substantial^d^	Time: *BF*_incl_ = 27.47 – strong^d^ Group: *BF*_incl_ = 16.35 – strong^d^	EF pre-post increase WM pre-post increase	EF = WM (post-fu) WM > AC (pre-post); WM = AC (post-fu)
Multitasking	Time×Group: *BF*_incl_ = 1.03 – ambiguous	Time: *BF*_incl_ = 0.38 – ambiguous Group: *BF*_incl_ = 0.49 – ambiguous	AC pre-post no change AC post-fu no change EF post-fu no change	EF = AC (post-fu)
Reasoning	Time×Group: *BF*_incl_ = 0.09 – strong^nd^	Time: *BF*_incl_ = 0.09 – strong^nd^ Group: *BF*_incl_ = 0.32 – substantial^nd^	EF pre-post no change AC post-fu no change	EF = WM (pre-post, post-fu) EF = AC (post-fu) WM = AC (post-fu)
Speed & RT	Time×Group: *BF*_incl_ = 11.77 – strong^d^	Time: *BF*_incl_ = 10.76 – strong^d^ Group: *BF*_incl_ = 70.46 – very strong^d^	EF pre-post increase EF post-fu decrease WM post-fu no change AC post-fu no change	EF = WM (pre-post, post-fu) EF > AC (pre-post); EF = AC (post-fu) WM > AC (pre-post); WM = AC (post-fu)
**PDQ**				
Attention	Time×Group: *BF*_incl_ = 43.20 – very strong^d^	Time: *BF*_incl_ = 438.65 – decisive^d^ Group: *BF*_incl_ = 0.41 – ambiguous	EF pre-post decrease EF post-fu no change WM pre-post decrease WM post-fu no change	EF = WM (pre-post, post-fu) EF > AC (pre-post); EF = AC (post-fu) WM > AC (pre-post); WM = AC (post-fu)
Retrospective Memory	Time×Group: *BF*_incl_ = 557.57 – decisive^d^	Time: *BF*_incl_ = 51.08 – very strong^d^ Group: *BF*_incl_ = 0.56 – ambiguous	EF pre-post decrease WM pre-post decrease WM post-fu no change AC post-fu no change	EF = WM (post-fu) EF > AC (pre-post); EF = AC (post-fu) WM > AC (pre-post); WM = AC (post-fu)
Prospective Memory	Time×Group: *BF*_incl_ = 13.10 – strong^d^	Time: *BF*_incl_ = 3.75 – substantial^d^ Group: *BF*_incl_ = 0.72 – ambiguous	WM pre-post decrease AC pre-post no change AC post-fu no change	WM > EF (pre-post); EF = WM (post-fu) EF = AC (pre-post, post-fu) WM > AC (pre-post); WM = AC (post-fu)
Planning / Organization	Time×Group: *BF*_incl_ = 0.06 – strong^nd^	Time: *BF*_incl_ = 3.19 – substantial^d^ Group: *BF*_incl_ = 0.32 – substantial^nd^	EF post-fu no change WM pre-post decrease WM post-fu no change AC post-fu no change	EF = WM (pre-post, post-FU) EF = AC (pre-post, post-FU) WM = AC (pre-post, post-FU)
**CASP-19**				
Total	Time×Group: *BF*_incl_ = 0.07 – strong^nd^	Time: *BF*_incl_ = 3.42 × 10^3^ – decisive^d^ Group: *BF*_incl_ = 0.31 – substantial^nd^	EF pre-post increase WM pre-post increase AC pre-post increase AC post-fu no change	EF = WM (pre-post, post-FU) EF = AC (pre-post, post-FU) WM = AC (pre-post, post-FU)
**GDS-15**				
Total	Time×Group: *BF*_incl_ = 0.12 – substantial^nd^	Time: *BF*_incl_ = 0.26 – substantial^nd^ Group: *BF*_incl_ = 0.64 – ambiguous	EF post-fu no change WM post-fu no change AC pre-post no change AC post-fu no change	EF = WM (pre-post, post-FU) EF = AC (post-FU) WM = AC (pre-post, post-FU)

Bayes Factors (*BF*_incl_) presented for each interaction and main effect. ^d^*BF*_incl_ > 3 (indicating at least substantial evidence for interaction or main effect), ^nd^*BF*_incl_ < 0.33 (indicating at least substantial evidence for no interaction or main effect). Post-hoc comparisons are reported only for contrasts where Bayes Factors provided at least substantial evidence. EF = multidomain EF group; WM = single-domain WM group; AC = active control group. Left post-hoc comparison column indicates pre-post change for each group. Right post-hoc comparison column indicates difference in pre-post change between groups. All descriptive statistics and post-hoc comparisons presented in Supplementary Materials

#### Subjective ratings of cognitive functioning

There was no Time × Group interaction or main effect of time or group for subjective ratings of everyday abilities and reasoning, and ambiguous evidence for an interaction concerning ratings of multitasking.

Time × Group interactions were observed for ratings of attention and memory. For attention, post-hoc analyses revealed higher ratings at post-test than pre-test in both EF and WM groups. There was ambiguous evidence for no pre-post change in the AC group, and mostly ambiguous evidence for no posttest-to-followup change in all three groups. The magnitude of pre-post change scores yielded mostly ambiguous evidence for no group differences. Similarly, for memory, both EF and WM groups reported better perceived memory at post-test than pre-test, and ambiguous evidence for no pre-post change in the AC group. Notably, the magnitude of pre-post change scores for memory ratings was larger in the WM group compared to the AC group. Other group comparisons concerning magnitude of change scores yielded mostly ambiguous evidence for no group differences.

There was also a Time × Group interaction for self-rated speed/RT abilities. Ratings of speed/RT increased from pre-test to post-test in the EF group; however, there was ambiguous evidence for change in the WM group and ambiguous evidence for no change in the AC group. Again, there was mostly ambiguous evidence for no posttest-to-followup change across groups. Comparison of group differences revealed that the magnitude of pre-post increase in self-rated speed/RT was comparable for EF and WM groups, and both groups demonstrated larger increases in pre-post ratings compared to the AC group.

#### Perceived cognitive difficulties (PDQ)

Time × Group interactions were observed for perceived deficits in attention, retrospective memory, and prospective memory. Post-hoc analyses yielded a similar pattern of findings for attention and retrospective memory, with reduced deficit scores from pre-test to post-test for both EF and WM groups (ambiguous evidence for no change in AC group). The magnitude of pre-post change scores was larger for EF and WM groups compared to the AC group. Perceived pre-post reductions in prospective memory deficits were clearly observed for the WM group (ambiguous and substantial evidence for no change in EF and AC groups, respectively). The magnitude of pre-post change scores was larger for the WM group compared to both EF and AC groups. There was mostly ambiguous evidence for no change from post-test to follow-up for each group.

There was no Time × Group interaction or main effect of group, but there was a main effect of time for perceived organization/planning deficits. Post-hoc analyses indicated that participants generally reported a reduction in organization/planning deficits from pre- to post-test (*Odds*_posterior_ = 6.59), with scores being maintained from post-test to follow-up (*Odds*_posterior_ = 0.08).

#### Well-being (CASP-19)

For the CASP-19 well-being score, there was no Time × Group interaction or main effect of group. There was decisive evidence for a main effect of time, where well-being scores generally increased from pre- to post-test (*Odds*_posterior_ = 6.87 × 10^5^); however, there was ambiguous evidence for no change from post-test to follow-up (*Odds*_posterior_ = 0.51).

#### GDS-15

For mood measured by the GDS-15, there was no interaction or main effects of group or time.

## Discussion

This study examined the efficacy of different cognitive training programs—multidomain core executive functions (EF), single-domain working memory (WM), active control (AC)—in improving objective measures of cognition and subjective measures of cognition and well-being in healthy older adults. Participants’ pre- and post-test cognitive performance was also compared to a sample of untrained young adults to evaluate the extent of potential improvements. Participants’ perceptions of training will be discussed, followed by the findings for the cognitive outcome measures (older adult training group comparisons, younger versus older adult comparisons), and the subjective outcomes.

### Perceptions of training

After completing the four-week program, participants reported their experience and beliefs about the efficacy of the training. Whilst perceptions regarding usability/playability were comparable across programs, the AC group reported substantially higher enjoyment but lower personal gratification ratings in relation to their program compared to the EF and WM groups. These findings are similar to that of Boot et al. (2016) who found that older adults playing control (non-cognitive training) games reported higher enjoyment of their program than those who engaged in a brain training program. This is unsurprising as non-cognitive training games are generally focused on entertainment and enjoyment and may not require substantial cognitive effort as such games may rely more on retrieval of existing (well-learned) knowledge from long-term memory, which is less effortful and less vulnerable to age-related decline (Todd et al., 2019). Consequently, games like those played by the AC group may offer a lesser sense of achievement and progress compared to the more challenging, adaptive EF and WM training programs.

Additionally, the EF group reported higher enjoyment than the WM group, which may reflect the greater variety of tasks offered through training across three EF domains, as opposed to the more repetitive, single-domain WM training. The diversity of cognitive challenges in the EF program may have contributed to sustained engagement and reduced task monotony, thereby enhancing participants’ enjoyment.

When asked to rate perceived improvements across cognitive domains, there were no group differences in attention, hand-eye coordination, perception, or reasoning. This may suggest that each training program targeted these domains equally (e.g., all programs required participants to attend to and perceive stimuli), thereby fostering comparable perceptions of gains in these areas. In contrast, the WM group reported stronger improvements in working memory and the EF group reported stronger improvements in speed and reaction time, likely due to the focus and requirements of the games within each training program (i.e., WM focusing on working memory-based tasks, and the EF games including more games involving time limits than both WM and AC programs).

Lastly, both EF and WM groups reported larger improvements to everyday abilities compared to the AC group. This is similar to Boot et al.’s (2013) findings that older adult participants who engaged in a cognitive training program reported that their training had the potential to benefit everyday abilities (more so than a non-cognitive training group), and is consistent with findings from a meta-analysis reporting that cognitive training elicits larger improvements in subjective everyday functioning compared to control groups among older adults (Nguyen et al., 2021). Overall, while older adults may find cognitive training less enjoyable than control games (e.g., entertainment games), it does appear to evoke a stronger sense of challenge, achievement, and subsequently, perceived improvements in a broad range of cognitive abilities.

It should, however, be noted that subjective measures, such as perceived improvements, may be susceptible to placebo (expectation) effects—perhaps due to response expectancy (Denkinger et al., 2021; Schwartz et al., 2016). Importantly, in this study, there was substantial evidence for no group differences in beliefs about the perceived benefits of any activity (including brain training, mobile/web games, and video games) prior to training, making it unlikely that expectation effects impacted these subjective findings. Whilst previous research has found no substantial evidence that placebo effects drive cognitive training-related improvements in objective cognitive outcomes, even when robust expectation-induction methods are used (e.g., Vodyanyk et al., 2021), the potential influence of expectation effects on subjective outcomes remains an important area for future research. Such research may help identify whether and how potential placebo/expectation effects can be minimized to better explore subjective outcomes, given the importance of such outcomes in contributing to older adults’ health, well-being, and outlook on their aging journey (e.g., positive aging; Wurm et al., 2017).

### Cognitive outcomes

Prior to discussing the cognitive findings, it is important to note that this study’s training program comprised multiple games/tasks within each cognitive domain. Whereas many cognitive training studies employ a single task per domain, our design drew on gamification and motivation frameworks to reduce monotony and sustain engagement over repeated sessions (Nguyen et al., 2025). Incorporating three games per domain was intended to not only reduce boredom and potential attrition, but also to target different subcomponents within each domain (e.g., WM updating, visuospatial WM, and WM span; cognitive and motor inhibition). This decision is also important for interpreting transfer effects, as outcome measures similarly engage multiple processes. For example, although the *n*-back is widely regarded as a WM updating task, efficient performance also depends on short-term capacity, maintenance, and manipulation; likewise, while the Flanker task primarily reflects cognitive inhibition, it also involves motor inhibition. This illustrates the well-documented *task-impurity problem*, whereby executive function tasks rarely isolate a single process and instead draw on overlapping constructs (Reuter-Lorenz et al., 2016). Accordingly, offering multiple training games per domain may better capture this complexity and increase the likelihood of transfer by engaging broader cognitive and neural systems (Guye et al., 2021), but it also means that observed improvements should be interpreted at the domain level rather than as gains in any single process (e.g., updating).

#### Group comparisons

##### **Trained Tasks**

As expected, the training programs elicited improvements on tasks from the same cognitive domain as the training tasks, with both EF and WM groups demonstrating pre-post improvements in the WM updating task, and the EF group also improving in the cognitive flexibility task. Despite having trained WM more frequently throughout the 4-week program, the WM training group’s improvement on the WM updating task was not greater than the EF group’s (i.e., pre-post training gains were comparable). In contrast, the WM group did not demonstrate training-related improvements to other domains (cognitive flexibility and inhibitory control/selective attention) that were trained in the EF group.

Notably, there was only a main effect of time for the Flanker task (inhibitory control/selective attention), indicating general pre-post improvements across participants, regardless of group (despite the EF group training on a Flanker task). This may reflect practice effects, or the possibility that all training programs indirectly trained attention, with each requiring participants to focus on stimuli for extended periods of time. This is further supported by the absence of group differences in perceived improvement in attention. These findings highlight the need for further research to clarify the mechanisms underlying such general improvements—particularly whether they result from task-specific practice or broader cognitive engagement across training programs. Future studies could explore these effects by incorporating additional inhibitory control and attention measures to better distinguish between targeted training outcomes and general cognitive benefits.

##### **Near Transfer**

A surprising finding in this study was the ambiguous evidence across each outcome of the global EF task (measured using the WCST). Bayesian analyses revealed ambiguous evidence for a Time × Group interaction for accuracy and no interaction regarding perseveration errors and categories completed. However, there was decisive evidence for a main effect of time across all outcomes, indicating that participants, regardless of group, generally improved from pre- to post-test.

While practice effects offer a plausible explanation for these findings, alternative explanations should also be considered. The WCST, while assessing EFs, also heavily relies on attention processes, requiring attentional resources to maintain correct responding (Aronson & Bennett, 2024; Greve et al., 1996). If each training program indirectly trained attention through tasks requiring sustained focus, it is reasonable that all groups showed improvements over time. Given the ambiguous evidence for this task, further research is needed to investigate the potential for EF training to transfer more robustly and to determine whether engagement in generally cognitively stimulating activities might enhance attention capacity. Such improvements in attention may have broader implications for cognitive processes, including global executive functioning.

##### **Far Transfer**

The EF group demonstrated training-related transfer to prospective memory (Virtual Week (VW) task), exhibiting larger pre-post gains compared to the AC group across all VW task types (regular, irregular, time-based). In contrast, the WM group only demonstrated larger pre-post gains for regular tasks compared to the AC group. Thus, whilst both EF and WM groups demonstrated clear improvements in prospective memory for recurring, predictable tasks (e.g., take medication after breakfast each day), only the EF group demonstrated clear improvements (in comparison to the AC group) in prospective memory for one-off tasks that occur at unpredictable times (e.g., pick up pens when you are at the shops today) and memory for tasks that occur at a specific time and necessitate the monitoring of time (e.g., check blood pressure when 2 min have elapsed).

Frameworks of prospective memory have suggested that core EFs play a critical role in prospective memory performance: WM updating is required for all types of prospective memory tasks (regular, irregular, time-based) to maintain the intention in mind; cognitive flexibility is required to shift between tasks and goals (e.g., time-monitoring); inhibitory control is required to suppress irrelevant actions or distractions that could interfere with prospective intentions/actions (Kliegel et al., 2002; Zuber et al., 2020). Thus, the training of WM in both the EF and WM training groups may have improved participants’ ability to maintain and update prospective intentions and actions. While it could be expected that the additional training of inhibitory control and cognitive flexibility in the EF program might confer further advantages for irregular and time-based tasks (e.g., facilitating time-monitoring through switching or inhibiting distractions to act at the right moment), this was not observed in the current study. Instead, improvements across groups suggest that gains in prospective memory were more likely driven by WM training components common to both programs. Overall, these findings indicate that strengthening WM alone may be sufficient to support improvements in prospective memory performance, whereas broader EF training did not confer additional benefits in this context.

Consistent with previous cognitive training studies (e.g., Borella et al., 2014; Goghari & Lawlor-Savage, 2017; Kray & Feher, 2017; von Bastian et al., 2013), no training-related improvements were observed for fluid intelligence/reasoning. Despite the frequently observed association between EFs—particularly WM—and reasoning (e.g., Au et al., 2015), training EFs may not improve reasoning ability as the specific cognitive and neural mechanisms underlying EFs and reasoning may be distinct, thus limiting the generalizability of potential training effects (Diamond et al., 2016). In the context of this study, the skills being trained (task-switching, WM, and inhibitory control) may not be directly required for discerning patterns (assessed in the CFIT). 

These findings for prospective memory and fluid intelligence align with theories of cognitive transfer such as Gathercole et al.’s (2019) cognitive routine framework which posits that cognitive training induces novel and challenging cognitive demands that cannot be met by the current neurocognitive system. This system must therefore develop new ‘cognitive routines’ (procedures) that can be learnt and automated through repeated practice and subsequently be applied to untrained tasks sharing the same processes. Thus, this study’s WM training may have facilitated the development of new, more efficient cognitive routines which could be applied to Virtual Week (prospective memory) which requires the use of WM and/or WM updating (Strauss et al., 2006; Zuber et al., 2020). Further, as cognitive flexibility is critical for successful completion of prospective memory tasks (Kliegel et al., 2002), training cognitive flexibility and inhibitory control in the EF group may have developed additional cognitive routines which allowed for more efficient processing. This aligns with the more consistent improvements (relative to the AC group) for the multidomain EF group than the single-domain WM group. As neither training group demonstrated transfer to the CFIT (fluid intelligence/reasoning), this suggests that any new cognitive routines developed through training did not align with the processes required to complete the CFIT and therefore could not be applied to elicit improved performance (transfer). Instead, all groups, including the active control, showed improvement over time on the CFIT, indicating that these gains are more likely attributable to practice effects or increased familiarity with the test rather than training-related transfer.

These findings generally provide support for Hypotheses 1a and 2a, with the multidomain EF training promoting clear training and transfer effects on most cognitive outcomes (N-Back, Task switching/mixing, WCST, and Virtual Week regular, irregular and time-based tasks) relative to the AC group, and the single-domain WM training promoting improved performance on memory-related outcomes (N-Back and Virtual Week regular tasks). Further, the magnitude of pre-post gains between the EF and WM groups was generally comparable, with mostly ambiguous or substantial evidence for no difference between groups. Considering the ambiguity of many outcomes, there is not sufficient evidence to provide overall support for Hypothesis 3 that multidomain EF training would elicit larger improvements than single-domain WM training. However, cognitive flexibility may represent a notable exception, as the EF group demonstrated substantially larger training gains on task-switching (i.e., reduced switch costs and mixing costs) compared to the WM group, providing limited support for Hypothesis 3 in this domain. Notably, the EF group also tended to demonstrate larger gains across trained and transfer tasks (relative to the AC group) compared to the WM group. Thus, this study suggests that adaptive, multidomain EF training may be a promising tool for promoting cognitive transfer.

Despite common arguments for the benefits of multidomain over single-domain training (e.g., Guye et al., 2021), there is a dearth of empirical evidence backing these claims (e.g., Anguera et al., 2013; Binder et al., 2016). This study is therefore one of the first to provide clear evidence for the potential efficacy of multidomain core EF training compared to single-domain (WM) training. However, further research is needed to better understand the mechanisms driving transfer in each condition, and whether multidomain approaches yield more robust or long-lasting benefits across diverse cognitive domains.

Further, this study is one of few studies to provide evidence for training-related transfer to prospective memory, and the only study exploring this in relation to multidomain EF training. Whilst results demonstrated transfer to prospective memory after both single-domain WM and multidomain EF training, a prior training study reported no transfer. After four sessions of training on a categorization WM span task, Hering et al. (2017) reported no improvement in a laboratory prospective memory task. However, training in that study lacked adaptive difficulty, which might have limited its potential to enhance WM to the fullest extent—evident by the limited transfer to other WM tasks—and consequently limited the potential for transfer to prospective memory. Additionally, the prospective memory task used by Hering et al. (2017) required participants to remember abstract letter-color combinations which may not accurately reflect the variety of tasks that older adults must remember in their everyday lives. In contrast, the current study integrated adaptive difficulty during training to enhance potential training-related cognitive improvements, and utilized Virtual Week—a more naturalistic and comprehensive assessment of prospective memory. As this task assesses performance on different types of tasks (regular, irregular, time-based) it requires participants to make decisions and remember to perform tasks as they would in the real world (e.g., take medication at breakfast and dinner), which may also assist in the generalizability of findings. Research in this area is of particular significance as prospective memory is a key predictor of functional independence in older adults (Hering et al., 2018; Sheppard et al., 2019), with numerous cognitive training studies specifically targeting this cognitive domain—many of which have used the Virtual Week task as the training program (Rose et al., 2015; Tse et al., 2023). Based on the findings from the current study, EF training may be effective in enhancing prospective memory and may promote broader transfer than single-domain prospective memory training as the specific cognitive routines developed during prospective memory-only training may not be applicable (generalizable) to many other tasks (compared to core EFs which are thought to be the building blocks of numerous cognitive domains and everyday tasks; Diamond, 2013).

#### Comparison to young adults

Older adults’ performance across all tasks and task conditions at pre-test was worse than that of young adults. At post-test, both EF and WM groups’ performance was comparable to young adults in WM updating in the 1-back and 2-back conditions. The EF group’s performance in the most difficult 3-back condition was also comparable to young adults. For the 3-back there was ambiguous evidence for comparable performance for the WM and young adult groups. The EF group demonstrated comparable performance on mixing-costs (task-switching) but ambiguous evidence for comparable performance on the Flanker effect (inhibitory control) and switch-costs (task-switching) relative to young adults. These findings suggest that while training programs can promote improvements, the ambiguity observed in outcomes for inhibitory control and task-switching may reflect variability in participant responses to training. It may therefore be the case that longer or more intensive training could further enhance cognitive performance. Nevertheless, the general pattern of findings in the current study are consistent with that of Anguera et al. (2013) who observed that, after older adults completed a 4-week multitasking executive control video game training program, their performance (and neural activity) on this trained task was comparable to that of young adults. The current findings build on their work, emphasizing that EF training programs have the potential to narrow age-related gaps in cognition, albeit with limits depending on the domain(s) trained. However, the variability observed in the present results calls into question the strength of evidence from previous cognitive training research, which utilize traditional null hypothesis significance testing. Future studies should consider incorporating Bayesian statistics to evaluate the strength of evidence more robustly. This approach would provide a clearer understanding of the degree of improvement and consistency of training effects, offering a more in-depth evaluation of the efficacy of cognitive training.

For transfer tasks, findings regarding age differences were mixed. Whilst all groups demonstrated better WCST performance across all indices from pre- to post-test, only the EF group demonstrated comparable post-test accuracy to young adults. There was ambiguous evidence for comparable performance in EF and young adult groups on perseveration errors and categories completed. In contrast, there was only ambiguous evidence for comparable performance between the WM group and young adults on perseveration errors.

The categories completed index may be particularly challenging for older adults as it requires not only initial learning of sorting rules, but also the ability to consistently shift to new rules once reinforcement contingencies change. Thus, the training may not have been sufficient to improve the ability to maintain task-relevant representations and efficiently transition between cognitive sets. Consequently, even with training, older adults may struggle to complete as many categories as young adults due to a reduced ability to suppress prior task rules and flexibly engage with new ones. Training effects may also be more pronounced in tasks that provide relatively purer measures of working memory or inhibitory control, rather than tasks like the WCST that require the dynamic integration of multiple executive processes over an extended period. These findings highlight the potential limitations of short-term cognitive training interventions in fully remediating age-related executive function declines, particularly in tasks that require continuous adaptation and rule-switching over time.

In the Virtual Week task, all older adult groups continued to perform worse than young adults on regular and irregular tasks at post-test. Despite pre-post improvements on all Virtual Week task types in the EF and WM groups, there are generally large age-related differences in prospective memory performance (e.g., Rendell et al., 2000). Therefore a 4-week training program may not have been sufficient for restoring performance to match the level of young adults. Interestingly, all older adult groups (including the AC group) exhibited comparable post-test performance to young adults on time-based tasks. Of all prospective memory tasks, time-based tasks generally require the most cognitive resources as they do not provide cues for when tasks must be performed; rather, successful execution of these types of tasks requires one to monitor the progression of time (Zuber et al., 2020). This skill may have been indirectly trained across all training groups as participants were constantly required to monitor the time to ensure that the duration of their training session did not exceed 45 min. Additionally, the incorporation of timers in most games (counting down the remaining time) may have increased older adults’ attentiveness to temporal cues, thereby potentially improving their performance on time-based tasks in the Virtual Week measure at post-test. As all training groups only exhibited comparable performance to young adults on time-based tasks (and not regular or irregular tasks) at post-test, this likely negates the possibility of practice effects. Further research is required to corroborate these findings and provide clearer insight into age-related differences in prospective memory and the potential for training to restore performance in this domain.

Overall, these findings provide partial support for Hypotheses 1b and 2b as the post-test performance of EF and WM groups were comparable to that of untrained young adults, albeit for limited outcomes. This benchmark moves beyond showing relative improvements within older adults to asking whether training can meaningfully reduce or close age-related performance gaps. This study contributes to the literature by offering insights into the extent to which EF training might “restore” cognitive functioning to achieve performance levels comparable to young adults. Despite demonstrating pre-post improvements on transfer tasks, the strength of evidence for performance of EF and WM training groups being comparable to young adults on global executive functioning was mostly ambiguous, whereas poorer performance was maintained on most aspects of a prospective memory task. Although full restoration to young adult levels was not consistently observed, the partial convergence seen in domains such as WM updating and task-switching suggests that targeted EF training may promote resilience by elevating older adults’ functioning toward a higher normative standard. From a theoretical perspective, this supports the idea that cognitive training can, at least in part, offset age-related decline in directly trained tasks of executive functioning. From a practical perspective, it suggests that well-designed training programs might help older adults maintain independence and everyday functioning by bolstering capacities most closely tied to age-sensitive executive processes.

#### Consideration of cognitive transfer

Theories of cognitive training and transfer generally agree that cognitive transfer only occurs when both training and transfer tasks share similar general task elements (e.g., primitive elements theory; Taatgen, 2013, 2021), overlapping task demands (e.g., overlapping task demand model; Zelinski et al., 2014), or relevant cognitive routines (e.g., cognitive routines framework; Gathercole et al., 2019). This is supported by neuroimaging studies reporting the occurrence of transfer when trained and transfer tasks engage overlapping brain regions at pre-test and share the same pattern of training-related changes in these regions (e.g., Baykara et al., 2021; Dahlin et al., 2008; Heinzel et al., 2016; Salminen et al., 2016). Accordingly, given the overlapping neuroanatomy of EFs and numerous other cognitive processes required in everyday functioning, such as attention, prospective memory, and problem-solving (Goldstein et al., 2014; Nani et al., 2019; Rueda et al., 2021), cognitive training targeting EFs should promote broad cognitive transfer and be useful in remediating the effects of age-related cognitive decline (Karbach & Kray, 2021). Therefore, training multiple cognitive domains (multidomain training) should be more beneficial than single-domain training as exercising a broad range of domains would activate a more widespread neural circuitry, thereby having the potential to produce broader transfer effects (Guye et al., 2021; Könen et al., 2016).

The findings from the current study generally support this notion, with multidomain EF training exhibiting larger pre-post gains relative to an AC group across more tasks and outcomes than the WM training group. Whilst WM is implicated in both executive functioning and prospective memory (Nyhus & Barceló, 2009; Wang et al., 2013), all core EFs—particularly cognitive flexibility—are involved in efficiently executing these types of tasks (Gamboz et al., 2009; Kliegel et al., 2002; Miles et al., 2021; Zuber et al., 2020). Thus, stronger and more consistent cognitive transfer may have been promoted through multidomain training (compared to training WM alone) due to the development of new information processing elements or cognitive routines pertaining to updating, as well as switching/shifting and suppressing distracting information, which could then be applied to execute these untrained tasks more efficiently.

Notably, however, the evidence pertaining to training-related changes (interactions) for some outcomes and many pre-post gain comparisons between the EF and WM groups (post-hoc tests) were ambiguous. One factor that may help explain this pattern is the difference in training dosage across conditions. In the present study, the WM training group completed three 45-min WM sessions per week for four weeks, whereas the EF training group completed one 45-min session per week for each EF domain (WM, cognitive flexibility, inhibitory control). Thus, the multidomain program provided less practice time on each individual EF relative to the single-domain condition. However, each weekly session was relatively lengthy compared to other training programs, and the games themselves (~ 3 min each) required sustained engagement, which may be important given that cognitive plasticity is thought to be a relatively “sluggish” process that benefits from prolonged stimulation (Lövdén et al., 2010). These dosage differences are consistent with our findings, such that, the WM training group showed slightly (though not substantially) larger gains on the WM updating transfer task, while the EF training group exhibited broader transfer across more outcomes relative to the AC group. This pattern suggests that intensive, repeated practice on a single domain may confer modest advantages for that domain, whereas distributing practice across multiple EFs can foster wider transfer by engaging diverse executive processes. It is therefore possible that with a longer training period, the multidomain EF condition could have shown even clearer evidence of transfer to global EF and prospective memory tasks. Further research is needed to systematically examine dosage effects in single- versus multidomain training to clarify how intensity and breadth of practice interact to produce transfer.

Another consideration is that, whilst cognitive training has the potential to improve cognitive and neural efficiency, sufficient time is needed for these improvements to manifest through behavioural indices (e.g., reaction time), which may potentially contribute to some of the mixed findings within this study and the extant literature. Furthermore, behavioural indices of cognitive functioning may be more subtle due to other aging-related factors, such as lower mobility. Thus, some of the mixed findings within this study could potentially be attributed to a combination of delayed behavioural manifestation of training effects and more subtle behavioural changes. The integration of neuroimaging methods is critical for advancing the field of cognitive training by providing a more comprehensive and clearer insight into the mechanisms underlying cognitive transfer.

### Subjective outcomes

Perceived attention, memory, and speed/reaction time skills increased from pre- to post-test for both EF and WM groups, with these improved ratings being maintained at the 1-month follow-up. The EF and WM groups reported pre-post decreases in perceived deficits in attention and retrospective memory, whereas only the WM group reported a reduction in perceived prospective memory deficits. All decreases in perceived deficit scores were maintained at follow-up. These findings align with Goghari and Lawlor-Savage (2018) who also found that, after training, participants reported fewer cognitive failures relevant to daily life (compared a passive control group). Further, there was a general reduction in perceived deficits in organization/planning skills across time. This may be an indirect consequence of the training regimen itself, as participants were required to organize and plan their weeks to schedule in the training which may have enhanced their practical organization and planning skills, reflecting an unintended benefit of engaging in a cognitive training program.

All groups in the current study also reported increased subjective well-being scores from pre- to post-test, which were maintained at the 1-month follow-up. This is similar to previous studies reporting improvements in well-being indices across both training and control groups (Ballesteros et al., 2014). This finding could be attributed to general participation in a new or different activity, which can foster a sense of purpose, stimulate social interaction and mental engagement, and promote a sense of accomplishment, thereby enhancing well-being (Sutin et al., 2021). No changes in depressive symptoms were observed for any group in the current study, which is consistent with previous research (e.g., Shah et al., 2014). This may be due to participants within this study reporting few depressive symptoms initially and therefore had little room for change. This may account for the difference compared to previous studies. For example, in a study involving adults experiencing clinical depression, it was reported that cognitive training may alleviate depressive symptoms by stimulating activity/functioning in neural regions implicated in mood regulation (e.g., Motter et al., 2016). Further, evidence from the Advanced Cognitive Training for Independent and Vital Elderly (ACTIVE) study reported that cognitive training (specifically, speed of processing training) reduced the risk of depressive symptoms at a 1- and 5-year follow-up (Wolinsky et al., 2009).

These findings partially support Hypothesis 1a as the multidomain EF training group demonstrated improved performance on many (but not all) subjective outcomes compared to the AC group. Hypothesis 2a was supported as the WM group demonstrated larger memory-related subjective cognitive outcomes compared to the AC group. Hypothesis 3 was not supported for the subjective findings as there was no difference in the magnitude of training-related gains on these subjective outcomes between the EF and WM groups.

Given that subjective outcomes are understudied in the cognitive training literature, these findings are important as older adults’ perceptions of aging, age-related changes, and beliefs about their own cognitive functioning have implications for their actual cognitive functioning, physical health, health-related behaviors, and even longevity (Goghari & Lawlor-Savage, 2018; Levy et al., 2012; Westerhof et al., 2023). Numerous large-scale studies have reported links between subjective and objective cognition. For instance, Freed et al. (2024) observed a stable correlation between objective and subjective memory in a sample of healthy older adults (*N* = 2,496). Further, across a large sample of cognitively healthy older adults (*N* = 6,056), Sabatini et al. (2021) reported that awareness of negative age-related change was associated with poorer cognition, higher levels of depression and anxiety, more negative self-perceptions of aging, and poorer self-rated health. Improving self-appraisals of one’s own capabilities may also promote a ‘cascading effect’ where training-related enhancements in subjective perceptions of cognitive abilities and self-efficacy may positively impact objective performance on other tasks and/or situations (Jaeggi & Buschkuehl, 2014).

The current study therefore contributes to the literature by exploring how cognitive training may impact upon individuals’ perceptions of their cognitive abilities and well-being. In particular, these findings suggest that cognitive training has the potential to elevate older adults’ perceptions of their cognitive functioning. Additionally, engaging in a new or different activity such as cognitive training may positively influence older adults’ well-being, regardless of the type of training. Further research is warranted to explore other subjective outcomes (e.g., awareness of age-related changes, perceptions of aging, affect, sense of purpose) to discover the full potential of cognitive training.

### Strengths, Limitations, and future directions

A key strength of this study was the application of Bayesian analyses, allowing for the quantification of the strength of evidence pertaining to cognitive training-related changes (Goghari & Lawlor-Savage, 2018; Harrell et al., 2023; Rabipour et al., 2019; Schmiedek, 2021). This yielded a stronger understanding of the degree and consistency of training-related improvements, identifying both promising outcomes and areas of ambiguity that warrant further investigation. Interestingly, had the results been interpreted solely through a Frequentist lens, the findings would have largely supported expected cognitive training and transfer effects, aligning with existing cognitive transfer frameworks and previous research. For instance, frequentist analyses indicated no statistically significant differences between the EF training group and young adults on all outcomes except VW (regular and irregular tasks) and similarly suggested no significant differences between the WM group and young adults on WCST accuracy and perseveration errors (see Supplementary Materials). However, Bayesian analyses revealed greater ambiguity, with several findings—including those related to trained tasks such as inhibitory control—showing inconclusive evidence rather than strong support for either the null or alternative hypothesis. This divergence highlights the limitations of relying solely on frequentist analyses, which does not quantify evidence for the null hypothesis and may lead to overinterpretation of non-significant results. Moving forward, it is critical for cognitive training research to incorporate Bayesian analyses to more rigorously assess the strength of evidence for or against training effects, thereby improving the reliability and interpretability of findings in this field.

A caveat of the current study, however, was that the number of cognitive outcomes—particularly transfer tasks—was relatively limited and could have been investigated across more domains to further explore to which other cognitive domains multidomain training and single-domain training might transfer. Additionally, including multiple measures of a cognitive domain (e.g., two different prospective memory tasks) would help determine whether training elicited improvements on a task-specific skill or the actual underlying ability (Schmiedek, 2021). Critically, whilst large test batteries can be beneficial for evaluating the extent of transfer, assessing cognitive performance on numerous tasks may introduce problems that may paradoxically interfere with the inference of transfer. For instance, larger test batteries may increase participant burden and motivational and/or cognitive fatigue, introduce carry-over effects, and increase the potential for Type I errors (e.g., Green et al., 2019). As this study also incorporated numerous subjective measures, a smaller set of cognitive outcome measures was included to minimize participant fatigue. It is important to strike a balance between including a sufficient number of outcome measures to provide a comprehensive evaluation of transfer whilst avoiding the inclusion of too many measures which may “contaminate” the investigation by introducing error and bias. Thus, future studies may choose to identify key domains which might be of interest to evaluate the extent of transfer, and include two measures for each domain to confirm whether any potential improvements were task-specific or may have transferred to the underlying ability.

Additionally, the 1-month follow-up for subjective outcomes and no follow-up for cognitive outcomes (designed as such due to feasibility) presents challenges in definitively addressing the maintenance of training-related changes. However, cognitive training should not be treated as a quick-fix, one-shot treatment; rather, sustained engagement would be more beneficial to promote long-term and potentially larger gains in cognition and well-being (e.g., Guye & Von Bastian, 2017; Nguyen et al., 2021). Going forward, research could therefore focus attention on elements that might influence enjoyment and motivation for continued engagement in cognitive training.

These limitations notwithstanding, the study’s findings are of significance as the potential to enhance executive functioning has important implications for functional independence in older adulthood, with core EFs forming the foundations for nearly all aspects of life (e.g., mental health, physical health, quality of life, everyday activities; Diamond, 2013). Similarly, the potential to elevate older adults’ perceptions of their own capabilities has clinical importance for actual capabilities, mental health, and well-being (Sabatini et al., 2021; Westerhof et al., 2023). Considering the lack of studies comparing the efficacy of multidomain versus single-domain core EF training, and limited research offering insights into the potential training-related benefits to subjective outcomes, the findings of the current study sheds light on the effectiveness and broader implications of cognitive training interventions for older adults.

### Conclusion

The current study provided empirical evidence for the direct comparison between multidomain and single-domain core EF training in older adults, revealing some support for broader transfer effects after multidomain compared to single-domain training. After multidomain EF training, the performance of older adults on many executive functioning outcomes was comparable to that of young adults (though there was ambiguous evidence for comparable performance on some outcomes). However, performance on far-transfer tasks (prospective memory) was still poorer than young adults, warranting further investigation regarding dosage (i.e., how much training might be needed to improve performance on far-transfer tasks to the level of young adults). Both EF and WM training elevated older adults’ perceptions of many aspects of their own cognitive functioning, which may promote confidence, self-efficacy, and more positive self-perceptions of aging. Given improvements in well-being were evident for all groups, it may be the case that engagement in novel tasks or general cognitive stimulation could contribute to improved quality of life. Whilst there is still much work to be done in the field of cognitive enhancement, research into multidomain core EF training is clearly a worthwhile endeavor to facilitate the remediation of age-related cognitive decline and foster functional independence in older age.

## Supplementary Information

Below is the link to the electronic supplementary material.

Supplementary Material 1

## Data Availability

The datasets generated and analyzed in the current study are available to access in the Open Science Framework repository. This work was pre-registered on the Open Science Framework registry prior to data collection and updated prior to data analysis. Pre-registration details and data can be accessed via https://doi.org/10.17605/OSF.IO/PZJAQ.

## Footnotes

1 We distinguish multidomain cognitive training (training multiple cognitive domains) from multimodal training (training involving multiple modes or components, such as cognitive training and physical exercise and/or brain stimulation, etc.).

2 Aligns with a priori G*Power analysis (Faul et al., 2007) using a small-to-moderate effect size (η_p_^2^_=_ 0.03, *f* = 0.17), α = 0.05, power = 0.80, for 3 groups, 2 measurement occasions, *r* = .60 for repeated measures, and non-sphericity correct = 1. These parameters are similar to those described by Redick et al. (2019). It should be noted that this sample size calculation pertains to the frequentist framework and does not directly apply to Bayesian analyses.

3 Double-blinding (blinding of outcome assessor) was not possible as the study was not funded by a research grant but rather formed part of a doctoral dissertation in which the candidate was required to collect and analyze data independently.

4 Whilst it is difficult to eliminate the use of core EFs from a task or game, the games chosen did not heavily rely or focus on the domains being trained in the EF training program. The games chosen for the active control program were discussed and agreed upon by the research team.

5 1-month follow-up was only completed for subjective outcomes but not cognitive outcomes to reduce participant burden and for feasibility (prioritizing resource allocation and adhering to timeline constraints, focusing on the immediate effects of the intervention, while still capturing longer-term subjective outcomes through an online questionnaire).

6 Null hypothesis significance testing was also conducted (statistics for these analyses are reported in online Supplementary Materials).

## References

[CR1] Anguera, J. A., Boccanfuso, J., Rintoul, J. L., Al-Hashimi, O., Faraji, F., Janowich, J., Kong, E., Larraburo, Y., Rolle, C., Johnston, E., & Gazzaley, A. (2013). Video game training enhances cognitive control in older adults. *Nature*, *501*, 97–101. 10.1038/nature12486 24005416 10.1038/nature12486 PMC3983066

[CR2] Aronson, R., & Bennett, R. (2024). Wisconsin card sorting task (WCST). In C. J. Golden, & R. Bennett (Eds.), *Clinical integration of neuropsychological test results* (pp. 142–148). Taylor & Francis Group. 10.1201/9781003309604

[CR3] Au, J., Sheehan, E., Tsai, N., Duncan, G. J., Buschkuehl, M., & Jaeggi, S. M. (2015). Improving fluid intelligence with training on working memory: A meta-analysis. *Psychonomic Bulletin & Review*, *22*, 366–377. 10.3758/s13423-014-0699-x 25102926 10.3758/s13423-014-0699-x

[CR5] Ballesteros, S., Prieto, A., Mayas, J., Toril, P., Pita, C., Ponce de León, L., Reales, J. M., & Waterworth, J. (2014). Brain training with non-action video games enhances aspects of cognition in older adults: A randomized controlled trial. *Frontiers in Aging Neuroscience*, *6*, 1–14. 10.3389/fnagi.2014.00277 25352805 10.3389/fnagi.2014.00277 PMC4196565

[CR4] Baykara, E., Könen, T., Unger, K., & Karbach, J. (2021). MRI predictors of cognitive training outcomes. *Journal of Cognitive Enhancement*, *5*, 245–258. 10.1007/s41465-020-00188-y

[CR6] Binder, J. C., Martin, M., Zollig, J., Rocke, C., Merillat, S., Eschen, A., Jancke, L., & Shing, Y. L. (2016). Multi-domain training enhances attentional control. *Psychology and Aging*, *31*, 390–408. 10.1037/pag0000081 27294719 10.1037/pag0000081

[CR7] Boot, W. R., Champion, M., Blakely, D. P., Wright, T., Souders, D. J., & Charness, N. (2013). Video games as a means to reduce age-related cognitive decline: Attitudes, compliance, and effectiveness. *Frontiers in Psychology*, *4*, 31–40. 10.3389/fpsyg.2013.00031 23378841 10.3389/fpsyg.2013.00031 PMC3561600

[CR8] Boot, W. R., Souders, D. J., Charness, N., Blocker, K., Roque, N., & Vitale, T. (2016). The gamification of cognitive training: Older adults’ perceptions of and attitudes toward digital game-based interventions. *International Conference on Human Aspects of IT for the Aged Population*, *9754*, 290–300. 10.1007/978-3-319-39943-0_28

[CR9] Borella, E., Carretti, B., Cantarella, A., Riboldi, F., Zavagnin, M., & De Beni, R. (2014). Benefits of training visuospatial working memory in young-old and old-old. *Developmental Psychology*, *50*, 714–727. 10.1037/a0034293 24059254 10.1037/a0034293

[CR10] Bures, V., Cech, P., Mikulecka, J., Ponce, D., & Kuca, K. (2016). The effect of cognitive training on the subjective perception of well-being in older adults. *PeerJ*, *4*, e2785. 10.7717/peerj.2785 28028465 10.7717/peerj.2785 PMC5180580

[CR11] Cambridge Cognition (n.d.). *Intra-Extra Dimensional Set Shift (IED)*. Cambridge Cognition. Retrieved 23 July from https://cambridgecognition.com/intra-extra-dimensional-set-shift-ied

[CR12] Cattell, R. B., & Cattell, A. K. S. (1960). *Handbook for the individual or group culture fair intelligence test*.

[CR13] Cochrane, A., & Green, C. S. (2021). New directions in training designs. In T. Strobach, & J. Karbach (Eds.), *Cognitive training: An overview of features and applications* (2nd ed., pp. 25–40). Springer.

[CR15] Cosentino, S., Devanand, D., & Gurland, B. (2018). A link between subjective perceptions of memory and physical function: Implications for subjective cognitive decline. *Journal of Alzheimer’s Disease*, *61*(4), 1387–1398. 10.3233/JAD-170495 29376850 10.3233/JAD-170495 PMC6436538

[CR16] Dahlin, E., Stigsdotter Neely, A., Larsson, A., Bäckman, L., & Nyberg, L. (2008). Transfer of learning after updating training mediated by the striatum. *Science*, *320*, 1510–1512. 10.1126/science.1155466 18556560 10.1126/science.1155466

[CR17] Das, P., & Steyvers, M. (2023). Older adults catch up to younger adults on cognitive tasks after extended training. *PsyArXiv*. https://psyarxiv.com/6jd3c/download?format=pdf

[CR18] De Simoni, C., & von Bastian, C. C. (2018). Working memory updating and binding training: Bayesian evidence supporting the absence of transfer. *Journal of Experimental Psychology: General*, *147*(6), 829–858. 10.1037/xge0000453 29888939 10.1037/xge0000453

[CR19] Denkinger, S., Spano, L., Bingel, U., Witt, C. M., Bavelier, D., & Green, C. S. (2021). Assessing the impact of expectations in cognitive training and beyond. *Journal of Cognitive Enhancement*, 1–17. 10.1007/s41465-021-00206-7

[CR20] Diamond, A. (2013). Executive functions. *Annual Review of Psychology*, *64*, 135–168. 10.1146/annurev-psych-113011-143750 23020641 10.1146/annurev-psych-113011-143750 PMC4084861

[CR21] Diamond, A., & Ling, D. S. (2016). Conclusions about interventions, programs, and approaches for improving executive functions that appear justified and those that, despite much hype, do not. *Developmental Cognitive Neuroscience*, *18*, 34–48. 10.1016/j.dcn.2015.11.005 26749076 10.1016/j.dcn.2015.11.005 PMC5108631

[CR22] Dorbath, L., Hasselhorn, M., & Titz, C. (2013). Effects of education on executive functioning and its trainability. *Educational Gerontology*, *39*, 314–325. 10.1080/03601277.2012.700820

[CR23] Dougherty, M. R., Hamovitz, T., & Tidwell, J. W. (2016). Reevaluating the effectiveness of n-back training on transfer through the bayesian lens: Support for the null [journal article]. *Psychonomic Bulletin & Review*, *23*, 306–316. 10.3758/s13423-015-0865-9 26082280 10.3758/s13423-015-0865-9

[CR25] Faul, F., Erdfelder, E., Lang, A., & Buchner, A. (2007). G*Power 3: A flexible statistical power analysis program for the social, behavioral, and biomedical sciences. *Behavior Research Methods*, *39*, 175–191. 10.3758/BF03193146 17695343 10.3758/bf03193146

[CR26] Folstein, M. F., Folstein, S. E., & McHugh, P. R. (1975). Mini-mental state: A practical method for grading the cognitive state of patients for the clinician. *Journal of Psychiatric Research*, *12*, 189–198. 10.1016/0022-3956(75)90026-6 1202204 10.1016/0022-3956(75)90026-6

[CR27] Freed, S. A., Sprague, B. N., & Ross, L. A. (2024). Does the association between objective and subjective memory vary by age among healthy older adults? *Aging Neuropsychology and Cognition*, *31*(2), 249–262. 10.1080/13825585.2022.2143471 10.1080/13825585.2022.2143471 PMC10166764 36353743

[CR28] Friedman, B., Heisel, M. J., & Delavan, R. L. (2005). Psychometric properties of the 15-item geriatric depression scale in functionally impaired, cognitively intact, community‐dwelling elderly primary care patients. *Journal of the American Geriatrics Society*, *53*(9), 1570–1576. 10.1111/j.1532-5415.2005.53461.x 16137289 10.1111/j.1532-5415.2005.53461.x

[CR29] Gajewski, P. D., & Falkenstein, M. (2012). Training-induced improvement of response selection and error detection in aging assessed by task switching: Effects of cognitive, physical, and relaxation training. *Frontiers in Human Neuroscience*, *6*, 130–148. 10.3389/fnhum.2012.00130 22593740 10.3389/fnhum.2012.00130 PMC3349932

[CR30] Galvin, J. E., Tolea, M. I., Rosenfeld, A., & Chrisphonte, S. (2020). The quick physical activity rating (QPAR) scale: A brief assessment of physical activity in older adults with and without cognitive impairment. *PloS One*, *15*(10), e0241641. 10.1371/journal.pone.0241641 33125429 10.1371/journal.pone.0241641 PMC7598491

[CR31] Gamboz, N., Borella, E., & Brandimonte, M. A. (2009). The role of switching, Inhibition and working memory in older adults’ performance in the Wisconsin card sorting test. *Aging Neuropsychology and Cognition*, *16*(3), 260–284. 10.1080/13825580802573045 10.1080/13825580802573045 19105052

[CR32] Gathercole, S. E., Dunning, D. L., Holmes, J., & Norris, D. (2019). Working memory training involves learning new skills. *Journal of Memory and Language*, *105*, 19–42. 10.1016/j.jml.2018.10.003 10.1016/j.jml.2018.10.003 PMC6591133 31235992

[CR33] Gobet, F., & Sala, G. (2023). Cognitive training: A field in search of a phenomenon. *Perspectives on Psychological Science*, *18*(1), 125–141. 10.1177/17456916221091830 35939827 10.1177/17456916221091830 PMC9903001

[CR34] Goghari, V. M., & Lawlor-Savage, L. (2017). Comparison of cognitive change after working memory training and logic and planning training in healthy older adults. *Frontiers in Aging Neuroscience*, *9*, 39–51. 10.3389/fnagi.2017.00039 28293187 10.3389/fnagi.2017.00039 PMC5328972

[CR35] Goghari, V. M., & Lawlor-Savage, L. (2018). Self-perceived benefits of cognitive training in healthy older adults. *Frontiers in Aging Neuroscience*, *10*, 112. 10.3389/fnagi.2018.00112 29922146 10.3389/fnagi.2018.00112 PMC5996899

[CR36] Goghari, V. M., Krzyzanowski, D., Yoon, S., Dai, Y., & Toews, D. (2020). Attitudes and beliefs toward computerized cognitive training in the general population. *Frontiers in Psychology*, *11*(503), 1–11. 10.3389/fpsyg.2020.00503 32318000 10.3389/fpsyg.2020.00503 PMC7147517

[CR37] Goldstein, S., & Naglieri, J. A. (2014). *Handbook of executive functioning*. Springer.

[CR38] Goldstein, S., Naglieri, J. A., Princiotta, D., & Otero, T. M. (2014). Introduction: A history of executive functioning as a theoretical and clinical construct. In S. Goldstein, & J. A. Naglieri (Eds.), *Handbook of executive functioning* (pp. 3–12). Springer.

[CR39] Goss-Sampson, M. A., van Doorn, J., & Wagenmakers, E. J. (2020). *Bayesian inference in JASP: A guide for students (v0.12.2)*. JASP Team. https://jasp-stats.org/jasp-materials/

[CR40] Green, C. S., Bavelier, D., Kramer, A. F., Vinogradov, S., Ansorge, U., Ball, K. K., Bingel, U., Chein, J. M., Colzato, L. S., Edwards, J. D., Facoetti, A., Gazzaley, A., Gathercole, S. E., Ghisletta, P., Gori, S., Granic, I., Hillman, C. H., Hommel, B., Jaeggi, S. M., & Witt, C. M. (2019). Improving methodological standards in behavioral interventions for cognitive enhancement. *Journal of Cognitive Enhancement*, 1–28. 10.1007/s41465-018-0115-y

[CR41] Guye, S., & Von Bastian, C. C. (2017). Working memory training in older adults: Bayesian evidence supporting the absence of transfer. *Psychology and Aging*, *32*(8), 732–746. 10.1037/pag0000206 29239658 10.1037/pag0000206

[CR42] Guye, S., Röcke, C., Mérillat, S., von Bastian, C. C., & Martin, M. (2021). Cognitive training across the adult lifespan. In T. Strobach, & J. Karbach (Eds.), *Cognitive training: An overview of features and applications* (2nd ed., pp. 141–153). Springer.

[CR43] Harrell, E. R., Roque, N. A., & Boot, W. R. (2023). Comparing the effectiveness of two theory-based strategies to promote cognitive training adherence. *Journal of Experimental Psychology: Applied*. 10.1037/xap0000485 37471032 10.1037/xap0000485

[CR44] Harvey, P. D., McGurk, S. R., Mahncke, H. W., & Wykes, T. (2018). Controversies in computerized cognitive training. *Biological Psychiatry: Cognitive Neuroscience and Neuroimaging*, *3*, 907–915. 10.1016/j.bpsc.2018.06.008 30197048 10.1016/j.bpsc.2018.06.008

[CR45] Heinzel, S., Lorenz, R. C., Pelz, P., Heinz, A., Walter, H., Kathmann, N., Rapp, M. A., & Stelzel, C. (2016). Neural correlates of training and transfer effects in working memory in older adults. *Neuroimage*, *134*, 236–249. 10.1016/j.neuroimage.2016.03.068 27046110 10.1016/j.neuroimage.2016.03.068

[CR46] Henneghan, A., Stuifbergen, A., Becker, H., Kullberg, V., & Gloris, N. (2017). Perceived cognitive deficits in a sample of persons living with multiple sclerosis. *The Journal of Neuroscience Nursing*, *49*(5), 274. 10.1097/2FJNN.0000000000000314 28885466 10.1097/JNN.0000000000000314 PMC5657431

[CR48] Hering, A., Meuleman, B., Bürki, C., Borella, E., & Kliegel, M. (2017). Improving older adults’ working memory: The influence of age and crystallized intelligence on training outcomes. *Journal of Cognitive Enhancement*, *1*(4), 358–373. 10.1007/s41465-017-0041-4

[CR47] Hering, A., Kliegel, M., Rendell, P. G., Craik, F. I. M., & Rose, N. S. (2018). Prospective memory is a key predictor of functional independence in older adults. *Journal of the International Neuropsychological Society*, *24*, 1–6. 10.1017/S1355617718000152 29606153 10.1017/S1355617718000152

[CR49] Hyde, M., Wiggins, R. D., Higgs, P., & Blane, D. B. (2003). A measure of quality of life in early old age: The theory, development and properties of a needs satisfaction model (CASP-19). *Aging & Mental Health*, *7*(3), 186–194. 10.1080/1360786031000101157 12775399 10.1080/1360786031000101157

[CR50] IBM Corp (2023). *IBM SPSS Statistics for Windows* (Version 29.0) [Computer Software]. IBM Corp. https://www.ibm.com/spss

[CR51] Ishihara, S. (1918). Tests for color blindness. *American Journal of Ophthalmology*, *1*(5), 376.

[CR52] Jaeggi, S. M., & Buschkuehl, M. (2014). Working memory training and transfer: Theoretical and practical considerations. In B. Toni (Ed.), *New frontiers of multidisciplinary research in STEAM-H (Science, Technology, Engineering, Agriculture, Mathematics, and Health)* (pp. 19–43). Springer International Publishing.

[CR53] JASP Team (2023). *JASP* (Version 0.18.1.0) [Computer Software]. https://jasp-stats.org/

[CR55] Karbach, J., & Kray, J. (2009). How useful is executive control training? Age differences in near and Far transfer of task-switching training. *Developmental Science*, *12*, 978–990. 10.1111/j.1467-7687.2009.00846.x 19840052 10.1111/j.1467-7687.2009.00846.x

[CR56] Karbach, J., & Kray, J. (2021). Executive function training. In T. Strobach, & J. Karbach (Eds.), *Cognitive training: An overview of features and applications* (2nd ed., pp. 199–213). Springer.

[CR58] Katz, B., Jaeggi, S. M., Buschkuehl, M., Shah, P., & Jonides, J. (2018). The effect of monetary compensation on cognitive training outcomes. *Learning and Motivation*, *63*, 77–90. 10.1016/j.lmot.2017.12.002

[CR60] Kim, G. R., Netuveli, G., Blane, D. B., Peasey, A., Malyutina, S., Simonova, G., Kubinova, R., Pajak, A., Croezen, S., & Bobak, M. (2015). Psychometric properties and confirmatory factor analysis of the CASP-19, a measure of quality of life in early old age: The HAPIEE study. *Aging & Mental Health*, *19*(7), 595–609. 10.1080/13607863.2014.938605 25059754 10.1080/13607863.2014.938605 PMC4396435

[CR61] King, M. L. (2019). The neural correlates of well-being: A systematic review of the human neuroimaging and neuropsychological literature. *Cognitive Affective & Behavioral Neuroscience*, *19*(4), 779–796. 10.3758/s13415-019-00720-4 10.3758/s13415-019-00720-4 PMC6713599 31062291

[CR63] Kliegel, M., Martin, M., McDaniel, M. A., & Einstein, G. O. (2002). Complex prospective memory and executive control of working memory: A process model. *Psychological Test and Assessment Modeling*, *44*(2), 303–318.

[CR64] Klimova, B. (2016). Computer-based cognitive training in aging. *Frontiers in Aging Neuroscience*, *8*, 313–319. 10.3389/fnagi.2016.00313 28066236 10.3389/fnagi.2016.00313 PMC5168996

[CR65] Könen, T., Strobach, T., & Karbach, J. (2016). Working memory. In T. Strobach, & J. Karbach (Eds.), *Cognitive training: An overview of features And applications* (pp. 59–68). Springer.

[CR66] Könen, T., Strobach, T., & Karbach, J. (2021). Working memory training. In T. Strobach, & J. Karbach (Eds.), *Cognitive training: An overview of features and applications* (2nd ed., pp. 155–168). Springer.

[CR67] Kray, J., & Feher, B. (2017). Age differences in the transfer and maintenance of practice-induced improvements in task switching: The impact of working-memory and Inhibition demands. *Frontiers in Psychology*, *8*, 410–428. 10.3389/fpsyg.2017.00410 28367135 10.3389/fpsyg.2017.00410 PMC5355431

[CR68] Kray, J., Dörrenbächer, S., & Campus, A. (2019). The effectiveness of training in task switching. In J. M. Novick, M. F. Bunting, M. R. Dougherty, & R. W. Engle (Eds.), *Cognitive and working memory training: Perspectives from Psychology, Neuroscience, and human development* (pp. 508–538). Oxford University Press.

[CR69] Lampit, A., Hallock, H., & Valenzuela, M. (2014). Computerized cognitive training in cognitively healthy older adults: A systematic review and meta-analysis of effect modifiers. *PLoS Medicine*, *11*, e1001756. 10.1371/journal.pmed.1001756 25405755 10.1371/journal.pmed.1001756 PMC4236015

[CR70] Levy, B. R., Zonderman, A. B., Slade, M. D., & Ferrucci, L. (2012). Memory shaped by age stereotypes over time. *Journals of Gerontology: Series B*, *67*, 432–436. 10.1093/geronb/gbr120 10.1093/geronb/gbr120 PMC3391075 22056832

[CR138] Lövdén,M., Bäckman, L., Lindenberger, U., Schaefer, S., & Schmiedek, F. (2010). A theoretical framework for the study of adult cognitive plasticity. *Psychological Bulletin, 136*(4), 659–676. 10.1037/a0020080 10.1037/a0020080 20565172

[CR71] Maraver, M. J., Gómez-Ariza, C. J., Borella, E., & Bajo, M. T. (2022). Baseline capacities and motivation in executive control training of healthy older adults. *Aging & Mental Health*, *26*(3), 595–603. 10.1080/13607863.2020.1858755 33325260 10.1080/13607863.2020.1858755

[CR72] Mason, A., Lee, R., & NTA Network. (2022). Six ways population change will affect the global economy. *Population and Development Review*, *48*(1), 51–73. 10.1111/padr.12469

[CR73] McCoy, C. E. (2017). Understanding the intention-to-treat principle in randomized controlled trials. *Western Journal of Emergency Medicine*, *18*(6), 1075–1708. 10.5811/2Fwestjem.2017.8.35985 29085540 10.5811/westjem.2017.8.35985 PMC5654877

[CR74] Melby-Lervåg, M., Redick, T. S., & Hulme, C. (2016). Working memory training does not improve performance on measures of intelligence or other measures of Far transfer: Evidence from a meta-analytic review. *Perspectives on Psychological Science*, *11*, 512–534. 10.1177/1745691616635612 27474138 10.1177/1745691616635612 PMC4968033

[CR75] Meule, A. (2017). Reporting and interpreting working memory performance in n-back tasks. *Frontiers in Psychology*, *8*, 352. 10.3389/fpsyg.2017.00352 28326058 10.3389/fpsyg.2017.00352 PMC5339218

[CR76] Miles, S., Howlett, C. A., Berryman, C., Nedeljkovic, M., Moseley, G. L., & Phillipou, A. (2021). Considerations for using the Wisconsin card sorting test to assess cognitive flexibility. *Behavior Research Methods*, *53*(5), 2083–2091. 10.3758/s13428-021-01551-3 33754321 10.3758/s13428-021-01551-3

[CR77] Mishra, J., Anguera, J. A., Ziegler, D. A., & Gazzaley, A. (2013). A cognitive framework for Understanding and improving interference resolution in the brain. *Progress in Brain Research*, *207*, 351–377. 10.1016/B978-0-444-63327-9.00013-8 24309262 10.1016/B978-0-444-63327-9.00013-8 PMC4067257

[CR78] Miyake, A., Friedman, N. P., Emerson, M. J., Witzki, A. H., Howerter, A., & Wager, T. D. (2000). The unity and diversity of executive functions and their contributions to complex frontal lobe tasks: A latent variable analysis. *Cognitive Psychology*, *41*, 49–100. 10.1006/cogp.1999.0734 10945922 10.1006/cogp.1999.0734

[CR80] Motter, J. N., Pimontel, M. A., Rindskopf, D., Devanand, D. P., Doraiswamy, P. M., & Sneed, J. R. (2016). Computerized cognitive training and functional recovery in major depressive disorder: A meta-analysis. *Journal of Affective Disorders*, *189*, 184–191. 10.1016/j.jad.2015.09.022 26437233 10.1016/j.jad.2015.09.022

[CR81] Nani, A., Manuello, J., Mancuso, L., Liloia, D., Costa, T., & Cauda, F. (2019). The neural correlates of consciousness and attention: Two sister processes of the brain. *Frontiers in Neuroscience*, *13*, 487285. 10.3389/fnins.2019.01169 10.3389/fnins.2019.01169 PMC6842945 31749675

[CR82] Ng, N. F., Schafer, R. J., Simone, C. M., & Osman, A. M. (2020). Perceptions of brain training: Public expectations of cognitive benefits from popular activities. *Frontiers in Human Neuroscience*, *14*, 1–12. 10.3389/fnhum.2020.00015 32256323 10.3389/fnhum.2020.00015 PMC7092697

[CR83] Nguyen, L., Murphy, K., & Andrews, G. (2019). Immediate and long-term efficacy of executive functions cognitive training in older adults: A systematic review and meta-analysis. *Psychological Bulletin*, *145*, 698–733. 10.1037/bul0000196 30998045 10.1037/bul0000196

[CR84] Nguyen, L., Murphy, K., & Andrews, G. (2021). A game a day keeps cognitive decline away? A systematic review and meta-analysis of commercially-available brain training programs in healthy and cognitively impaired older adults. *Neuropsychology Review*, 1–30. 10.1007/s11065-021-09515-2 10.1007/s11065-021-09515-2 34251578

[CR85] Nguyen, L., Murphy, K., & Andrews, G. (2025). Design and development of a gamified cognitive training program targeting executive functions for older adults. *Entertainment Computing*, *52*, 100892. 10.1016/j.entcom.2024.100892

[CR86] Noack, H., Lövdén, M., Schmiedek, F., & Lindenberger, U. (2009). Cognitive plasticity in adulthood and old age: Gauging the generality of cognitive intervention effects. *Restorative Neurology and Neuroscience*, *27*, 435–453. 10.3233/RNN-2009-0496 19847069 10.3233/RNN-2009-0496

[CR87] Nouchi, R., Saito, T., Nouchi, H., & Kawashima, R. (2016). Small acute benefits of 4 weeks processing speed training games on processing speed and Inhibition performance and depressive mood in the healthy elderly people: Evidence from a randomized control trial. *Frontiers in Aging Neuroscience*, *8*, 302–314. 10.3389/fnagi.2016.00302 28066229 10.3389/fnagi.2016.00302 PMC5179514

[CR88] Nyberg, L., & Eriksson, J. (2016). Working memory: Maintenance, updating, and the realization of intentions. *Cold Spring Harbor Perspectives in Biology*, *8*(2), a021816. 10.1101/cshperspect.a021816 10.1101/cshperspect.a021816 PMC4743080 26637287

[CR89] Nyhus, E., & Barceló, F. (2009). The Wisconsin card sorting test and the cognitive assessment of prefrontal executive functions: A critical update. *Brain and Cognition*, *71*(3), 437–451. 10.1016/j.bandc.2009.03.005 19375839 10.1016/j.bandc.2009.03.005

[CR90] Park, M. H., Kwon, D. Y., Seo, W. K., Lim, K. S., & Song, M. S. (2009). The effects of cognitive training on community-dwelling elderly Koreans. *Journal of Psychiatric and Mental Health Nursing*, *16*, 904–909. 10.1111/j.1365-2850.2009.01467.x 19930364 10.1111/j.1365-2850.2009.01467.x

[CR91] Phan, M. H., Keebler, J. R., & Chaparro, B. S. (2016). The development and validation of the game user experience satisfaction scale (GUESS). *Human Factors*, *58*, 1217–1247. 10.1177/0018720816669646 27647156 10.1177/0018720816669646

[CR93] Rabipour, S., Davidson, P. S. R., & Kristjansson, E. (2018). Measuring expectations of cognitive enhancement: Item response analysis of the expectation assessment scale. *Journal of Cognitive Enhancement*, *2*, 311–317. 10.1007/s41465-018-0073-4

[CR94] Rabipour, S., Morrison, C., Crompton, J., Petrucelli, M., Germano, M. O. G., Popescu, A., & Davidson, P. S. R. (2019). Few effects of a 5-week adaptive computerized cognitive training program in healthy older adults. *Journal of Cognitive Enhancement*, *4*, 258–273. 10.1007/s41465-019-00147-2

[CR95] Redick, T. S., Wiemers, E. A., & Engle, R. W. (2019). The role of proactive interference in working memory training and transfer. *Psychological Research Psychologische Forschung*, 1–20. 10.1007/s00426-019-01172-8 10.1007/s00426-019-01172-8 PMC6778494 30953133

[CR96] Rendell, P. G., & Craik, F. I. M. (2000). Virtual week and actual week: Age-related differences in prospective memory. *Applied Cognitive Psychology*, *14*, 43–62. 10.1002/acp.770

[CR97] Rendell, P. G., & Henry, J. D. (2009). A review of virtual week for prospective memory assessment: Clinical implications. *Brain Impairment*, *10*(1), 14–22. 10.1375/brim.10.1.14

[CR98] Reuter-Lorenz, P. A., Festini, S. B., & Jantz, T. K. (2016). Executive functions and neurocognitive aging. In K. W. Schaie, & S. L. Willis (Eds.), *Handbook of the psychology of aging* (8th ed., pp. 245–262). Elsevier Science Publishing Co Inc.

[CR99] Ripp, I., Emch, M., Wu, Q., Lizarraga, A., Udale, R., von Bastian, C. C., Koch, K., & Yakushev, I. (2022). Adaptive working memory training does not produce transfer effects in cognition and neuroimaging. *Translational Psychiatry*, *12*(512), 1–13. 10.1038/s41398-022-02272-7 36513642 10.1038/s41398-022-02272-7 PMC9747798

[CR100] Rose, N. S., Rendell, P. G., Hering, A., Kliegel, M., Bidelman, G. M., & Craik, F. I. M. (2015). Cognitive and neural plasticity in older adults’ prospective memory following training with the virtual week computer game. *Frontiers in Human Neuroscience*, *9*, 592–607. 10.3389/fnhum.2015.00592 26578936 10.3389/fnhum.2015.00592 PMC4623669

[CR101] Rouder, J. N., Morey, R. D., Speckman, P. L., & Province, J. M. (2012). Default Bayes factors for ANOVA designs. *Journal of Mathematical Psychology*, *56*(5), 356–374. 10.1016/j.jmp.2012.08.001

[CR102] Rueda, M. R., Cómbita, L. M., & Pozuelos, J. P. (2021). Cognitive training in childhood and adolescence. In T. Strobach, & J. Karbach (Eds.), *Cognitive training: An overview of features and applications* (2nd ed., pp. 127–154). Springer.

[CR103] Sabatini, S., Ukoumunne, O. C., Ballard, C., Collins, R., Anstey, K. J., Diehl, M., & Clare, L. (2021). Cross-sectional association between objective cognitive performance and perceived age-related gains and losses in cognition. *International Psychogeriatrics*, *33*(7), 727–741. 10.1017/S1041610221000375 33849677 10.1017/S1041610221000375

[CR104] Sala, G., & Gobet, F. (2019). Cognitive training does not enhance general cognition. *Trends in Cognitive Sciences*, *23*, 9–20. 10.1016/j.tics.2018.10.004 30471868 10.1016/j.tics.2018.10.004

[CR105] Sala, G., Aksayli, N. D., Tatlidil, K. S., Gondo, Y., & Gobet, F. (2019). Working memory training does not enhance older adults’ cognitive skills: A comprehensive meta-analysis. *Intelligence*, *77*, 1–13. 10.1016/j.intell.2019.101386

[CR106] Salminen, T., Kühn, S., Frensch, P. A., & Schubert, T. (2016). Transfer after dual n-back training depends on striatal activation change. *Journal of Neuroscience Methods*, *36*, 10198–10213. 10.1523/JNEUROSCI.2305-15.2016 10.1523/JNEUROSCI.2305-15.2016 PMC6705577 27683914

[CR107] Schmiedek, F. (2021). Methods and designs. In T. Strobach, & J. Karbach (Eds.), *Cognitive training: An overview of features and applications* (2nd ed., pp. 11–23). Springer.

[CR108] Schwarz, K. A., Pfister, R., & Büchel, C. (2016). Rethinking explicit expectations: Connecting placebos, social cognition, and contextual perception. *Trends in Cognitive Sciences*, *20*(6), 469–480. 10.1016/j.tics.2016.04.001 27108268 10.1016/j.tics.2016.04.001

[CR109] Shah, T., Verdile, G., Sohrabi, H., Campbell, A., Putland, E., Cheetham, C., Dhaliwal, S., Weinborn, M., Maruff, P., & Darby, D. (2014). A combination of physical activity and computerized brain training improves verbal memory and increases cerebral glucose metabolism in the elderly. *Translational Psychiatry*, *4*, e487. 10.1038/tp.2014.122 25463973 10.1038/tp.2014.122 PMC4270308

[CR110] Sheppard, D. P., Matchanova, A., Sullivan, K. L., Kazimi, S. I., & Woods, S. P. (2019). Prospective memory partially mediates the association between aging and everyday functioning. *The Clinical Neuropsychologist*, 1–20. 10.1080/13854046.2019.1637461 10.1080/13854046.2019.1637461 PMC6957765 31304859

[CR111] Steptoe, A., Deaton, A., & Stone, A. A. (2015). Psychological wellbeing, health and ageing. *Lancet*, *385*(9968), 640–648. 10.1016/S0140-6736(13)61489-0 25468152 10.1016/S0140-6736(13)61489-0 PMC4339610

[CR112] Strauss, E., Sherman, E. M. S., & Spreen, O. (2006). *A compendium of neuropsychological tests: Administration, norms, and commentary* (3rd revised ed.). Oxford University Press Inc.

[CR114] Sullivan, M. J., Edgley, K., & Dehoux, E. (1990). A survey of multiple sclerosis: I. Perceived cognitive problems and compensatory strategy use. *Canadian Journal of Rehabilitation*, *4*(2), 99–105.

[CR115] Sutin, A. R., Luchetti, M., & Terracciano, A. (2021). Sense of purpose in life and healthier cognitive aging. *Trends in Cognitive Sciences*, *25*(11), 917–919. 10.1016/j.tics.2021.08.009 34538721 10.1016/j.tics.2021.08.009 PMC8987293

[CR116] Taatgen, N. A. (2013). The nature and transfer of cognitive skills. *Psychological Review*, *120*, 439–471. 10.1037/a0033138 23750831 10.1037/a0033138

[CR117] Taatgen, N. A. (2021). Theoretical models of training and transfer effects. In T. Strobach, & J. Karbach (Eds.), *Cognitive training: An overview of features and applications* (2nd ed., pp. 41–54). Springer.

[CR119] Todd, J. A. M., Andrews, G., & Conlon, E. G. (2019). Relational thinking in later adulthood. *Psychology and Aging*, *34*(4), 486–501. 10.1037/pag0000346 30973240 10.1037/pag0000346

[CR118] Tse, Z. C. K., Cao, Y., Ogilvie, J. M., Chau, B. K. H., Ng, D. H. C., & Shum, D. H. K. (2023). Prospective memory training in older adults: A systematic review and meta-analysis. *Neuropsychology Review*, *33*(2), 347–372. 10.1007/s11065-022-09536-5 35543836 10.1007/s11065-022-09536-5 PMC10148783

[CR120] Turnbull, A., Seitz, A. R., Tadin, D., & Lin, F. V. (2022). Unifying framework for cognitive training interventions in brain aging. *Ageing Research Reviews*, *101724*, 1–11. 10.1016/j.arr.2022.101724 10.1016/j.arr.2022.101724 PMC10681332 36031055

[CR121] Tusch, E. S., Alperin, B. R., Ryan, E., Holcomb, P. J., Mohammed, A. H., & Daffner, K. R. (2016). Changes in neural activity underlying working memory after computerized cognitive training in older adults. *Frontiers in Aging Neuroscience*, *8*, 255–269. 10.3389/fnagi.2016.00255 27877122 10.3389/fnagi.2016.00255 PMC5099139

[CR122] United Nations (2022). *World Population Prospects 2022: Summary of Results* (UN DESA/POP/2022/TR/NO.3). Retrieved from: https://www.un.org/development/desa/pd/sites/www.un.org.development.desa.pd/files/wpp2022_summary_of_results.pdf

[CR124] Vodyanyk, M., Cochrane, A., Corriveau, A., Demko, Z., & Green, C. S. (2021). No evidence for expectation effects in cognitive training tasks. *Journal of Cognitive Enhancement*, *5*, 296–310. 10.1007/s41465-021-00207-6

[CR125] von Bastian, C. C., Langer, N., Jancke, L., & Oberauer, K. (2013). Effects of working memory training in young and old adults. *Memory and Cognition*, *41*, 611–624. 10.3758/s13421-012-0280-7 23263879 10.3758/s13421-012-0280-7

[CR126] Wagenmakers, E., Love, J., Marsman, M., Jamil, T., Ly, A., Verhagen, J., Selker, R., Gronau, Q. F., Dropmann, D., & Boutin, B. (2018). Bayesian inference for psychology, part II: Example applications with JASP. *Psychonomic Bulletin & Review*, *25*, 58–76. 10.3758/s13423-017-1323-7 28685272 10.3758/s13423-017-1323-7 PMC5862926

[CR127] Walton, C. C., Lampit, A., Boulamatsis, C., Hallock, H., Barr, P., Ginige, J. A., Brodaty, H., Chau, T., Heffernan, M., & Sachdev, P. S. (2019). Design and development of the brain training system for the digital maintain your brain dementia prevention trial. *JMIR Aging*, *2*(1), e13135. 31518277 10.2196/13135 PMC6715098

[CR129] Wang, Y., Cao, X. Y., Cui, J. F., Shum, D. H., & Chan, R. C. (2013). The relation between prospective memory and working memory: Evidence from event-related potential data. *PsyCh Journal*, *2*(2), 113–121. 10.1002/pchj.24 26271181 10.1002/pchj.24

[CR128] Wang, M., Sung, H., & Liu, J. (2022). Population aging and its impact on human wellbeing in China. *Frontiers in Public Health*, *10*, 883566. 10.3389/fpubh.2022.883566 35419339 10.3389/fpubh.2022.883566 PMC8995787

[CR130] Westerhof, G. J., Nehrkorn-Bailey, A. M., Tseng, H. Y., Brothers, A., Siebert, J. S., Wurm, S., & Diehl, M. (2023). Longitudinal effects of subjective aging on health and longevity: An updated meta-analysis. *Psychology and Aging*, *38*(3), 147. 10.1037/pag0000737 36972091 10.1037/pag0000737 PMC10192139

[CR131] Wolinsky, F. D., Weg, V., Martin, M. W., Unverzagt, R., Ball, F. W., Jones, K. K., R. N., & Tennstedt, S. L. (2009). The effect of speed-of-processing training on depressive symptoms in ACTIVE. *Journals of Gerontology Series A: Biomedical Sciences and Medical Sciences*, *64*(4), 468–472. 10.1093/gerona/gln044 10.1093/gerona/gln044 PMC2657170 19181719

[CR132] World Health Organization (2022). *Ageing and health*. https://www.who.int/news-room/fact-sheets/detail/ageing-and-health

[CR133] Wurm, S., Diehl, M., Kornadt, A. E., Westerhof, G. J., & Wahl, H. W. (2017). How do views on aging affect health outcomes in adulthood and late life? Explanations for an established connection. *Developmental Review*, *46*, 27–43. 10.1016/j.dr.2017.08.002 33927468 10.1016/j.dr.2017.08.002 PMC8081396

[CR134] Yesavage, J. A., & Sheikh, J. I. (1986). Geriatric depression scale (GDS). *Clinical Gerontologist*, *5*, 165–173. 10.1300/J018v05n01_09

[CR135] Zelinski, E. M., Peters, K. D., Hindin, S., Petway, K. T., & Kennison, R. F. (2014). Evaluating the relationship between change in performance on training tasks and on untrained outcomes. *Frontiers in Human Neuroscience*, *8*, 617. 10.3389/fnhum.2014.00617 25165440 10.3389/fnhum.2014.00617 PMC4131298

[CR136] Zhang, J., Li, L. W., & McLaughlin, S. J. (2022). Psychological well-being and cognitive function among older adults in china: A population-based longitudinal study. *Journal of Aging and Health*, *34*(2), 173–183. 10.1177/08982643211036226 34510952 10.1177/08982643211036226

[CR137] Zuber, S., & Kliegel, M. (2020). Prospective memory development across the lifespan: An integrative framework. *European Psychologist*, *25*, 162–173. 10.1027/1016-9040/a000380

