# Supplementary material for: A comparison of single-domain and multidomain executive functions cognitive training for enhancing cognition and well-being in older adults
Source: Psychol Res. 2026 Mar 7;90(2):52. doi: 10.1007/s00426-026-02249-x (PMC12966223; doi:10.1007/s00426-026-02249-x)
Supplement: Supplementary file 1 — Supplementary Material 1 [file 426_2026_2249_MOESM1_ESM.docx]

**Supplementary Materials**

**Table of Contents**

[S1 Description of Active Control Games 1](#_Toc207271047)

[S2 Baseline (Pre-Test) Group Comparisons Across Outcomes 3](#_Toc207271048)

[S3 Between-Groups Comparisons of Beliefs About Activities for Cognitive Enhancement 6](#_Toc207271049)

[S4 Perceptions of Training: Descriptives & Difference Statistics 7](#_Toc207271050)

[S5 Perception of Training: Post-Hoc Group Comparisons 8](#_Toc207271051)

[S6 Within-Group (Pre-Post/Post-FU) Changes Across Outcomes 9](#_Toc207271052)

[S7 Between-Groups Comparisons Across Outcomes 12](#_Toc207271053)

[S8 Descriptive Statistics: Flanker Task 15](#_Toc207271054)

[S9 Descriptive Statistics: Task Switching Paradigm 16](#_Toc207271055)

[S10 Descriptive Statistics: Virtual Week (Prospective Memory) 17](#_Toc207271056)

[S11 Young—Older Adult Comparisons Across Cognitive Outcome Measures 18](#_Toc207271057)

[S12 Subjective Outcomes: Descriptive Statistics Across Groups and Time 20](#_Toc207271058)

[Normality Checks 21](#_Toc207271059)

[Additional Analyses 22](#_Toc207271060)

[S13 Frequentist & Bayesian Statistics: Cognitive Outcomes (Interactions & Main Effects) 24](#_Toc207271061)

[S14 Frequentist & Bayesian Statistics: Subjective Outcomes (Interactions & Main Effects) 26](#_Toc207271062)

**Table S1**

## Brief Descriptions of the Active Control Games

| **Game Genre** | **Game Name**  ***(Developer)*** | **Brief Description** |
| --- | --- | --- |
| Number-based | Make 10  *SimpleLife Studio/CO2 Labs* | A 5x5 grid of numbers is presented (numbers in each cell initially range from 1 to 3). Players must tap on adjacent (horizontally or vertically) cells that display the same number to create a cell with a greater value (e.g., tapping on two ‘1’ blocks next to each other will create a ‘2’ block). This is continued until there are no blocks with the same number adjacent to each other or the player creates a ‘10’ block (which is the goal of the game). |
|  | Number Merge  *Hub Apps & Games Studio* | A 7x7 grid of colored and numbered bubbles is presented on the screen. Players must connect all bubbles with the same number (horizontally, vertically, diagonally) to clear them from the board. After clearing bubbles from the board, more random bubbles appear from the top of the grid. Players must score a pre-fixed number of points before time runs out to earn at least one star before proceeding to the next level. |
|  | Hidden Numbers  *Absolutist Ltd* | A “busy” still of a scene is presented. Numbers from 1 through to 25 are embedded and hidden within the scene. Players must find all the numbers from 1 to 25 (in any order) by tapping on the numbers in the scene. Each level presents a different scene. The numbers within higher levels are less obvious to find. |
| Word-based | Word Search  *Playvalve* | Players are shown a grid of seemingly random letters. Above the grid, players are presented with target words. Players must find these words within the grid of letters by dragging their finger across the letters of the word within the grid. Letters must be adjacent to each other (horizontally, vertically, diagonally). As players progress through the levels, the grid becomes larger (more letters to scan through). There is no time limit in this game. |
|  | Word Scroll  *Fantasy Word Games* | A grid of letters is presented on the screen; however, the grid is constantly moving in an upward direction, presenting more letters as it moves. After a few seconds, the grid cycles through to its original position, and continues to move upwards (the letters within the level do not change as the grid circles back to its original position). Players are presented with a prompt or theme (e.g., “About Body) and a number (e.g., 0/4). This indicates to participants that players must find 4 body parts in the moving grid of letters. No information is provided about the number of letters in each word. Players must scan through the moving grid and drag their finger along adjacent letters that constitute words relevant to the theme. All words must be found within the specified time limit. If all words are found in time, players proceed to the next level. As the levels become more difficult, more words need to be found. |
|  | Word Brain 2  *MAG Interactive* | To start, players are presented with a 3x3 matrix consisting of letters. Players must unscramble the letters within the matrix to solve mystery words by sliding their finger over each letter, in order, of the word they are trying to spell. When solving words, only letters adjacent to each other (horizontally, vertically, and diagonally) can be selected. The unknown words to be solved are represented at the bottom of the screen through strings of blank boxes (where the number of boxes in each string represents the number of letters in that mystery word). Once a mystery word has been solved, used letters are cleared from the board and other mystery words for that level must be solved using the remaining letters within the matrix. Each level comprises different themes. For each puzzle, the words to be solved conform to the specified theme (presented at the top of the screen). As players progress through the levels, the matrix becomes bigger (e.g., 3x3, 4x4, 5x5, etc.) and more words are required to be solved for each level. |
| Visuospatial-based | Find the Difference  *SuperSwell* | Two seemingly identical pictures are presented side-by-side, with very slight differences between the two (e.g., something is missing or a different color in one picture relative to the other). Players must locate 5 differences in the two pictures provided. Players can zoom in and out of the picture by pinching the picture on the screen. After players spot all 5 differences, they proceed to the next level. As the game progresses, the differences become more and more tricky to spot. |
|  | Match3D  *Lion Cube Studio* | A screen filled with 3D objects is presented. Players must find matching pairs of objects that are scattered across the screen (and sometimes on top of other objects). Matching pairs of objects are dragged to the platform at the bottom of the screen to be removed from play. Once all pairs of items have been located and removed, the level is cleared and players then proceed to the next level. Level progression is associated with an increase in difficulty whereby more objects (some may look similar to others) are presented on the screen, and less time is provided to clear the level. |
|  | Block! Hexa  *BitMango* | A large randomly-shaped figure comprising small hexagonal spaces within it is presented on the screen. Below this figure are hexagonal tiles making up different, random shapes of various colors. Players must place these shapes in any of the free spaces within the larger figure. The aim of the game is to fill the larger figure with the random shapes provided. The puzzles become more difficult as players progress through the levels, as the number shapes to be fitted increase and their forms become more irregular. Further, random shapes that do not fit in the figure will also be presented as the difficulty increases. |

**Table S2**

## Older Adult Training Group Comparisons and Descriptive Statistics Across Each Outcome Measure at Baseline (Pre-Test)

| **Outcomes** | **EF** | **WM** | **AC** | **Difference** | |
| --- | --- | --- | --- | --- | --- |
|  |  |  |  | **Bayes Factor (error%)** | **Interpretation** |
| **Cognitive Outcomes** | | | | | |
| ***N*-Back** |  |  |  |  |  |
| 1-Back  (Composite) | 8.20 (3.39) | 7.43 (2.16) | 7.92 (2.66) | *BF*_incl_ = 0.18 (0.007%) | Substantial evidence for no difference |
| 2-Back  (Composite) | 5.42 (3.37) | 4.82 (2.16) | 5.00 (2.01) | *BF*_incl_ = 0.16 (0.007%) | Substantial evidence for no difference |
| 3-Back  (Composite) | 3.86 (2.63) | 3.00 (2.17) | 4.12 (1.86) | *BF*_incl_ = 0.38 (0.010%) | Ambiguous evidence for no difference |
| **Flanker Task** |  |  |  |  |  |
| Flanker Effect  (RT, ms) | 99 (55) | 96 (52) | 98 (66) | *BF*_incl_ = 0.13 (0.007%) | Substantial evidence for no difference |
| **Cued Task-Switching Paradigm** |  |  |  |  |  |
| Switch Cost  (RT, ms) | 158 (44) | 150 (43) | 147 (38) | *BF*_incl_ = 0.18 (0.008%) | Substantial evidence for no difference |
| Mixing Cost  (RT, ms) | 411 (168) | 378 (148) | 377 (152) | *BF*_incl_ = 0.16 (0.007%) | Substantial evidence for no difference |
| **WCST** |  |  |  |  |  |
| Accuracy  (Proportion Correct) | 0.64 (0.12) | 0.66 (0.20) | 0.62 (0.20) | *BF*_incl_ = 0.16 (0.007%) | Substantial evidence for no difference |
| Number of Perseveration Errors | 13.68 (4.88) | 11.68 (7.19) | 12.55 (7.01) | *BF*_incl_ = 0.19 (0.008%) | Substantial evidence for no difference |
| Number of Categories Completed | 3.18 (0.96) | 3.23 (1.23) | 3.18 (1.26) | *BF*_incl_ = 0.13 (0.007%) | Substantial evidence for no difference |
| **Virtual Week** |  |  |  |  |  |
| Regular Tasks  (Proportion Correctly Completed) | 0.44 (0.23) | 0.44 (0.15) | 0.49 (0.15) | *BF*_incl_ = 0.21 (0.008%) | Substantial evidence for no difference |
| Irregular Tasks  (Proportion Correctly Completed) | 0.35 (0.17) | 0.36 (0.17) | 0.34 (0.17) | *BF*_incl_ = 0.13 (0.007%) | Substantial evidence for no difference |
| Time-Based Tasks  (Proportion Correctly Completed) | 0.35 (0.23) | 0.37 (0.25) | 0.46 (0.20) | *BF*_incl_ = 0.41 (0.009%) | Ambiguous evidence for no difference |
| **CFIT** |  |  |  |  |  |
| Accuracy  (Proportion Correct) | 0.41 (0.09) | 0.43 (0.08) | 0.43 (0.07) | *BF*_incl_ = 0.24 (0.008%) | Substantial evidence for no difference |
| Completion  (Proportion Completed) | 0.72 (0.11) | 0.72 (0.12) | 0.72 (0.10) | *BF*_incl_ = 0.13 (0.007%) | Substantial evidence for no difference |
| **Subjective Measures of Cognition and Well-being** | | | | | |
| **Subjective Ratings of Cognitive Functioning** |  |  |  |  |  |
| Attention | 6.50 (1.79) | 6.64 (1.68) | 6.68 (1.13) | *BF*_incl_ = 0.13 (0.007%) | Substantial evidence for no difference |
| Everyday Tasks | 8.64 (1.14) | 8.59 (1.33) | 8.05 (1.25) | *BF*_incl_ = 0.40 (0.009%) | Ambiguous evidence for no difference |
| Memory | 6.41 (1.56) | 6.55 (2.09) | 5.82 (1.62) | *BF*_incl_ = 0.28 (0.008%) | Substantial evidence for no difference |
| Multitasking | 7.41 (2.54) | 7.14 (2.27) | 7.73 (1.78) | *BF*_incl_ = 0.17 (0.007%) | Substantial evidence for no difference |
| Reasoning | 8.23 (1.31) | 7.82 (1.37) | 8.00 (1.20) | *BF*_incl_ = 0.19 (0.008%) | Substantial evidence for no difference |
| Speed & RT | 7.64 (1.43) | 8.18 (1.56) | 7.09 (1.77) | *BF*_incl_ = 0.86 (0.011%) | Ambiguous evidence for no difference |
| **PDQ** |  |  |  |  |  |
| PDQ  (Attention) | 8.41 (3.45) | 8.95 (3.50) | 7.05 (3.18) | *BF*_incl_ = 0.51 (0.010%) | Ambiguous evidence for no difference |
| PDQ  (Retrospective Memory) | 8.05 (4.20) | 10.32 (3.83) | 8.14 (3.62) | *BF*_incl_ = 0.76 (0.011%) | Ambiguous evidence for no difference |
| PDQ  (Prospective Memory) | 8.00 (3.83) | 9.50 (4.51) | 9.64 (3.53) | *BF*_incl_ = 0.30 (0.008%) | Substantial evidence for no difference |
| Planning / Organization | 4.36 (3.62) | 4.64 (3.09) | 4.09 (2.62) | *BF*_incl_ = 0.14 (0.007%) | Substantial evidence for no difference |
| **CASP-19** |  |  |  |  |  |
| Total | 42.41 (6.43) | 43.73 (6.18) | 43.55 (6.03) | *BF*_incl_ = 0.16 (0.007%) | Substantial evidence for no difference |
| **GDS-15** |  |  |  |  |  |
| Total | 3.05 (1.46) | 2.45 (0.86) | 2.45 (0.80) | *BF*_incl_ = 0.64 (0.010%) | Ambiguous evidence for no difference |

*Note.* Values represent mean and standard deviation. Bayes Factors (*BF*_incl_, error%) presented to evaluate differences across groups.

**Table S3**

## Descriptive Statistics and Older Adult Training Group Comparisons for Beliefs About Activities for Cognitive Enhancement

| **Variable** | **Total** | **EF** | **WM** | **AC** | **Difference** | |
| --- | --- | --- | --- | --- | --- | --- |
|  |  |  |  |  | **NHST Statistic** | **Bayes Factor (error %)** |
| Crossword puzzles | 3.71 (1.08) | 3.68 (1.17) | 3.77 (1.02) | 3.68 (1.09) | *F*(2,63) = 0.51, *p* = .951, η_p_^2^ = .002 | *BF*_incl_ = 0.13 (0.007%)  (substantial evidence)^nd^ |
| Sudoku puzzles | 3.47 (1.33) | 3.73 (1.28) | 3.18 (1.26) | 3.50 (1.44) | *F*(2,63) = 0.94, *p* = .398, η_p_^2^ = .029 | *BF*_incl_ = 0.25 (0.008%)  (substantial evidence)^nd^ |
| Brain training games | 3.67 (1.24) | 3.64 (1.29) | 3.73 (1.28) | 3.65 (1.22) | *F*(2,63) = 0.04, *p* = .963, η_p_^2^ = .001 | *BF*_incl_ = 0.13 (0.007%)  (substantial evidence)^nd^ |
| Mobile/web games | 2.97 (1.25) | 2.77 (1.38) | 3.18 (1.30) | 2.95 (1.09) | *F*(2,63) = 0.58, *p* = .562, η_p_^2^ = .018 | *BF*_incl_ = 0.19 (0.008%)  (substantial evidence)^nd^ |
| Action video games | 2.48 (1.27) | 2.59 (1.14) | 2.41 (1.40) | 2.45 (1.30) | *F*(2,63) = 0.12, *p* = .888, η_p_^2^ = .004 | *BF*_incl_ = 0.14 (0.007%)  (substantial evidence)^nd^ |
| Card or board games | 3.38 (1.27) | 3.32 (1.36) | 3.50 (1.44) | 3.32 (1.04) | *F*(2,63) = 0.15, *p* = .865, η_p_^2^ = .005 | *BF*_incl_ = 0.14 (0.007%)  (substantial evidence)^nd^ |
| Learning a new language | 3.77 (1.17) | 3.82 (1.01) | 3.77 (1.27) | 3.73 (1.28) | *F*(2,63) = 0.03, *p* = .969, η_p_^2^ = .001 | *BF*_incl_ = 0.13 (0.007%)  (substantial evidence)^nd^ |
| Learning a musical instrument | 3.33 (1.47) | 3.41 (1.22) | 3.32 (1.62) | 3.27 (1.61) | *F*(2,63) = 0.05, *p* = .954, η_p_^2^ = .002 | *BF*_incl_ = 0.13 (0.007%)  (substantial evidence)^nd^ |
| Learning how to code | 3.24 (1.35) | 3.32 (1.43) | 3.27 (1.28) | 3.14 (1.39) | *F*(2,63) = 0.11, *p* = .900, η_p_^2^ = .003 | *BF*_incl_ = 0.14 (0.007%)  (substantial evidence)^nd^ |
| Physical exercise | 3.94 (1.21) | 3.91 (1.38) | 4.05 (1.21) | 3.86 (1.08) | *F*(2,63) = 0.13, *p* = .878, η_p_^2^ = .004 | *BF*_incl_ = 0.14 (0.007%)  (substantial evidence)^nd^ |

*Note.* Descriptive statistic values represent means and standard deviations (in parentheses). Null hypothesis significance testing statistics (*p*-value) and Bayes Factors (*BF*_incl_) presented to evaluate differences across groups. **p* < .05 (indicating significant differences between groups).

**Table S4**

## Descriptive Statistics and Analysis of Participants’ Perceptions of Training Across Groups

| **Variable** | **EF** | **WM** | **AC** | **Difference** | |
| --- | --- | --- | --- | --- | --- |
|  |  |  |  | **NHST Statistic** | **Bayes Factor (error %)** |
| **Game User Experience Satisfaction Scale (GUESS)** | | | | | |
| Usability/playability | 5.50  (0.82) | 5.49  (0.97) | 5.49  (0.68) | *F*(2,63) = 0.00, *p* = .999, η_p_^2^ = .000 | *BF*_incl_ = 0.13 (0.007%)  (substantial evidence)^nd^ |
| Enjoyment | 4.93  (1.09) | 3.49  (0.91) | 5.89  (0.76) | *F*(2,63) = 37.00, *p* < .001, η_p_^2^ = .540* | *BF*_incl_ = 3.25×10^8^ (0.000%)  (decisive evidence)^d^ |
| Personal gratification | 5.51  (0.89) | 4.96  (1.33) | 3.99  (0.93) | *F*(2,63) = 11.44, *p* < .001, η_p_^2^ = .266* | *BF*_incl_ = 406.27 (0.012%)  (decisive evidence)^d^ |
| **Subjective Training-Related Improvements** | | | | | |
| Attention | 3.73  (1.08) | 3.55  (1.01) | 3.41  (1.10) | *F*(2,63) = 0.50, *p* = .611, η_p_^2^ = .016 | *BF*_incl_ = 0.18 (0.008%)  (substantial evidence)^nd^ |
| Everyday abilities | 3.64  (0.95) | 3.36  (1.05) | 2.55  (0.86) | *F*(2,63) = 7.75, *p* < .001, η_p_^2^ = .197* | *BF*_incl_ = 34.48 (0.006%)  (very strong evidence)^d^ |
| Hand-eye coordination | 3.32  (0.89) | 3.18  (1.14) | 3.23  (1.27) | *F*(2,63) = 0.09, *p* = .918, η_p_^2^ = .003 | *BF*_incl_ = 0.13 (0.007%)  (substantial evidence)^nd^ |
| Memory | 3.73  (1.03) | 4.32  (0.84) | 2.96  (1.25) | *F*(2,63) = 9.25, *p* < .001, η_p_^2^ = .227* | *BF*_incl_ = 95.56 (0.005%)  (very strong evidence)^d^ |
| Multitasking | 3.55  (1.10) | 2.68  (1.21) | 2.73  (0.94) | *F*(2,63) = 4.39, *p* = .016, η_p_^2^ = .122* | *BF*_incl_ = 3.21 (0.006%)  (substantial evidence)^d^ |
| Perception | 3.64  (1.09) | 3.23  (1.19) | 3.45  (1.14) | *F*(2,63) = 0.71, *p* = .497, η_p_^2^ = .022 | *BF*_incl_ = 0.21 (0.008%)  (substantial evidence)^nd^ |
| Reasoning | 3.00  (1.31) | 2.50  (1.01) | 2.55  (1.06) | *F*(2,63) = 1.31, *p* = .277, η_p_^2^ = .040 | *BF*_incl_ = 0.32 (0.009%)  (substantial evidence)^nd^ |
| Speed & reaction time | 4.14  (0.94) | 3.36  (1.09) | 3.05  (0.84) | *F*(2,63) = 7.44, *p* = .001, η_p_^2^ = .191* | *BF*_incl_ = 27.81 (0.006%)  (strong evidence)^d^ |

*Note.* Values represent mean and standard deviation. Null hypothesis significance testing statistics (*F*-test, p-value) and Bayes Factors (*BF*_incl_, error%) presented to evaluate differences across groups. **p* < .05 (indicating significant differences between groups).

**Table S5**

## Analysis of Group Differences in Training Perception Outcomes for Older Adults

|  | **EF – WM Difference** | **EF – AC Difference** | **WM – AC Difference** |
| --- | --- | --- | --- |
| **Game User Experience Satisfaction Scale (GUESS)** | | | |
| Usability | *M*_diff_ = 0.01, *SE* = 0.25, *p* = .999  *Odds*_posterior_ = 0.18 (substantial)^nd^ | *M*_diff_ = 0.01, *SE* = 0.25, *p* = .999  *Odds*_posterior_ = 0.18 (substantial)^nd^ | *M*_diff_ = -0.00, *SE* = 0.25, *p* = .999  *Odds*_posterior_ = 0.18 (substantial)^nd^ |
| Enjoyment | *M*_diff_ = 1.44, *SE* = 0.28, *p* < .001*  *Odds*_posterior_ = 436.95 (decisive)^d^ | *M*_diff_ = -0.96, *SE* = 0.28, *p* = .003*  *Odds*_posterior_ = 12.86 (strong)^d^ | *M*_diff_ = -2.40, *SE* = 0.28, *p* < .001*  *Odds*_posterior_ = 5.77×10^8^ (decisive)^d^ |
| Personal gratification | *M*_diff_ = 0.54, *SE* = 0.32, *p* = .287  *Odds*_posterior_ = 0.48 (ambiguous) | *M*_diff_ = 1.52, *SE* = 0.32, *p* < .001*  *Odds*_posterior_ = 4.29×10^3^ (decisive)^d^ | *M*_diff_ = 0.98, *SE* = 0.32, *p* = .011*  *Odds*_posterior_ = 3.70 (substantial)^d^ |
| **Subjective Training-Related Improvements** | | | |
| Attention | *M*_diff_ = 0.18, *SE* = 0.32, *p* = .999  *Odds*_posterior_ = 0.20 (substantial)^nd^ | *M*_diff_ = 0.32, *SE* = 0.32, *p* = .973  *Odds*_posterior_ = 0.26 (substantial)^nd^ | *M*_diff_ = 0.14, *SE* = 0.32, *p* = .999  *Odds*_posterior_ = 0.19 (substantial)^nd^ |
| Everyday abilities | *M*_diff_ = 0.27, *SE* = 0.29, *p* = .999  *Odds*_posterior_ = 0.24 (substantial)^nd^ | *M*_diff_ = 1.09, *SE* = 0.29, *p* = .001*  *Odds*_posterior_ = 56.66 (very strong)^d^ | *M*_diff_ = 0.82, *SE* = 0.29, *p* = .018*  *Odds*_posterior_ = 3.78 (substantial)^d^ |
| Hand-eye coordination | *M*_diff_ = 0.14, *SE* = 0.34, *p* = .999  *Odds*_posterior_ = 0.19 (substantial)^nd^ | *M*_diff_ = 0.09, *SE* = 0.34, *p* = .999  *Odds*_posterior_ = 0.18 (substantial)^nd^ | *M*_diff_ = -0.05, *SE* = 0.34, *p* = .999  *Odds*_posterior_ = 0.18 (substantial)^nd^ |
| Memory | *M*_diff_ = -0.59, *SE* = 0.32, *p* = .204  *Odds*_posterior_ = 0.96 (ambiguous) | *M*_diff_ = 0.77, *SE* = 0.32, *p* = .054  *Odds*_posterior_ = 1.23 (ambiguous) | *M*_diff_ = 1.36, *SE* = 0.32, *p* < .001*  *Odds*_posterior_ = 110.32 (decisive)^d^ |
| Multitasking | *M*_diff_ = 0.86, *SE* = 0.33, *p* = .032*  *Odds*_posterior_ = 1.89 (ambiguous) | *M*_diff_ = 0.82, *SE* = 0.33, *p* = .046*  *Odds*_posterior_ = 2.66 (ambiguous) | *M*_diff_ = -0.05, *SE* = 0.33, *p* = .999  *Odds*_posterior_ = 0.18 (substantial)^nd^ |
| Perception | *M*_diff_ = 0.41, *SE* = 0.35, *p* = .720  *Odds*_posterior_ = 0.31 (substantial)^nd^ | *M*_diff_ = 0.18, *SE* = 0.35, *p* = .999  *Odds*_posterior_ = 0.20 (substantial)^nd^ | *M*_diff_ = -0.23, *SE* = 0.35, *p* = .999  *Odds*_posterior_ = 0.21 (substantial)^nd^ |
| Reasoning | *M*_diff_ = 0.50, *SE* = 0.34, *p* = .445  *Odds*_posterior_ = 0.39 (ambiguous) | *M*_diff_ = 0.46, *SE* = 0.34, *p* = .565  *Odds*_posterior_ = 0.33 (ambiguous) | *M*_diff_ = -0.05, *SE* = 0.34, *p* = .999  *Odds*_posterior_ = 0.18 (substantial)^nd^ |
| Speed & RT | *M*_diff_ = 0.77, *SE* = 0.29, *p* = .030*  *Odds*_posterior_ = 2.02 (ambiguous) | *M*_diff_ = 1.09, *SE* = 0.29, *p* = .001*  *Odds*_posterior_ = 66.05 (very strong)^d^ | *M*_diff_ = 0.32, *SE* = 0.29, *p* = .835  *Odds*_posterior_ = 0.28 (substantial)^nd^ |

*Note.* **p* < .05 (indicating significant difference in performance between groups), ^d^*Odds*_posterior_ > 3 (indicating at least substantial evidence for difference between groups), ^nd^*Odds*_posterior_ < 0.33 (indicating at least substantial evidence for no difference between groups). *p-*values reflect Bonferroni-adjusted values and posterior *Odds* reflect multiplicity-adjusted values. The posterior *Odds* are the result of multiplying the prior *Odds* by the uncorrected Bayes Factor and reflect the relative plausibility of models after observing data (Goss-Sampson et al., 2020).

**Table S6**

## Analysis of Pre-Post and Post-FollowUp Change Scores for Cognitive and Subjective Outcomes for Older Adult Training Groups

|  | **EF Group** | **WM Group** | **AC Group** |
| --- | --- | --- | --- |
| **Cognitive Outcomes** | | | |
| ***N*-Back** |  |  |  |
| 1-Back (Composite) |  |  |  |
| Pre-Post Change | *M*_diff_ = 3.56, *SE* = 0.67, *p* < .001*  *Odds*_posterior_ = 3.29×10^3^ (decisive)^d^ | *M*_diff_ = 3.80, *SE* = 0.67, *p* < .001*  *Odds*_posterior_ = 419.79 (decisive)^d^ | *M*_diff_ = 0.82, *SE* = 0.67, *p* = .228  *Odds*_posterior_ = 2.29 (ambiguous) |
| 2-Back (Composite) |  |  |  |
| Pre-Post Change | *M*_diff_ = 3.90, *SE* = 0.63, *p* < .001*  *Odds*_posterior_ = 1.26×10^3^ (decisive)^d^ | *M*_diff_ = 4.10, *SE* = 0.63, *p* < .001*  *Odds*_posterior_ = 1.27×10^3^ (decisive)^d^ | *M*_diff_ = 0.85, *SE* = 0.63, *p* = .185  *Odds*_posterior_ = 0.71 (ambiguous) |
| 3-Back(Composite) |  |  |  |
| Pre-Post Change | *M*_diff_ = 4.19, *SE* = 0.65, *p* < .001*  *Odds*_posterior_ = 7.90×10^3^ (decisive)^d^ | *M*_diff_ = 4.52, *SE* = 0.65, *p* < .001*  *Odds*_posterior_ = 2.17×10^3^ (decisive)^d^ | *M*_diff_ = 0.04, *SE* = 0.65, *p* = .950  *Odds*_posterior_ = 0.22 (substantial)^nd^ |
| **Flanker Task** |  |  |  |
| Flanker Effect (RT, ms) |  |  |  |
| Pre-Post Change | *M*_diff_ = -47, *SE* = 9, *p* < .001*  *Odds*_posterior_ = 98.36 (very strong)^d^ | *M*_diff_ = -15, *SE* = 9, *p* = .082  *Odds*_posterior_ = 1.02 (ambiguous) | *M*_diff_ = -18, *SE* = 9, *p* = .045*  *Odds*_posterior_ = 1.11 (ambiguous) |
| **Cued Task-Switching Paradigm** |  |  |  |
| Switch Cost (RT, ms) |  |  |  |
| Pre-Post Change | *M*_diff_ = -42, *SE* = 7, *p* < .001*  *Odds*_posterior_ = 23.35 (strong)^d^ | *M*_diff_ = -8, *SE* = 7, *p* = .266  *Odds*_posterior_ = 0.54 (ambiguous) | *M*_diff_ = -3, *SE* = 7, *p* = .652  *Odds*_posterior_ = 0.30 (substantial)^nd^ |
| Mixing Cost (RT, ms) |  |  |  |
| Pre-Post Change | *M*_diff_ = -143, *SE* = 19, *p* < .001*  *Odds*_posterior_ = 866.29 (decisive)^d^ | *M*_diff_ = -22, *SE* = 19, *p* = .262  *Odds*_posterior_ = 0.59 (ambiguous) | *M*_diff_ = -12, *SE* = 19, *p* = .517  *Odds*_posterior_ = 0.42 (ambiguous) |
| **WCST** |  |  |  |
| Accuracy (Proportion Correct) |  |  |  |
| Pre-Post Change | *M*_diff_ = 0.14, *SE* = 0.03, *p* < .001*  *Odds*_posterior_ = 552.73 (decisive)^d^ | *M*_diff_ = 0.07, *SE* = 0.03, *p* = .031*  *Odds*_posterior_ = 0.66 (ambiguous) | *M*_diff_ = 0.03, *SE* = 0.03, *p* = .421  *Odds*_posterior_ = 0.70 (ambiguous) |
| Number of Perseveration Errors |  |  |  |
| Pre-Post Change | *M*_diff_ = -4.05, *SE* = 1.47, *p* = .008*  *Odds*_posterior_ = 3.00 (ambiguous) | *M*_diff_ = -1.86, *SE* = 1.47, *p* = .210  *Odds*_posterior_ = 0.42 (ambiguous) | *M*_diff_ = -1.00, *SE* = 1.47, *p* = .499  *Odds*_posterior_ = 0.30 (substantial)^nd^ |
| Number of Categories Completed |  |  |  |
| Pre-Post Change | *M*_diff_ = 0.86, *SE* = 0.20, *p* < .001*  *Odds*_posterior_ = 99.39 (very strong)^d^ | *M*_diff_ = 0.46, *SE* = 0.20, *p* = .028*  *Odds*_posterior_ = 0.81 (ambiguous) | *M*_diff_ = 0.32, *SE* = 0.20, *p* = .121  *Odds*_posterior_ = 3.42 (substantial)^d^ |
| **Virtual Week** |  |  |  |
| Regular Tasks  (Proportion Correctly Completed) |  |  |  |
| Pre-Post Change | *M*_diff_ = 0.23, *SE* = 0.04, *p* < .001*  *Odds*_posterior_ = 1.33×10^4^ (decisive)^d^ | *M*_diff_ = 0.18, *SE* = 0.04, *p* < .001*  *Odds*_posterior_ = 75.70 (very strong)^d^ | *M*_diff_ = 0.02, *SE* = 0.04, *p* = .606  *Odds*_posterior_ = 0.27 (substantial)^nd^ |
| Irregular Tasks  (Proportion Correctly Completed) |  |  |  |
| Pre-Post Change | *M*_diff_ = 0.15, *SE* = 0.03, *p* < .001*  *Odds*_posterior_ = 2.88×10^3^ (decisive)^d^ | *M*_diff_ = 0.07, *SE* = 0.03, *p* = .010*  *Odds*_posterior_ = 3.17 (substantial)^d^ | *M*_diff_ = 0.04, *SE* = 0.03, *p* = .130  *Odds*_posterior_ = 0.66 (ambiguous) |
| Time-Based Tasks  (Proportion Correctly Completed) |  |  |  |
| Pre-Post Change | *M*_diff_ = 0.24, *SE* = 0.04, *p* < .001*  *Odds*_posterior_ = 211.57 (decisive)^d^ | *M*_diff_ = 0.18, *SE* = 0.04, *p* < .001*  *Odds*_posterior_ = 234.88 (decisive)^d^ | *M*_diff_ = 0.05, *SE* = 0.04, *p* = .286  *Odds*_posterior_ = 0.45 (ambiguous) |
| **CFIT** |  |  |  |
| Accuracy (Proportion Correct) |  |  |  |
| Pre-Post Change | *M*_diff_ = 0.05, *SE* = 0.02, *p* = .056  *Odds*_posterior_ = 1.45 (ambiguous) | *M*_diff_ = 0.01, *SE* = 0.02, *p* = .219  *Odds*_posterior_ = 0.26 (substantial)^nd^ | *M*_diff_ = 0.05, *SE* = 0.02, *p* = .032*  *Odds*_posterior_ = 2.06 (ambiguous) |
| Completion (Proportion Completed) |  |  |  |
| Pre-Post Change | *M*_diff_ = -0.00, *SE* = 0.03, *p* = .944  *Odds*_posterior_ = 0.22 (substantial)^nd^ | *M*_diff_ = 0.01, *SE* = 0.03, *p* = .820  *Odds*_posterior_ = 0.23 (substantial)^nd^ | *M*_diff_ = -0.01, *SE* = 0.03, *p* = .662  *Odds*_posterior_ = 0.24 (substantial)^nd^ |
| **Subjective Outcomes** |  |  |  |
| **Subjective Ratings of Cognitive Functioning** | | | |
| **Attention** |  |  |  |
| Pre-Post Change | *M*_diff_ = 0.96, *SE* = 0.29, *p* = .005*  *Odds*_posterior_ = 4.87 (substantial)^d^ | *M*_diff_ = 1.05, *SE* = 0.29, *p* = .002*  *Odds*_posterior_ = 22.77 (strong)^d^ | *M*_diff_ = 0.27, *SE* = 0.29, *p* = .999  *Odds*_posterior_ = 0.40 (ambiguous) |
| Post-FollowUp Change | *M*_diff_ = 0.94, *SE* = 0.33, *p* = .018*  *Odds*_posterior_ = 2.78 (ambiguous) | *M*_diff_ = -0.41, *SE* = 0.33, *p* = .657  *Odds*_posterior_ = 0.42 (ambiguous) | *M*_diff_ = 0.32, *SE* = 0.33, *p* = .999  *Odds*_posterior_ = 0.42 (ambiguous) |
| **Everyday Tasks** |  |  |  |
| Pre-Post Change | *M*_diff_ = -0.14, *SE* = 0.17, *p* = .999  *Odds*_posterior_ = 0.28 (substantial)^nd^ | *M*_diff_ = 0.14, *SE* = 0.17, *p* = .999  *Odds*_posterior_ = 0.51 (ambiguous) | *M*_diff_ = -0.09, *SE* = 0.17, *p* = .999  *Odds*_posterior_ = 0.25 (substantial)^nd^ |
| Post-FollowUp Change | *M*_diff_ = 0.24, *SE* = 0.22, *p* = .824  *Odds*_posterior_ = 0.47 (ambiguous) | *M*_diff_ = -0.23, *SE* = 0.22, *p* = .927  *Odds*_posterior_ = 0.47 (ambiguous) | *M*_diff_ = -0.09, *SE* = 0.22, *p* = .999  *Odds*_posterior_ = 0.23 (substantial)^nd^ |
| **Memory** |  |  |  |
| Pre-Post Change | *M*_diff_ = 0.77, *SE* = 0.31, *p* = .047*  *Odds*_posterior_ = 7.41 (substantial)^d^ | *M*_diff_ = 1.46, *SE* = 0.31, *p* < .001*  *Odds*_posterior_ = 93.46 (very strong)^d^ | *M*_diff_ = -0.32, *SE* = 0.31, *p* = .931  *Odds*_posterior_ = 0.34 (ambiguous) |
| Post-FollowUp Change | *M*_diff_ = 0.46, *SE* = 0.38, *p* = .701  *Odds*_posterior_ = 0.59 (ambiguous) | *M*_diff_ = -0.59, *SE* = 0.38, *p* = .374  *Odds*_posterior_ = 0.67 (ambiguous) | *M*_diff_ = 0.59, *SE* = 0.38, *p* = .374  *Odds*_posterior_ = 0.48 (ambiguous) |
| **Multitasking** |  |  |  |
| Pre-Post Change | *M*_diff_ = 0.68, *SE* = 0.20, *p* = .003*  *Odds*_posterior_ = 2.65 (ambiguous) | *M*_diff_ = 0.27, *SE* = 0.20, *p* = .527  *Odds*_posterior_ = 2.00 (ambiguous) | *M*_diff_ = -0.09, *SE* = 0.20, *p* = .999  *Odds*_posterior_ = 0.25 (substantial)^nd^ |
| Post-FollowUp Change | *M*_diff_ = -0.12, *SE* = 0.27, *p* = .999  *Odds*_posterior_ = 0.25 (substantial)^nd^ | *M*_diff_ = -0.68, *SE* = 0.27, *p* = .044*  *Odds*_posterior_ = 0.95 (ambiguous) | *M*_diff_ = -0.05, *SE* = 0.27, *p* = .999  *Odds*_posterior_ = 0.23 (substantial)^nd^ |
| **Reasoning** |  |  |  |
| Pre-Post Change | *M*_diff_ = 0.14, *SE* = 0.19, *p* = .999  *Odds*_posterior_ = 0.32 (substantial)^nd^ | *M*_diff_ = 0.32, *SE* = 0.19, *p* = .269  *Odds*_posterior_ = 0.51 (ambiguous) | *M*_diff_ = 0.23, *SE* = 0.19, *p* = .668  *Odds*_posterior_ = 0.54 (ambiguous) |
| Post-FollowUp Change | *M*_diff_ = -0.19, *SE* = 0.27, *p* = .999  *Odds*_posterior_ = 0.35 (ambiguous) | *M*_diff_ = -0.32, *SE* = 0.27, *p* = .717  *Odds*_posterior_ = 0.59 (ambiguous) | *M*_diff_ = 0.05, *SE* = 0.27, *p* = .999  *Odds*_posterior_ = 0.23 (substantial)^nd^ |
| **Speed & RT** |  |  |  |
| Pre-Post Change | *M*_diff_ = 1.05, *SE* = 0.23, *p* < .001*  *Odds*_posterior_ = 224.93 (decisive)^d^ | *M*_diff_ = 0.68, *SE* = 0.23, *p* = .015*  *Odds*_posterior_ = 2.65 (ambiguous) | *M*_diff_ = 0.27, *SE* = 0.23, *p* = .746  *Odds*_posterior_ = 0.51 (ambiguous) |
| Post-FollowUp Change | *M*_diff_ = -0.04, *SE* = 0.24, *p* = .999  *Odds*_posterior_ = 16.29 (strong)^d^ | *M*_diff_ = -0.18, *SE* = 0.24, *p* = .999  *Odds*_posterior_ = 0.27 (substantial)^nd^ | *M*_diff_ = 0.14, *SE* = 0.24, *p* = .999  *Odds*_posterior_ = 0.26 (substantial)^nd^ |
| **PDQ** | | | |
| **Attention** |  |  |  |
| Pre-Post Change | *M*_diff_ = -1.68, *SE* = 0.36, *p* < .001*  *Odds*_posterior_ = 44.74 (very strong)^d^ | *M*_diff_ = -1.05, *SE* = 0.36, *p* = .014*  *Odds*_posterior_ = 9.16 (substantial)^d^ | *M*_diff_ = 0.41, *SE* = 0.36, *p* = .762  *Odds*_posterior_ = 0.53 (ambiguous) |
| Post-FollowUp Change | *M*_diff_ = -0.23, *SE* = 0.41, *p* = .999  *Odds*_posterior_ = 0.25 (substantial)^nd^ | *M*_diff_ = -0.36, *SE* = 0.41, *p* = .999  *Odds*_posterior_ = 0.30 (substantial)^nd^ | *M*_diff_ = -0.32, *SE* = 0.41, *p* = .999  *Odds*_posterior_ = 0.40 (ambiguous) |
| **Retrospective Memory** |  |  |  |
| Pre-Post Change | *M*_diff_ = -1.18, *SE* = 0.28, *p* < .001*  *Odds*_posterior_ = 47.43 (very strong)^d^ | *M*_diff_ = -1.91, *SE* = 0.28, *p* < .001*  *Odds*_posterior_ = 5.31×10^3^ (decisive)^d^ | *M*_diff_ = 0.46, *SE* = 0.28, *p* = .328  *Odds*_posterior_ = 1.22 (ambiguous) |
| Post-FollowUp Change | *M*_diff_ = 0.41, *SE* = 0.37, *p* = .804  *Odds*_posterior_ = 0.36 (ambiguous) | *M*_diff_ = 0.14, *SE* = 0.37, *p* = .999  *Odds*_posterior_ = 0.24 (substantial)^nd^ | *M*_diff_ = -0.23, *SE* = 0.37, *p* = .999  *Odds*_posterior_ = 0.27 (substantial)^nd^ |
| **Prospective Memory** |  |  |  |
| Pre-Post Change | *M*_diff_ = -0.36, *SE* = 0.30, *p* = .666  *Odds*_posterior_ = 0.56 (ambiguous) | *M*_diff_ = -1.73, *SE* = 0.30, *p* < .001*  *Odds*_posterior_ = 271.35 (decisive)^d^ | *M*_diff_ = 0.05, *SE* = 0.30, *p* = .999  *Odds*_posterior_ = 0.23 (substantial)^nd^ |
| Post-FollowUp Change | *M*_diff_ = -0.36, *SE* = 0.42, *p* = .999  *Odds*_posterior_ = 0.46 (ambiguous) | *M*_diff_ = 0.55, *SE* = 0.42, *p* = .580  *Odds*_posterior_ = 0.43 (ambiguous) | *M*_diff_ = -0.09, *SE* = 0.42, *p* = .999  *Odds*_posterior_ = 0.23 (substantial)^nd^ |
| **Planning/Organization** |  |  |  |
| Pre-Post Change | *M*_diff_ = -0.68, *SE* = 0.30, *p* = .072  *Odds*_posterior_ = 0.81 (ambiguous) | *M*_diff_ = -0.64, *SE* = 0.30, *p* = .104  *Odds*_posterior_ = 5.12 (substantial)^d^ | *M*_diff_ = -0.27, *SE* = 0.30, *p* = .999  *Odds*_posterior_ = 0.40 (ambiguous) |
| Post-FollowUp Change | *M*_diff_ = 0.05, *SE* = 0.38, *p* = .999  *Odds*_posterior_ = 0.22 (substantial)^nd^ | *M*_diff_ = -0.23, *SE* = 0.38, *p* = .999  *Odds*_posterior_ = 0.32 (substantial)^nd^ | *M*_diff_ = 0.00, *SE* = 0.38, *p* = .999  *Odds*_posterior_ = 0.22 (substantial)^nd^ |
| **CASP-19** | | | |
| **Total** |  |  |  |
| Pre-Post Change | *M*_diff_ = 2.68, *SE* = 0.70, *p* = .001*  *Odds*_posterior_ = 34.00 (very strong)^d^ | *M*_diff_ = 3.18, *SE* = 0.70, *p* < .001*  *Odds*_posterior_ = 59.67 (very strong)^d^ | *M*_diff_ = 2.09, *SE* = 0.70, *p* = .013  *Odds*_posterior_ = 15.41 (strong)^d^ |
| Post-FollowUp Change | *M*_diff_ = -0.91, *SE* = 0.94, *p* = .999  *Odds*_posterior_ = 0.86 (ambiguous) | *M*_diff_ = -2.09, *SE* = 0.94, *p* = .087  *Odds*_posterior_ = 1.27 (ambiguous) | *M*_diff_ = -0.23, *SE* = 0.94, *p* = .999  *Odds*_posterior_ = 0.23 (substantial)^nd^ |
| **GDS-15** | | | |
| **Total** |  |  |  |
| Pre-Post Change | *M*_diff_ = -0.32, *SE* = 0.12, *p* = .038*  *Odds*_posterior_ = 0.86 (ambiguous) | *M*_diff_ = 0.14, *SE* = 0.12, *p* = .829  *Odds*_posterior_ = 0.51 (ambiguous) | *M*_diff_ = 0.00, *SE* = 0.12, *p* = .999  *Odds*_posterior_ = 0.22 (substantial)^nd^ |
| Post-FollowUp Change | *M*_diff_ = 0.05, *SE* = ,0.15 *p* = .999  *Odds*_posterior_ = 0.24 (substantial)^nd^ | *M*_diff_ = 0.14, *SE* = 0.15, *p* = .999  *Odds*_posterior_ = 0.30 (substantial)^nd^ | *M*_diff_ = -0.14, *SE* = 0.15, *p* = .999  *Odds*_posterior_ = 0.32 (substantial)^nd^ |

*Note.* **p* < .05 (indicating significant difference in performance between groups), ^d^*Odds*_posterior_ > 3 (indicating at least substantial evidence for difference between groups), ^nd^*Odds*_posterior_ < 0.33 (indicating at least substantial evidence for no difference between groups). *p-*values reflect Bonferroni-adjusted values and posterior *Odds* reflect multiplicity-adjusted values (Goss-Sampson et al., 2020).

**Table S7**

## Analysis of Older Adult Training Group Differences in Change Scores (Pre-Post and Post-FollowUp) for Cognitive and Subjective Outcomes

|  | **EF – WM Difference** | **EF – AC Difference** | **WM – AC Difference** |
| --- | --- | --- | --- |
| **Cognitive Outcomes** | | | |
| ***N*-Back Task** |  |  |  |
| 1-Back (Composite) |  |  |  |
| Pre-Post Change | *M*_diff_ = -0.24, *SE* = 0.95, *p* = .999  *Odds*_posterior_ = 0.18 (substantial)^nd^ | *M*_diff_ = 2.74, *SE* = 0.95, *p* = .016*  *Odds*_posterior_ = 56.25 (very strong)^d^ | *M*_diff_ = 3.00, *SE* = 0.95, *p* = .008*  *Odds*_posterior_ = 5.13 (substantial)^d^ |
| 2-Back (Composite) |  |  |  |
| Pre-Post Change | *M*_diff_ = -0.20, *SE* = 0.90, *p* = .999  *Odds*_posterior_ = 0.18 (substantial)^nd^ | *M*_diff_ = 3.05, *SE* = 0.90, *p* = .003*  *Odds*_posterior_ = 15.90 (strong)^d^ | *M*_diff_ = 3.25, *SE* = 0.90, *p* = .002*  *Odds*_posterior_ = 43.42 (very strong)^d^ |
| 3-Back (Composite) |  |  |  |
| Pre-Post Change | *M*_diff_ = -0.33, *SE* = 0.91, *p* = .999  *Odds*_posterior_ = 0.18 (substantial)^nd^ | *M*_diff_ = 4.15, *SE* = 0.91, *p* < .001*  *Odds*_posterior_ = 1.26×10^3^ (decisive)^d^ | *M*_diff_ = 4.48, *SE* = 0.91, *p* < .001*  *Odds*_posterior_ = 741.66 (decisive)^d^ |
| **Flanker Task** |  |  |  |
| Flanker Effect (RT, ms) |  |  |  |
| Pre-Post Change | *M*_diff_ = -31, *SE* = 12, *p* = .042*  *Odds*_posterior_ = 2.34 (ambiguous) | *M*_diff_ = -29, *SE* = 12, *p* = .069  *Odds*_posterior_ = 0.88 (ambiguous) | *M*_diff_ = 2, *SE* = 12, *p* = .999  *Odds*_posterior_ = 0.18 (substantial)^nd^ |
| **Cues Task-Switching Paradigm** |  |  |  |
| Switch Cost (RT, ms) |  |  |  |
| Pre-Post Change | *M*_diff_ = -34, *SE* = 10, *p* = .002*  *Odds*_posterior_ = 4.63 (substantial)^d^ | *M*_diff_ = -38, *SE* = 10, *p* < .001*  *Odds*_posterior_ = 11.00 (strong)^d^ | *M*_diff_ = -5, *SE* = 10, *p* = .999  *Odds*_posterior_ = 0.41 (ambiguous) |
| Mixing Cost (RT, ms) |  |  |  |
| Pre-Post Change | *M*_diff_ = -121, *SE* = 27, *p* < .001*  *Odds*_posterior_ = 56.29 (very strong)^d^ | *M*_diff_ = -130, *SE* = 27, *p* < .001*  *Odds*_posterior_ = 158.98 (decisive)^d^ | *M*_diff_ = -9, *SE* = 27, *p* = .999  *Odds*_posterior_ = 0.19 (substantial)^nd^ |
| **WCST** |  |  |  |
| Accuracy (Proportion Correct) |  |  |  |
| Pre-Post Change | *M*_diff_ = 0.07, *SE* = 0.04, *p* = .272  *Odds*_posterior_ = 0.41 (ambiguous) | *M*_diff_ = 0.12, *SE* = 0.04, *p* = .026*  *Odds*_posterior_ = 27.37 (strong)^d^ | *M*_diff_ = 0.04, *SE* = 0.04, *p* = .983  *Odds*_posterior_ = 0.25 (substantial)^nd^ |
| Number of Perseveration Errors |  |  |  |
| Pre-Post Change | *M*_diff_ = -2.18, *SE* = 2.08, *p* = .894  *Odds*_posterior_ = 0.26 (substantial)^nd^ | *M*_diff_ = -3.04, *SE* = 2.08, *p* = .444  *Odds*_posterior_ = 0.43 (ambiguous) | *M*_diff_ = -0.86, *SE* = 2.08, *p* = .999  *Odds*_posterior_ = 0.19 (substantial)^nd^ |
| Number of Categories Completed |  |  |  |
| Pre-Post Change | *M*_diff_ = 0.41, *SE* = 0.29, *p* = .474  *Odds*_posterior_ = 0.32 (substantial)^nd^ | *M*_diff_ = 0.55, *SE* = 0.29, *p* = .184  *Odds*_posterior_ = 1.45 (ambiguous) | *M*_diff_ = 0.14, *SE* = 0.29, *p* = .999  *Odds*_posterior_ = 0.19 (substantial)^nd^ |
| **Virtual Week** |  |  |  |
| Regular Tasks  (Proportion Correctly Completed) |  |  |  |
| Pre-Post Change | *M*_diff_ = 0.04, *SE* = 0.05, *p* = .999  *Odds*_posterior_ = 0.23 (substantial)^nd^ | *M*_diff_ = 0.21, *SE* = 0.05, *p* < .001*  *Odds*_posterior_ = 353.18 (decisive)^d^ | *M*_diff_ = 0.16, *SE* = 0.05, *p* = .006*  *Odds*_posterior_ = 7.19 (substantial)^d^ |
| Irregular Tasks  (Proportion Correctly Completed) |  |  |  |
| Pre-Post Change | *M*_diff_ = 0.08, *SE* = 0.04, *p* = .079  *Odds*_posterior_ = 1.24 (ambiguous) | *M*_diff_ = 0.11, *SE* = 0.04, *p* = .009*  *Odds*_posterior_ = 7.18 (substantial)^d^ | *M*_diff_ = 0.03, *SE* = 0.04, *p* = .999  *Odds*_posterior_ = 0.23 (substantial)^nd^ |
| Time-Based Tasks  (Proportion Correctly Completed) |  |  |  |
| Pre-Post Change | *M*_diff_ = 0.07, *SE* = 0.06, *p* = .788  *Odds*_posterior_ = 0.28 (substantial)^nd^ | *M*_diff_ = 0.20, *SE* = 0.06, *p* = .005*  *Odds*_posterior_ = 6.64 (substantial)^d^ | *M*_diff_ = 0.13, *SE* = 0.06, *p* = .107  *Odds*_posterior_ = 1.90 (ambiguous) |
| **CFIT** |  |  |  |
| Accuracy (Proportion Correct) |  |  |  |
| Pre-Post Change | *M*_diff_ = 0.03, *SE* = 0.03, *p* = .999  *Odds*_posterior_ = 0.24 (substantial)^nd^ | *M*_diff_ = -0.01, *SE* = 0.03, *p* = .999  *Odds*_posterior_ = 0.18 (substantial)^nd^ | *M*_diff_ = -0.03, *SE* = 0.03, *p* = .835  *Odds*_posterior_ = 0.27 (substantial)^nd^ |
| Completion (Proportion Completed) |  |  |  |
| Pre-Post Change | *M*_diff_ = -0.01, *SE* = 0.04, *p* = .999  *Odds*_posterior_ = 0.18 (substantial)^nd^ | *M*_diff_ = 0.01, *SE* = 0.04, *p* = .999  *Odds*_posterior_ = 0.18 (substantial)^nd^ | *M*_diff_ = 0.02, *SE* = 0.04, *p* = .999  *Odds*_posterior_ = 0.19 (substantial)^nd^ |
| **Subjective Outcomes** |  |  |  |
| **Subjective Ratings of Cognitive Functioning** | | | |
| **Attention** |  |  |  |
| Pre-Post Change | *M*_diff_ = -0.09, *SE* = 0.41, *p* = .999  *Odds*_posterior_ = 0.18 (substantial)^nd^ | *M*_diff_ = 0.68, *SE* = 0.41, *p* = .312  *Odds*_posterior_ = 0.51 (ambiguous) | *M*_diff_ = 0.77, *SE* = 0.41, *p* = .198  *Odds*_posterior_ = 0.92 (ambiguous) |
| Post-FollowUp Change | *M*_diff_ = 1.34, *SE* = 0.47, *p* = .016*  *Odds*_posterior_ = 2.73 (ambiguous) | *M*_diff_ = 0.62, *SE* = 0.47, *p* = .564  *Odds*_posterior_ = 0.36 (ambiguous) | *M*_diff_ = -0.73, *SE* = 0.47, *p* = .371  *Odds*_posterior_ = 0.54 (ambiguous) |
| **Everyday Tasks** |  |  |  |
| Pre-Post Change | *M*_diff_ = -0.27, *SE* = 0.24, *p* = .764  *Odds*_posterior_ = 0.32 (substantial)^nd^ | *M*_diff_ = -0.05, *SE* = 0.24, *p* = .999  *Odds*_posterior_ = 0.18 (substantial)^nd^ | *M*_diff_ = 0.23, *SE* = 0.24, *p* = .999  *Odds*_posterior_ = 0.28 (substantial)^nd^ |
| Post-FollowUp Change | *M*_diff_ = 0.47, *SE* = 0.31, *p* = .412  *Odds*_posterior_ = 0.67 (ambiguous) | *M*_diff_ = 0.33, *SE* = 0.31, *p* = .867  *Odds*_posterior_ = 0.26 (substantial)^nd^ | *M*_diff_ = -0.14, *SE* = 0.31, *p* = .999  *Odds*_posterior_ = 0.19 (substantial)^nd^ |
| **Memory** |  |  |  |
| Pre-Post Change | *M*_diff_ = -0.68, *SE* = 0.44, *p* = .378  *Odds*_posterior_ = 0.49 (ambiguous) | *M*_diff_ = 1.09, *SE* = 0.44, *p* = .047*  *Odds*_posterior_ = 2.44 (ambiguous) | *M*_diff_ = 1.77, *SE* = 0.44, *p* < .001*  *Odds*_posterior_ = 29.49 (strong)^d^ |
| Post-FollowUp Change | *M*_diff_ = 1.05, *SE* = 0.54, *p* = .167  *Odds*_posterior_ = 0.21 (substantial)^nd^ | *M*_diff_ = -0.13, *SE* = 0.54, *p* = .999  *Odds*_posterior_ = 0.96 (ambiguous) | *M*_diff_ = -1.18, *SE* = 0.54, *p* = .094  *Odds*_posterior_ = 0.25 (substantial)^nd^ |
| **Multitasking** |  |  |  |
| Pre-Post Change | *M*_diff_ = 0.41, *SE* = 0.28, *p* = .454  *Odds*_posterior_ = 0.37 (ambiguous) | *M*_diff_ = 0.77, *SE* = 0.28, *p* = .024*  *Odds*_posterior_ = 1.60 (ambiguous) | *M*_diff_ = 0.36, *SE* = 0.28, *p* = .604  *Odds*_posterior_ = 0.58 (ambiguous) |
| Post-FollowUp Change | *M*_diff_ = 0.57, *SE* = 0.38, *p* = .438  *Odds*_posterior_ = 0.34 (ambiguous) | *M*_diff_ = -0.07, *SE* = 0.38, *p* = .999  *Odds*_posterior_ = 0.18 (substantial)^nd^ | *M*_diff_ = -0.64, *SE* = 0.38, *p* = .307  *Odds*_posterior_ = 0.47 (ambiguous) |
| **Reasoning** |  |  |  |
| Pre-Post Change | *M*_diff_ = -0.18, *SE* = 0.26, *p* = .999  *Odds*_posterior_ = 0.21 (substantial)^nd^ | *M*_diff_ = 0.36, *SE* = 0.26, *p* = .506  *Odds*_posterior_ = 0.52 (ambiguous) | *M*_diff_ = 0.55, *SE* = 0.26, *p* = .122  *Odds*_posterior_ = 0.77 (ambiguous) |
| Post-FollowUp Change | *M*_diff_ = 0.14, *SE* = 0.38, *p* = .999  *Odds*_posterior_ = 0.19 (substantial)^nd^ | *M*_diff_ = -0.23, *SE* = 0.38, *p* = .999  *Odds*_posterior_ = 0.20 (substantial)^nd^ | *M*_diff_ = -0.36, *SE* = 0.38, *p* = .999  *Odds*_posterior_ = 0.23 (substantial)^nd^ |
| **Speed & RT** |  |  |  |
| Pre-Post Change | *M*_diff_ = 0.36, *SE* = 0.33, *p* = .830  *Odds*_posterior_ = 0.27 (substantial)^nd^ | *M*_diff_ = 1.32, *SE* = 0.33, *p* = .001*  *Odds*_posterior_ = 172.32 (decisive)^d^ | *M*_diff_ = 0.95, *SE* = 0.33, *p* = .016*  *Odds*_posterior_ = 3.65 (substantial)^d^ |
| Post-FollowUp Change | *M*_diff_ = 0.14, *SE* = 0.34, *p* = .999  *Odds*_posterior_ = 0.19 (substantial)^nd^ | *M*_diff_ = -0.18, *SE* = 0.34, *p* = .999  *Odds*_posterior_ = 0.20 (substantial)^nd^ | *M*_diff_ = -0.32, *SE* = 0.34, *p* = .999  *Odds*_posterior_ = 0.24 (substantial)^nd^ |
| **PDQ** |  |  |  |
| **Attention** |  |  |  |
| Pre-Post Change | *M*_diff_ = -0.64, *SE* = 0.50, *p* = .630  *Odds*_posterior_ = 0.30 (substantial)^nd^ | *M*_diff_ = -2.09, *SE* = 0.50, *p* < .001*  *Odds*_posterior_ = 63.68 (very strong)^d^ | *M*_diff_ = -1.45, *SE* = 0.50, *p* = .016*  *Odds*_posterior_ = 10.25 (strong)^d^ |
| Post-FollowUp Change | *M*_diff_ = 0.14, *SE* = 0.58, *p* = .999  *Odds*_posterior_ = 0.18 (substantial)^nd^ | *M*_diff_ = 0.09, *SE* = 0.58, *p* = .999  *Odds*_posterior_ = 0.18 (substantial)^nd^ | *M*_diff_ = -0.05, *SE* = 0.58, *p* = .999  *Odds*_posterior_ = 0.18 (substantial)^nd^ |
| Retrospective Memory |  |  |  |
| Pre-Post Change | *M*_diff_ = 0.73, *SE* = 0.40, *p* = .213  *Odds*_posterior_ = 0.55 (ambiguous) | *M*_diff_ = -1.64, *SE* = 0.40, *p* < .001*  *Odds*_posterior_ = 156.22 (decisive)^d^ | *M*_diff_ = -2.36, *SE* = 0.40, *p* < .001*  *Odds*_posterior_ = 3.10×10^4^ (decisive)^d^ |
| Post-FollowUp Change | *M*_diff_ = 0.27, *SE* = 0.52, *p* = .999  *Odds*_posterior_ = 0.19 (substantial)^nd^ | *M*_diff_ = 0.64, *SE* = 0.52, *p* = .671  *Odds*_posterior_ = 0.32 (substantial)^nd^ | *M*_diff_ = 0.36, *SE* = 0.52, *p* = .999  *Odds*_posterior_ = 0.22 (substantial)^nd^ |
| **Prospective Memory** |  |  |  |
| Pre-Post Change | *M*_diff_ = 1.36, *SE* = 0.42, *p* = .005*  *Odds*_posterior_ = 6.67 (substantial)^d^ | *M*_diff_ = -0.41, *SE* = 0.42, *p* = .990  *Odds*_posterior_ = 0.29 (substantial)^nd^ | *M*_diff_ = -1.77, *SE* = 0.42, *p* < .001*  *Odds*_posterior_ = 56.35 (very strong)^d^ |
| Post-FollowUp Change | *M*_diff_ = -0.91, *SE* = 0.59, *p* = .378  *Odds*_posterior_ = 0.57 (ambiguous) | *M*_diff_ = -0.27, *SE* = 0.59, *p* = .999  *Odds*_posterior_ = 0.19 (substantial)^nd^ | *M*_diff_ = 0.64, *SE* = 0.59, *p* = .846  *Odds*_posterior_ = 0.25 (substantial)^nd^ |
| **Planning/Organization** |  |  |  |
| Pre-Post Change | *M*_diff_ = -0.05, *SE* = 0.42, *p* = .999  *Odds*_posterior_ = 0.18 (substantial)^nd^ | *M*_diff_ = -0.41, *SE* = 0.42, *p* = .989  *Odds*_posterior_ = 0.24 (substantial)^nd^ | *M*_diff_ = -0.36, *SE* = 0.42, *p* = .999  *Odds*_posterior_ = 0.29 (substantial)^nd^ |
| Post-FollowUp Change | *M*_diff_ = 0.27, *SE* = 0.54, *p* = .999  *Odds*_posterior_ = 0.20 (substantial)^nd^ | *M*_diff_ = 0.05, *SE* = 0.54, *p* = .999  *Odds*_posterior_ = 0.18 (substantial)^nd^ | *M*_diff_ = -0.23, *SE* = 0.54, *p* = .999  *Odds*_posterior_ = 0.19 (substantial)^nd^ |
| **CASP-19** |  |  |  |
| **Total** |  |  |  |
| Pre-Post Change | *M*_diff_ = -0.50, *SE* = 1.00, *p* = .999  *Odds*_posterior_ = 0.19 (substantial)^nd^ | *M*_diff_ = 0.59, *SE* = 1.00, *p* = .999  *Odds*_posterior_ = 0.21 (substantial)^nd^ | *M*_diff_ = 1.09, *SE* = 1.00, *p* = .833  *Odds*_posterior_ = 0.28 (substantial)^nd^ |
| Post-FollowUp Change | *M*_diff_ = 1.18, *SE* = 1.32, *p* = .999  *Odds*_posterior_ = 0.27 (substantial)^nd^ | *M*_diff_ = -0.68, *SE* = 1.32, *p* = .999  *Odds*_posterior_ = 0.20 (substantial)^nd^ | *M*_diff_ = -1.86, *SE* = 1.32, *p* = .492  *Odds*_posterior_ = 0.32 (substantial)^nd^ |
| **GDS-15** |  |  |  |
| **Total** |  |  |  |
| Pre-Post Change | *M*_diff_ = -0.18, *SE* = 0.18, *p* = .913  *Odds*_posterior_ = 0.24 (substantial)^nd^ | *M*_diff_ = -0.32, *SE* = 0.18, *p* = .224  *Odds*_posterior_ = 0.53 (ambiguous) | *M*_diff_ = -0.14, *SE* = 0.18, *p* = .999  *Odds*_posterior_ = 0.30 (substantial)^nd^ |
| Post-FollowUp Change | *M*_diff_ = -0.09, *SE* = 0.21, *p* = .999  *Odds*_posterior_ = 0.19 (substantial)^nd^ | *M*_diff_ = 0.18, *SE* = 0.21, *p* = .999  *Odds*_posterior_ = 0.25 (substantial)^nd^ | *M*_diff_ = 0.27, *SE* = 0.21, *p* = .587  *Odds*_posterior_ = 0.32 (substantial)^nd^ |

*Note.* **p* < .05 (indicating significant difference in performance between groups), ^d^*Odds*_posterior_ > 3 (indicating at least substantial evidence for difference between groups), ^nd^*Odds*_posterior_ < 0.33 (indicating at least substantial evidence for no difference between groups). *p-*values reflect Bonferroni-adjusted values and posterior *Odds* reflect multiplicity-adjusted values (Goss-Sampson et al., 2020).

**Table S8**

## Descriptive Statistics for Accuracy and Reaction Time Data Across All Task Conditions and Groups for the Flanker Task

| **Flanker Task Measure** | **Older Adults** | | | | | | **Young Adults** |
| --- | --- | --- | --- | --- | --- | --- | --- |
|  | **EF Group** | | **WM Group** | | **AC Group** | |  |
|  | **Pre-Test** | **Post-Test** | **Pre-Test** | **Post-Test** | **Pre-Test** | **Post-Test** |  |
| **Accuracy** | | | | | | | |
| Congruent Trials Accuracy (%) | 99.64%  (1.33) | 99.77%  (0.75) | 99.59%  (1.40) | 99.32%  (1.84) | 99.32%  (1.29) | 99.73%  (0.88) | 99.73%  (0.76) |
| Incongruent Trials Accuracy (%) | 99.55%  (1.06) | 99.41%  (1.40) | 98.55%  (1.97) | 98.82%  (1.82) | 99.18%  (1.59) | 99.64%  (0.79) | 98.67%  (3.18) |
| **RT** | | | | | | | |
| Congruent Trials RT  (ms) | 510  (84) | 475  (76) | 525  (110) | 521  (110) | 512  (123) | 507  (118) | 389  (45) |
| Incongruent Trials RT  (ms) | 609  (72) | 528  (84) | 621  (117) | 601  (117) | 610  (128) | 588  (91) | 430  (45) |

*Note.* Values represent mean and standard deviation.

**Table S9**

## Descriptive Statistics for Accuracy and Reaction Time Data Across All Task Conditions and Groups for the Task Switching Paradigm

| **Task Switching Measure** | **Older Adults** | | | | | | **Young Adults** |
| --- | --- | --- | --- | --- | --- | --- | --- |
|  | **EF Group** | | **WM Group** | | **AC Group** | |  |
|  | **Pre-Test** | **Post-Test** | **Pre-Test** | **Post-Test** | **Pre-Test** | **Post-Test** |  |
| **Accuracy** | | | | | | | |
| Single-Task Accuracy (%) | 99.34%  (0.01) | 99.93%  (0.00) | 99.14%  (0.01) | 99.64%  (0.00) | 99.18%  (0.02) | 99.59%  (0.01) | 99.54%  (0.00) |
| Mixed-Task: Nonswitch Trials Accuracy (%) | 98.55%  (0.02) | 99.68%  (0.01) | 98.50%  (0.02) | 99.32%  (0.01) | 98.64%  (0.02) | 99.14%  (0.02) | 99.39%  (0.01) |
| Mixed-Task: Switch Trials Accuracy (%) | 98.00%  (0.02) | 99.18%  (0.01) | 98.23%  (0.02) | 99.09%  (0.02) | 99.18%  (0.02) | 99.32%  (0.02) | 98.86%  (0.02) |
| Mixed-Task Overall Accuracy (%) | 98.23%  (0.02) | 99.43%  (0.01) | 98.36%  (0.02) | 99.18%  (0.02) | 98.86%  (0.02) | 99.23%  (0.02) | 99.13%  (0.02) |
| **RT** | | | | | | | |
| Single-Task RT (ms) | 751  (98) | 696  (86) | 725  (76) | 727  (112) | 743  (113) | 739  (93) | 605  (120) |
| Mixed-Task: Nonswitch Trials RT (ms) | 1083  (214) | 906  (193) | 1028  (177) | 1012  (199) | 1048  (139) | 1032  (153) | 779  (235) |
| Mixed-Task: Switch Trials RT (ms) | 1241  (213) | 1022  (194) | 1178  (167) | 1154  (183) | 1194  (148) | 1175  (165) | 833  (236) |
| Mixed-Task Overall RT (ms) | 1162  (212) | 964  (193) | 1103  (171) | 1083  (190) | 1121  (142) | 1104  (158) | 806  (225) |

*Note.* Values represent mean and standard deviation.

**Table S10**

## Descriptive Statistics Showing Performance on the Virtual Week (Prospective Memory) Task Across Groups and Testing Occasion

| **Task Type** | **EF Group** | | **WM Group** | | **AC Group** | | **Young Adult Group** |
| --- | --- | --- | --- | --- | --- | --- | --- |
|  | **Pre-Test** | **Post-Test** | **Pre-Test** | **Post-Test** | **Pre-Test** | **Post-Test** |  |
| **Regular Task** |  |  |  |  |  |  |  |
| Correct (on-time) | 0.44 (0.23) | 0.66 (0.17) | 0.44 (0.15) | 0.62 (0.19) | 0.49 (0.15) | 0.51 (0.16) | 0.84 (0.17) |
| Early | 0.03 (0.05) | 0.01 (0.02) | 0.02 (0.04) | 0.00 (0.00) | 0.02 (0.04) | 0.00 (0.02) | 0.03 (0.08) |
| Late | 0.14 (0.11) | 0.06 (0.10) | 0.17 (0.12) | 0.16 (0.08) | 0.14 (0.10) | 0.15 (0.13) | 0.07 (0.13) |
| Miss | 0.39 (0.17) | 0.26 (0.17) | 0.37 (0.14) | 0.22 (0.16) | 0.33 (0.14) | 0.34 (0.16) | 0.06 (0.11) |
| Incorrect | 0.02 (0.04) | 0.01 (0.04) | 0.00 (0.02) | 0.00 (0.00) | 0.02 (0.07) | 0.00 (0.02) | 0.00 (0.00) |
| **Irregular Task** |  |  |  |  |  |  |  |
| Correct (on-time) | 0.35 (0.17) | 0.50 (0.11) | 0.36 (0.17) | 0.42 (0.11) | 0.34 (0.17) | 0.38 (0.12) | 0.66 (0.19) |
| Early | 0.02 (0.04) | 0.00 (0.02) | 0.01 (0.04) | 0.01 (0.04) | 0.02 (0.02) | 0.00 (0.00) | 0.02 (0.05) |
| Late | 0.16 (0.12) | 0.13 (0.11) | 0.20 (0.13) | 0.15 (0.10) | 0.14 (0.12) | 0.15 (0.12) | 0.11 (0.11) |
| Miss | 0.43 (0.18) | 0.35 (0.12) | 0.40 (0.17) | 0.38 (0.16) | 0.48 (0.19) | 0.45 (0.16) | 0.21 (0.13) |
| Incorrect | 0.04 (0.08) | 0.02 (0.06) | 0.03 (0.06) | 0.04 (0.07) | 0.02 (0.04) | 0.02 (0.03) | 0.00 (0.00) |
| **Time-Based Task** |  |  |  |  |  |  |  |
| Correct (on-time) | 0.35 (0.23) | 0.59 (0.19) | 0.37 (0.25) | 0.55 (0.19) | 0.46 (0.20) | 0.51 (0.14) | 0.59 (0.28) |
| Early | 0.05 (0.12) | 0.03 (0.07) | 0.02 (0.05) | 0.02 (0.06) | 0.02 (0.06) | 0.00 (0.00) | 0.07 (0.09) |
| Late | 0.25 (0.20) | 0.17 (0.17) | 0.23 (0.22) | 0.19 (0.16) | 0.17 (0.16) | 0.23 (0.19) | 0.14 (0.14) |
| Miss | 0.35 (0.25) | 0.21 (0.17) | 0.38 (0.33) | 0.24 (0.20) | 0.35 (0.23) | 0.26 (0.18) | 0.20 (0.20) |
| Incorrect | 0.00 (0.00) | 0.00 (0.00) | 0.00 (0.00) | 0.00 (0.00) | 0.00 (0.00) | 0.00 (0.00) | 0.00 (0.00) |

*Note.* Values represent the mean proportions and standard deviations in parentheses.

**Table S11**

## Analysis Comparing Older Adult Training Groups to Untrained Young Adults on Cognitive Outcome Measures

| **Outcome** | **EF Group** | | **WM Group** | | **AC Group** | |
| --- | --- | --- | --- | --- | --- | --- |
|  | **Pre-Test** | **Post-Test** | **Pre-Test** | **Post-Test** | **Pre-Test** | **Post-Test** |
| ***N*-Back Task** | | | | | | |
| 1-Back  (Composite) | *M*_diff_ = -4.77, *SE* =1.24  *p* = .001*  *Odds*_posterior_ = 4.62  (substantial)^d^ | *M*_diff_ = -1.20, *SE* = 1.26  *p* = .898  *Odds*_posterior_ = 0.15  (substantial)^nd^ | *M*_diff_ = -5.54, *SE* = 1.24  *p* < .001*  *Odds*_posterior_ = 23.70  (strong)^d^ | *M*_diff_ = -1.74, *SE* = 1.26  *p* = .999  *Odds*_posterior_ = 0.19  (substantial)^nd^ | *M*_diff_ = -5.05, *SE* = 1.24  *p* = .001*  *Odds*_posterior_ = 8.86  (substantial)^d^ | *M*_diff_ = -4.23, *SE* = 1.26  *p* = .007*  *Odds*_posterior_ = 2.84  (ambiguous) |
| 2-Back  (Composite) | *M*_diff_ = -4.84, *SE* = 1.16  *p* < .001*  *Odds*_posterior_ = 8.36  (substantial)^d^ | *M*_diff_ = -0.94, *SE* = 1.19  *p* = .999  *Odds*_posterior_ = 0.14  (substantial)^nd^ | *M*_diff_ = -5.44, *SE* = 1.16  *p* < .001*  *Odds*_posterior_ = 38.98  (very strong)^d^ | *M*_diff_ = -1.34, *SE* = 1.19  *p* = .999  *Odds*_posterior_ = 0.17  (substantial)^nd^ | *M*_diff_ = -5.26, *SE* = 1.16  *p* < .001*  *Odds*_posterior_ = 28.95  (strong)^d^ | *M*_diff_ = -4.41, *SE* = 1.19  *p* = .002*  *Odds*_posterior_ = 5.24  (substantial)^d^ |
| 3-Back  (Composite) | *M*_diff_ = -6.00, *SE* = 1.08  *p* < .001*  *Odds*_posterior_ = 166.23  (decisive)^d^ | *M*_diff_ = -1.82, *SE* = 1.16  *p* = .719  *Odds*_posterior_ = 0.23  (substantial)^nd^ | *M*_diff_ = -6.86, *SE* = 1.08  *p* < .001*  *Odds*_posterior_ = 1.51×10^3^  (decisive)^d^ | *M*_diff_ = -2.34, *SE* = 1.16  *p* = .273  *Odds*_posterior_ = 0.37  (ambiguous) | *M*_diff_ = -5.75, *SE* = 1.08  *p* < .001*  *Odds*_posterior_ = 137.63  (decisive)^d^ | *M*_diff_ = -5.71, *SE* = 1.16  *p* < .001*  *Odds*_posterior_ = 114.76  (decisive)^d^ |
| **Flanker Task** | | | | | | |
| Flanker Effect  (RT) | *M*_diff_ = 58, *SE* = 13  *p* < .001*  *Odds*_posterior_ = 1.49×10^4^  (decisive)^d^ | *M*_diff_ = 11, *SE* = 11  *p* = .999  *Odds*_posterior_ = 0.70  (ambiguous) | *M*_diff_ = 55, *SE* = 13  *p* < .001*  *Odds*_posterior_ = 1.18×10^4^  (decisive)^d^ | *M*_diff_ = 39, *SE* = 11  *p* = .002*  *Odds*_posterior_ = 655.85  (decisive)^d^ | *M*_diff_ = 57, *SE* = 13  *p* < .001*  *Odds*_posterior_ = 511.35  (decisive)^d^ | *M*_diff_ = 39, *SE* = 11  *p* = .002*  *Odds*_posterior_ = 14.64  (strong)^d^ |
| **Cued Task-Switching Paradigm** | | | | | | |
| Switch Cost  (RT) | *M*_diff_ = 73, *SE* = 16  *p* < .001*  *Odds*_posterior_ = 39.43  (very strong)^d^ | *M*_diff_ = 31, *SE* = 15  *p* = .257  *Odds*_posterior_ = 0.41  (ambiguous) | *M*_diff_ = 65, *SE* = 16  *p* < .001*  *Odds*_posterior_ = 13.34  (strong)^d^ | *M*_diff_ = 58, *SE* = 15  *p* = .002*  *Odds*_posterior_ = 4.74  (substantial)^d^ | *M*_diff_ = 61, *SE* = 16  *p* = .001*  *Odds*_posterior_ = 9.26  (substantial)^d^ | *M*_diff_ = 58, *SE* = 15  *p* = .002*  *Odds*_posterior_ = 6.21  (substantial)^d^ |
| Mixing Cost  (RT) | *M*_diff_ = 210, *SE* = 44  *p* < .001*  *Odds*_posterior_ = 242.26  (decisive)^d^ | *M*_diff_ = 67, *SE* = 43  *p* = .746  *Odds*_posterior_ = 0.31  (substantial)^nd^ | *M*_diff_ = 177, *SE* = 44  *p* < .001*  *Odds*_posterior_ = 52.24  (very strong)^d^ | *M*_diff_ = 155, *SE* = 43  *p* = .003*  *Odds*_posterior_ = 12.34  (strong)^d^ | *M*_diff_ = 176, *SE* = 44  *p* < .001*  *Odds*_posterior_ = 46.00  (very strong)^d^ | *M*_diff_ = 164, *SE* = 43  *p* = .002*  *Odds*_posterior_ = 19.11  (strong)^d^ |
| **WCST** | | | | | | |
| Accuracy  (Proportion Correct) | *M*_diff_ = -0.18, *SE* = 0.04  *p* < .001*  *Odds*_posterior_ = 2.66×10^4^  (decisive)^a^ | *M*_diff_ = -0.04, *SE* = 0.04  *p* = .999  *Odds*_posterior_ = 0.24  (substantial)^nd^ | *M*_diff_ = -0.16, *SE* = 0.04  *p* < .001*  *Odds*_posterior_ = 71.24  (very strong)^d^ | *M*_diff_ = -0.10, *SE* = 0.04  *p* = .085  *Odds*_posterior_ = 2.29  (ambiguous) | *M*_diff_ = -0.20, *SE* = 0.04  *p* < .001*  *Odds*_posterior_ = 1.24×10^3^  (decisive)^d^ | *M*_diff_ = -0.18, *SE* = 0.04  *p* < .001*  *Odds*_posterior_ = 361.83  (decisive)^d^ |
| Number of Perseveration Errors | *M*_diff_ = 7.08, *SE* = 1.60  *p* < .001*  *Odds*_posterior_ = 8.61×10^4^  (decisive)^d^ | *M*_diff_ = 3.03, *SE* = 1.67  *p* = .436  *Odds*_posterior_ = 0.87  (ambiguous) | *M*_diff_ = 5.08, *SE* = 1.60  *p* = .012*  *Odds*_posterior_ = 7.76  (substantial)^d^ | *M*_diff_ = 3.21, *SE* = 1.67  *p* = .344  *Odds*_posterior_ = 0.86  (ambiguous) | *M*_diff_ = 5.94, *SE* = 1.60  *p* = .002*  *Odds*_posterior_ = 38.83  (very strong)^d^ | *M*_diff_ = 4.94, *SE* =1.67  *p* = .023*  *Odds*_posterior_ = 3.67  (substantial)^d^ |
| Number of Categories Completed | *M*_diff_ = -1.27, *SE* = 0.28  *p* < .001*  *Odds*_posterior_ = 8.10×10^3^  (decisive)^d^ | *M*_diff_ = -0.41, *SE* = 0.25  *p* = .670  *Odds*_posterior_ = 0.59  (ambiguous) | *M*_diff_ = -1.23, *SE* = 0.28  *p* < .001*  *Odds*_posterior_ = 370.99  (decisive)^d^ | *M*_diff_ = -0.77, *SE* = 0.25  *p* = .019*  *Odds*_posterior_ = 9.47  (substantial)^d^ | *M*_diff_ = -1.27, *SE* = 0.28  *p* < .001*  *Odds*_posterior_ = 509.51  (decisive)^d^ | *M*_diff_ = -0.96, *SE* = 0.25  *p* = .002*  *Odds*_posterior_ = 20.01  (strong)^d^ |
| **Virtual Week** | | | | | | |
| Regular Tasks  (Proportion Correctly Completed) | *M*_diff_ = -0.40, *SE* = 0.05  *p* < .001*  *Odds*_posterior_ = 4.00×10^7^  (decisive)^d^ | *M*_diff_ = -0.17, *SE* = 0.05  *p* = .003*  *Odds*_posterior_ = 20.80  (strong)^d^ | *M*_diff_ = -0.40, *SE* = 0.05  *p* < .001*  *Odds*_posterior_ = 9.92×10^9^  (decisive)^d^ | *M*_diff_ = -0.22, *SE* = 0.05  *p* < .001*  *Odds*_posterior_ = 190.87  (decisive)^d^ | *M*_diff_ = -0.35, *SE* = 0.05  *p* < .001*  *Odds*_posterior_ = 8.55×10^7^  (decisive)^d^ | *M*_diff_ = -0.33, *SE* = 0.05  *p* < .001*  *Odds*_posterior_ = 1.26×10^7^  (decisive)^d^ |
| Irregular Tasks  (Proportion Correctly Completed) | *M*_diff_ = -0.31, *SE* = 0.05  *p* < .001*  *Odds*_posterior_ = 4.99×10^4^  (decisive)^d^ | *M*_diff_ = -0.16, *SE* = 0.04  *p* < .001*  *Odds*_posterior_ = 15.44  (strong)^d^ | *M*_diff_ = -0.30, *SE* = 0.05  *p* < .001*  *Odds*_posterior_ = 3.13×10^4^  (decisive)^d^ | *M*_diff_ = -0.23, *SE* = 0.04  *p* < .001*  *Odds*_posterior_ = 2.13×10^3^  (decisive)^d^ | *M*_diff_ = -0.32, *SE* = 0.05  *p* < .001*  *Odds*_posterior_ = 9.23×10^4^  (decisive)^d^ | *M*_diff_ = -0.27, *SE* = 0.04  *p* < .001*  *Odds*_posterior_ = 3.70×10^4^  (decisive)^d^ |
| Time-based Tasks  (Proportion Correctly Completed) | *M*_diff_ = -0.25, *SE* = 0.07  *p* = .003*  *Odds*_posterior_ = 10.93  (strong)^d^ | *M*_diff_ = 0.00, *SE* = 0.06  *p* = .999  *Odds*_posterior_ = 0.12  (substantial)^nd^ | *M*_diff_ = -0.22, *SE* = 0.07  *p* = .008*  *Odds*_posterior_ = 4.43  (substantial)^d^ | *M*_diff_ = -0.04, *SE* = 0.06  *p* = .999  *Odds*_posterior_ = 0.14  (substantial)^nd^ | *M*_diff_ = -0.13, *SE* = 0.07  *p* = .039  *Odds*_posterior_ = 1.50  (ambiguous) | *M*_diff_ = -0.08, *SE* = 0.06  *p* = .927  *Odds*_posterior_ = 0.24  (substantial)^nd^ |

*Note.* *M*_diff_ represents older adult score minus younger adult score, **p* < .05 (indicating significant difference in performance compared to young adults), ^d^*Odds*_posterior_ > 3 (indicating at least substantial evidence for difference between groups), ^nd^*Odds*_posterior_ < 0.33 (indicating at least substantial evidence for no difference between groups). *p-*values reflect Bonferroni-adjusted values and posterior *Odds* reflect multiplicity-adjusted values. The posterior *Odds* are the result of multiplying the prior *Odds* by the uncorrected Bayes Factor and reflect the relative plausibility of models after observing data (Goss-Sampson et al., 2020).

**Table S12**

## Descriptive Statistics for Subjective Outcome Measures Across Older Adult Training Groups at Pre-, Post-, and Follow-Up Test

| **Outcomes** | **EF Group** | | | **WM Group** | | | **AC Group** | | |
| --- | --- | --- | --- | --- | --- | --- | --- | --- | --- |
|  | **Pre-Test** | **Post-Test** | **Follow Up** | **Pre-Test** | **Post-Test** | **Follow Up** | **Pre-Test** | **Post-Test** | **Follow Up** |
| **Subjective Ratings of Cognitive Functioning** | | | | | | | | | |
| Attention | 6.50 (1.79) | 7.45 (1.85) | 8.39 (1.57) | 6.64 (1.68) | 7.68 (1.67) | 7.27 (1.64) | 6.68 (1.13) | 6.95 (1.29) | 7.27 (1.67) |
| Everyday Tasks | 8.64 (1.14) | 8.50 (1.50) | 8.74 (1.15) | 8.59 (1.33) | 8.73 (1.24) | 8.50 (1.26) | 8.05 (1.25) | 7.95 (1.29) | 7.86 (1.46) |
| Memory | 6.41 (1.56) | 7.18 (1.50) | 7.64 (1.73) | 6.55 (2.09) | 8.00 (1.80) | 7.41 (2.20) | 5.82 (1.62) | 5.50 (1.26) | 6.09 (2.02) |
| Multitasking | 7.41 (2.54) | 8.09 (1.95) | 7.97 (1.88) | 7.14 (2.27) | 7.41 (2.26) | 6.73 (2.16) | 7.73 (1.78) | 7.64 (1.53) | 7.59 (1.84) |
| Reasoning | 8.23 (1.31) | 8.36 (1.14) | 8.18 (1.10) | 7.82 (1.37) | 8.14 (1.55) | 7.82 (1.50) | 8.00 (1.20) | 7.77 (1.31) | 7.82 (1.47) |
| Speed & RT | 7.64 (1.43) | 8.68 (1.04) | 8.64 (1.09) | 8.18 (1.56) | 8.86 (1.36) | 8.68 (1.32) | 7.09 (1.77) | 6.82 (1.87) | 6.95 (2.03) |
| **PDQ** | | | | | | | | | |
| Attention | 8.41 (3.45) | 6.73 (3.55) | 6.50 (3.78) | 8.95 (3.50) | 7.91 (3.32) | 7.55 (3.66) | 7.05 (3.18) | 7.45 (3.02) | 7.14 (3.00) |
| Retrospective Memory | 8.05 (4.20) | 6.86 (4.45) | 7.27 (4.56) | 10.32 (3.83) | 8.41 (4.06) | 8.55 (3.81) | 8.14 (3.62) | 8.59 (4.11) | 8.36 (3.89) |
| Prospective Memory | 8.00 (3.83) | 7.64 (4.29) | 7.27 (3.69) | 9.50 (4.51) | 7.77 (4.82) | 8.32 (3.80) | 9.64 (3.53) | 9.68 (3.54) | 9.59 (3.87) |
| Planning / Organization | 4.36 (3.62) | 3.68 (2.82) | 3.73 (2.93) | 4.64 (3.09) | 4.00 (3.04) | 3.77 (3.07) | 4.09 (2.62) | 3.82 (2.67) | 3.82 (2.06) |
| **CASP-19** | | | | | | | | | |
| Total | 42.41 (6.43) | 45.09 (5.13) | 44.18 (6.07) | 43.73 (6.18) | 46.91 (5.24) | 44.82 (3.55) | 43.55 (6.03) | 45.64 (5.63) | 45.41 (5.44) |
| **GDS-15** | | | | | | | | | |
| Total | 3.05 (1.46) | 2.73 (1.28) | 2.77 (1.41) | 2.45 (0.86) | 2.32 (0.72) | 2.45 (0.96) | 2.45 (0.80) | 2.45 (0.86) | 2.32 (0.48) |

*Note.* Values represent mean and standard deviation.

## **Normality Checks**

Normality was examined using standardized skew statistics (skew divided by its standard error). We applied a conservative criterion of ±3.29 (p < .001) to identify significant skew. This ensured that only variables with extreme deviations from normality were flagged. Given that repeated-measures ANOVAs are robust to moderate violations of normality with balanced designs, and that Bayesian analyses do not rely on this assumption, we are confident that our results are not meaningfully affected by minor skewness. Below are the standardised skew statistics for key variables at pre-test.

- Age: *std. skew* = 1.34
- Education: *std. skew* = 3.27
- Flanker: *std. skew* = 3.08
- Task Switching (switch cost): *std. skew* = 1.67
- Task Switching (mixing cost): *std. skew* = 0.10
- 1-back: *std. skew* = -3.05
- 2-back: *std. skew* = -0.32
- 3-back: *std. skew* = 0.51
- WCST: *std. skew* = 2.98
- Virtual Week (regular): *std. skew* = 0.64
- Virtual Week (timed): *std. skew* = 0.51
- Virtual Week (irregular): *std. skew* = 0.54
- CASP: *std. skew* = -0.73
- PDQ: *std. skew* = -1.99
- Subjective Memory: *std. skew* = -1.02
- Subjective Attention: *std. skew* = -0.83
- Subjective Reaction Time: *std. skew* = -0.81
- Subjective Reasoning: *std. skew* = -1.47
- Subjective Multitasking: *std. skew* = -2.18
- Subjective Everyday Abilities: *std. skew* = -0.79

## **Additional Analyses**

**Perceptions of Training – Weekly Training Log Data**

Each week participants were asked to record their experience with the training program in a journal. Questions assessed motivation (“I felt motivated to complete my training”), enjoyment (“I found the games enjoyable to play), and level of challenge (“I found the games challenging”). Response options ranged from 1 (*strongly disagree*) to 5 (s*trongly agree*), with higher scores representing higher levels of motivation, enjoyment, and challenge. A summary of descriptives is presented in the table below.

**Descriptive Statistics of Weekly Training Log Data Across Older Adult Training Groups**

|  | **EF** | **WM** | **AC** |
| --- | --- | --- | --- |
| **Motivation** | | | |
| Week 1 | 4.36 (0.90) | 4.45 (0.74) | 4.41 (0.80) |
| Week 2 | 3.68 (0.95) | 3.68 (1.13) | 3.86 (1.17) |
| Week 3 | 3.82 (1.33) | 3.23 (0.87) | 3.82 (1.18) |
| Week 4 | 4.23 (1.07) | 3.27 (1.20) | 3.59 (1.26) |
| **Enjoyment** | | | |
| Week 1 | 3.91 (0.97) | 3.23 (1.23) | 4.09 (0.81) |
| Week 2 | 3.73 (1.12) | 3.32 (0.78) | 4.32 (0.72) |
| Week 3 | 3.23 (1.19) | 3.50 (1.14) | 4.27 (0.83) |
| Week 4 | 3.32 (1.13) | 3.23 (1.23) | 4.27 (0.88) |
| **Challenge** | | | |
| Week 1 | 4.55 (0.51) | 4.64 (0.58) | 2.73 (1.08) |
| Week 2 | 4.59 (0.50) | 4.50 (0.51) | 2.55 (0.86) |
| Week 3 | 4.45 (0.60) | 4.41 (0.50) | 2.59 (0.96) |
| Week 4 | 4.50 (0.51) | 4.50 (0.51) | 2.73 (0.94) |

*Note.* Values represent means and standard deviations.

***Analyses and Results***

A series of 3 Group (EF, WM, AC) × Week (Week 1, Week 2, Week 3, Week 4) ANOVAs were run to examine participants’ self-reported levels of motivation, enjoyment, and challenge throughout the training program.

The analysis for *motivation* revealed decisive evidence for a significant main effect of week, *F*(2.70, 170.09) = 10.94, *p* < .001, η_p_^2^ = .148 (*BF*_incl_ = 10.25×10^3^). Post-hoc analyses indicated that regardless of group, motivation was higher in Week 1 than all other weeks (all *p*s < .001, *Odds*_posterior_ > 163.09). There was strong evidence for no difference in motivation between Weeks 2, 3, and 4 (all *p*s = .999, *Odds*_posterior_ < 0.07). There was ambiguous evidence for no Group × Week interaction, *F*(5.40, 170.09) = 2.06, *p* = .068, η_p_^2^ = .061 (*BF*_incl_ = 0.63), and substantial evidence for no main effect of group, *F*(2, 63) = 1.45, *p* = .242, η_p_^2^ = .044 (*BF*_incl_ = 0.29).

For *enjoyment* the analysis revealed substantial evidence for no Group × Week interaction, *F*(5.22, 164.40) = 1.64, *p* = .150, η_p_^2^ = .049 (*BF*_incl_ = 0.30), and very strong evidence for no main effect of week, *F*(2.61, 164.40) = 0.56, *p* = .647, η_p_^2^ = .009 (*BF*_incl_ = 0.03). There was decisive evidence for a main effect of group, *F*(2, 63) = 10.79, *p* < .001, η_p_^2^ = .255 (*BF*_incl_ = 247.47), with the AC group reporting higher enjoyment throughout the program than the EF (*p* = .004, *Odds*_posterior_ = 1.85×10^3^) and WM groups (*p* < .001, *Odds*_posterior_ = 2.95×10^6^). There was decisive evidence for no difference in enjoyment between the EF and WM groups (*p* = .825, *Odds*_posterior_ = 0.23).

There was very strong evidence for no Group × Week interaction, *F*(6,189) = 0.34, *p* = .914, η_p_^2^ = .011 (*BF*_incl_ = 0.02), and strong evidence for no main effect of week, *F*(3,189) = 0.90, *p* = .442, η_p_^2^ = .014 (*BF*_incl_ = 0.06) for participants’ self-reported *challenge*. There was decisive evidence for a main effect of group, *F*(2, 63) = 92.89, *p* < .001, η_p_^2^ = .747 (*BF*_incl_ = 3.49×10^16^), with the AC group reporting lower levels of challenge than the EF (*p* < .001, *Odds*_posterior_ = 9.01×10^32^) and WM groups (*p* < .001, *Odds*_posterior_ = 4.73×10^32^). There was strong evidence for no difference in level of challenge between the EF and WM groups (*p* = .999, *Odds*_posterior_ = 0.10).

**Flanker Task – Conditions (Congruent vs Incongruent Trials)**

A 3 Group (EF, WM, AC) × 2 Condition (congruent, incongruent) × 2 Time (pre-test, post-test) ANOVA revealed a significant main effect of condition, *F*(1,63) = 24.47, *p* < .001, η_p_^2^ = .280 (*BF*_incl_ = 9.62×10^18^), indicating that the standard Flanker Effect was evident with longer RTs for the incongruent than congruent trials (*M*_diff_ = 85ms, *SE* = 6, *p* < .001; *Odds*_posterior_ = 1.33×10^34^). There was a main effect of time and two-way interactions (Condition × Group, Condition × Time) and a significant 3-way interaction, *F*(2,63) = 3.95, *p* = .024, η_p_^2^ = .111 (*BF*_incl_ = 1.80). Post-hoc analyses revealed that the EF group demonstrated significant improvements in RTs for congruent trials (*M*_diff_ = -35ms, *SE* = 9, *p* < .001; *Odds*_posterior_ = 10.67) and incongruent trials (*M*_diff_ = -81ms, *SE* = 12, *p* < .001; *Odds*_posterior_ = 9.83×10^3^) from pre-test to post-test. Ambiguous evidence for no pre-post RT changes were observed for congruent and incongruent trials for the WM (*p*s > .107, *Odds*_posterior_ < 1.44) and AC groups (*p*s > .068, *Odds*_posterior_ < 0.67).

**Task-Switching Paradigm – Switch Cost Conditions (Nonswitch vs Switch Trials)**

The 3 Group (EF, WM, AC) × 2 Trial Type (nonswitch trial, switch trial) × 2 Time (pre-test, post-test) revealed a main effect of trial type, *F*(1,63) = 995.10, *p* < .001, η_p_^2^ = .940 (*BF*_incl_ = 7.55×10^36^), indicating longer RTs for switch than nonswitch trials (*M*_diff_ = 142ms, *SE* = 5, *p* < .001; *Odds*_posterior_ = 1.31×10^28^). There was a significant main effect of time, significant two-way interactions (Time × Group, Time × Trial Type) and the three-way interaction was significant, *F*(2,63) = 19.94, *p* < .001, η_p_^2^ = .240 (*BF*_incl_ = 2.45×10^5^). Post-hoc analyses revealed that the EF group demonstrated significant improvements in RTs for switch trials (*M*_diff_ = -219ms, *SE* = 19, *p* < .001; *Odds*_posterior_ = 1.13×10^4^) and nonswitch trials (*M*_diff_ = -177ms, *SE* = 20, *p* < .001; *Odds*_posterior_ = 421.51) from pre-test to post-test. The EF group exhibited a larger pre-post decrease in reaction time for switch trials than nonswitch trials (*M*_diff_ = -42ms, *SE* = 7, *p* < .001; *Odds*_posterior_ = 52.96). In contrast, no pre-post RT changes were observed for either switch or nonswitch trials for the WM (*p*s > .236, *Odds*_posterior_ < 0.69) or AC groups (*p*s > .348, *Odds*_posterior_ < 0.41).

**Task-Switching Paradigm – Mixing Cost Conditions (Mixed Task Trials vs Single Task Trials)**

The 3 Group (EF, WM, AC) × 2 Trial Type (mixed task trial, single task trial) × 2 Time (pre-test, post-test) revealed a main effect of trial type, *F*(1,63) = 393.10, *p* < .001, η_p_^2^ = .862 (*BF*_incl_ = 6.06×10^26^), with longer RTs for mixed task than single task trials (*M*_diff_ = 359ms, *SE* = 18, *p* < .001; *Odds*_posterior_ = 3.16×10^50^). There was a significant main effect of time, significant two-way interactions (Time × Group, Time × Trial Type) and the three-way interaction was significant, *F*(2,63) = 14.54, *p* < .001, η_p_^2^ = .316 (*BF*_incl_ = 1.23×10^4^). Post-hoc analyses revealed that the EF group demonstrated significant improvements in RTs for single task trials (*M*_diff_ = -55ms, *SE* = 13, *p* < .001; *Odds*_posterior_ = 12.64) and mixed task trials (*M*_diff_ = -198ms, *SE* = 20, *p* < .001; *Odds*_posterior_ = 2.36×10^4^) from pre-test to post-test. The EF group exhibited a larger pre-post decrease in reaction time in mixed task trials than single task trials (*M*_diff_ = -143ms, *SE* = 19, *p* < .001; *Odds*_posterior_ = 866.27). In contrast, no pre-post RT changes were observed for either single task or mixed task trials for the WM (*p*s > .325, *Odds*_posterior_ < 0.48) or AC groups (*p*s > .393, *Odds*_posterior_ < 0.59).

**Table S13**

## Statistical Analysis Results for Interactions and Main Effects for Cognitive Outcomes Among Older Adult Training Groups (Pre- to Post-Test)

| **Outcomes** | **Interactions** | **Main Effects** |
| --- | --- | --- |
|  |  |  |
| **Cognitive Outcomes** | | |
| ***N*-Back Task** |  |  |
| *N*-Back  (Composite) | Time×Condition×Group: *F*(3.52,110.95) = 0.76, *p* = .554, η_p_^2^ _=_ .024  (*BF*_incl_ = 0.07) – strong^nd^  Time×Group: *F*(2,63) = 15.90, *p* < .001, η_p_^2^ _=_ .335*  (7.08×10^3^) – decisive^d^  Time×Condition: *F*(1.76,110.95) = 0.17, *p* = .846, η_p_^2^ _=_ .003  (*BF*_incl_ = 0.06) – strong^nd^  Condition×Group: *F*(3.39,106.75) = 0.14, *p* =.967, η_p_^2^ _=_ .004  (*BF*_incl_ = 0.03) – very strong^nd^ | Condition: *F*(1.69,106.75) = 126.64, *p* < .001, η_p_^2^ _=_ .668*  (*BF*_incl_ = 3.12×10^28^) – decisive^d^  Time: *F*(1,63) = 98.70, *p* < .001, η_p_^2^ _=_ .610*  (*BF*_incl_ = 8.20×10^8^) – decisive^d^  Group: *F*(2,63) = 5.18, *p* = .008, η_p_^2^ _=_ .141  (*BF*_incl_ = 5.60) – substantial^d^ |
| **Flanker Task** |  |  |
| Flanker Effect  (RT, ms) | Time×Group: *F*(2,63) = 3.95, *p* = .024, η_p_^2^ _=_ .111*  (*BF*_incl_ = 2.32) – ambiguous | Time: *F*(1,63) = 27.93, *p* < .001, η_p_^2^ _=_ . 307*  (*BF*_incl_ = 3.91×10^3^) – decisive^d^  Group: *F*(2,63) = 0.52, *p* = .596, η_p_^2^ _=_ .016  (*BF*_incl_ = 0.31) – substantial^nd^ |
| **Cued Task-Switching Paradigm** |  |  |
| Switch Cost  (RT, ms) | Time×Group: *F*(2,63) = 9.73, *p* < .001, η_p_^2^ _=_ .236*  (*BF*_incl_ = 128.13) – decisive^d^ | Time: *F*(1,63) = 19.94, *p <* .001, η_p_^2^ _=_ .240*  (*BF*_incl_ = 128.94) – decisive^d^  Group: *F*(2,63) = 0.39, *p* = .682, η_p_^2^ _=_ .012  (*BF*_incl_ = 0.24) – substantial^nd^ |
| Mixing Cost  (RT, ms) | Time×Group: *F*(2,63) = 14.54, *p* < .001, η_p_^2^ _=_ .316*  (*BF*_incl_ = 2.55×10^3^) – decisive^d^ | Time: *F*(1,63) = 28.63, *p* < .001, η_p_^2^ _=_ .312*  (*BF*_incl_ = 616.01) – decisive^d^  Group: *F*(2,63) = 0.30, *p* = .740, η_p_^2^ _=_ .010  (*BF*_incl_ = 0.34) – ambiguous |
| **WCST** |  |  |
| Accuracy  (Proportion Correct) | Time×Group: *F*(2,63) = 3.75, *p* = .029, η_p_^2^ _=_ .106*  (*BF*_incl_ = 1.95) – ambiguous | Time: *F*(1,63) = 19.51, *p* < .001, η_p_^2^ _=_ .236*  (*BF*_incl_ = 278.98) – decisive^d^  Group: *F*(2,63) = 1.77, *p* = .179, η_p_^2^ _=_ .053  (*BF*_incl_ = 0.65) – ambiguous |
| Number of Perseveration Errors | Time×Group: *F*(2,63) = 1.14, *p* = .326, η_p_^2^ _=_ .035  (*BF*_incl_ = 0.37) – ambiguous | Time: *F*(1,63) = 7.36, *p* = .009, η_p_^2^ _=_ .105*  (*BF*_incl_ = 4.51) – substantial^d^  Group: *F*(2,63) = 0.30, *p* = .739, η_p_^2^ _=_ .010  (*BF*_incl_ = 0.19) – substantial^nd^ |
| Number of Categories | Time×Group: *F*(2,63) = 1.97, *p* = .149, η_p_^2^ _=_ .059  (*BF*_incl_ = 0.49) – ambiguous | Time: *F*(1,63) = 21.77, *p* < .001, η_p_^2^ _=_ .257*  (*BF*_incl_ = 847.64) – decisive^d^  Group: *F*(2,63) = 0.43, *p* = .653, η_p_^2^ _=_ .013  (*BF*_incl_ = 0.28) – substantial^nd^ |
| **Virtual Week** |  |  |
| Regular Tasks  (Proportion Correctly Completed) | Time×Group: *F*(2,63) = 9.25, *p* < .001, η_p_^2^ _=_ .227*  (*BF*_incl_ = 129.48) – decisive^d^ | Time: *F*(1,63) = 46.99, *p* < .001, η_p_^2^ _=_ .427*  (*BF*_incl_ = 2.40×10^5^) – decisive^d^  Group: *F*(2,63) = 0.60, *p* = .555, η_p_^2^ _=_ .019  (*BF*_incl_ = 0.24) – substantial^nd^ |
| Irregular Tasks  (Proportion Correctly Completed) | Time×Group: *F*(2,63) = 5.10, *p* = .009, η_p_^2^ _=_ .139*  (*BF*_incl_ = 6.68) – substantial^d^ | Time: *F*(1,63) = 34.00, *p* < .001, η_p_^2^ _=_ .350*  (*BF*_incl_ = 1.84×10^4^) – decisive^d^  Group: *F*(2,63) = 1.32, *p* = .274, η_p_^2^ _=_ .040  (*BF*_incl_ = 0.48) – ambiguous |
| Time-Based Tasks  (Proportion Correctly Completed) | Time×Group: *F*(2,63) = 5.54, *p* = .006, η_p_^2^ _=_ .150*  (*BF*_incl_ = 7.58) – substantial^d^ | Time: *F*(1,63) = 39.59, *p* < .001, η_p_^2^ _=_ .386*  (*BF*_incl_ = 1.04×10^5^) – decisive^d^  Group: *F*(2,63) = 0.13, *p* = .877, η_p_^2^ _=_ .004  (*BF*_incl_ = 0.17) – substantial^nd^ |
| **CFIT** |  |  |
| Accuracy  (Proportion Correct) | Time×Group: *F*(2,63) = 0.69, *p* = .506, η_p_^2^ _=_ .021  (*BF*_incl_ = 0.22) – substantial^nd^ | Time: *F*(1,63) = 7.63, *p* = .007, η_p_^2^ _=_ .108*  (*BF*_incl_ = 6.93) – substantial^d^  Group: *F*(2,63) = 1.47, *p* = .238, η_p_^2^ _=_ .045  (*BF*_incl_ = 0.32) – substantial^nd^ |
| Completion  (Proportion Completed) | Time×Group: *F*(2,63) = 0.11, *p* = .895, η_p_^2^ _=_ .004  (*BF*_incl_ = 0.16) – substantial^nd^ | Time: *F*(1,63) = 0.03, *p* = .872, η_p_^2^ _=_ .000  (*BF*_incl_ = 0.18) – substantial^nd^  Group: *F*(2,63) = 0.19, *p* = .826, η_p_^2^ _=_ .006  (*BF*_incl_ = 0.14) – substantial^nd^ |

*Note.* Null hypothesis significance testing statistics (*F*-test, p-value) and Bayes Factors (*BF*_incl_) presented for each interaction and main effect. **p* < .05 (indicating significant interaction or main effect). ^d^*BF*_incl_ > 3 (indicating at least substantial evidence for interaction or main effect), ^nd^*BF*_incl_ < 0.33 (indicating at least substantial evidence for no interaction or main effect).

**Table S14**

## Statistical Analysis Results for Interactions and Main Effects for Subjective Outcomes Among Older Adult Training Groups (Pre-, Post-, and Follow-up Test)

| **Outcomes** | **Interactions** | **Main Effects** |
| --- | --- | --- |
|  |  |  |
| **Subjective Measures of Cognition and Well-being** | | |
| **Subjective Ratings of Cognitive Functioning** | | |
| Attention | Time×Group: *F*(4,126) = 3.58, *p* = .008, η_p_^2^ _=_ .102*  (*BF*_incl_ = 5.41) – substantial^d^ | Time: *F*(2,126) = 15.78, *p* < .001, η_p_^2^ _=_ .200*  (*BF*_incl_ = 9.95×10^3^) – decisive^d^  Group: *F*(2,63) = 0.72, *p* = .491, η_p_^2^ _=_ .022  (*BF*_incl_ = 0.26) – substantial^nd^ |
| Everyday Tasks | Time×Group: *F*(3.60,113.31) = 0.92, *p* = .450, η_p_^2^ _=_ .028  (*BF*_incl_ = 0.11) – substantial^nd^ | Time: *F*(1.80,113.31) = 0.12, = .864, η_p_^2^ _=_ .005  (*BF*_incl_ = 0.06) – strong^nd^  Group: *F*(2,63) = 2.28, *p* = .111, η_p_^2^ _=_ .067  (*BF*_incl_ = 1.02) – ambiguous |
| Memory | Time×Group: *F*(4,126) = 3.71, *p* = .007, η_p_^2^ _=_ .105*  (*BF*_incl_ = 5.41) – substantial^d^ | Time: *F*(2,126) = 8.05, *p* < .001, η_p_^2^ _=_ .113*  (*BF*_incl_ = 27.47) – strong^d^  Group: *F*(2,63) = 6.62, *p* = .002, η_p_^2^ _=_ .174*  (*BF*_incl_ = 16.35) – strong^d^ |
| Multitasking | Time×Group: *F*(3.38, 106.40) = 2.60, *p* = .111, η_p_^2^ _=_ .026  (*BF*_incl_ = 1.03) – ambiguous | Time: *F*(1.69,106.40) = 2.41, *p* = .103, η_p_^2^ _=_ .037  (*BF*_incl_ = 0.38) – ambiguous  Group: *F*(2,63) = 0.88, *p* = .421, η_p_^2^ _=_ .027  (*BF*_incl_ = 0.49) – ambiguous |
| Reasoning | Time×Group: *F*(3.42,107.63) = 0.69, *p* = .578, η_p_^2^ _=_ .021  (*BF*_incl_ = 0.09) – strong^nd^ | Time: *F*(1.71,107.63) = 0.60, *p* = .524, η_p_^2^ _=_ .009  (*BF*_incl_ = 0.09) – strong^nd^  Group: *F*(2,63) = 0.73, *p* = .488, η_p_^2^ _=_ .023  (*BF*_incl_ = 0.32) – substantial^nd^ |
| Speed & RT | Time×Group: *F*(4,126) = 4.28, *p* = .003, η_p_^2^ _=_ .120*  (*BF*_incl_ = 11.77) – strong^d^ | Time: *F*(2,126) = 6.99, *p* = .001, η_p_^2^ _=_ .100*  (*BF*_incl_ = 10.76) – strong^d^  Group: *F*(2,63) = 8.88, *p* < .001, η_p_^2^ _=_ .220*  (*BF*_incl_ = 70.46) – very strong^d^ |
| **PDQ** | | |
| Attention | Time×Group: *F*(4,126) = 5.56, *p* < .001, η_p_^2^ _=_ .150*  (*BF*_incl_ = 43.20) – very strong^d^ | Time: *F*(2,126) = 13.76, *p* < .001, η_p_^2^ _=_ .179*  (*BF*_incl_ = 438.65) – decisive^d^  Group: *F*(2,63) = 0.60, *p* = .555, η_p_^2^ _=_ .019  (*BF*_incl_ = 0.41) – ambiguous |
| Retrospective Memory | Time×Group: *F*(3.64,114.58) = 8.28, *p* < .001, η_p_^2^ _=_ .208*  (*BF*_incl_ = 557.57) – decisive^d^ | Time: *F*(1.82,114.58) = 13.35, *p* < .001, η_p_^2^ _=_ .175*  (*BF*_incl_ = 51.08) – very strong^d^  Group: *F*(2,63) = 1.01, *p* = .370, η_p_^2^ _=_ .031  (*BF*_incl_ = 0.56) – ambiguous |
| Prospective Memory | Time×Group: *F*(3.14,98.79) = 4.15, *p* = .007, η_p_^2^ _=_ .116*  (*BF*_incl_ = 13.10) – strong^d^ | Time: *F*(1.57,98.79) = 7.89, *p* = .002, η_p_^2^ _=_ .111*  (*BF*_incl_ = 3.75) – substantial^d^  Group: *F*(2,63) = 1.45, *p* = .243, η_p_^2^ _=_ .044  (*BF*_incl_ = 0.72) – ambiguous |
| Planning / Organization | Time×Group: *F*(3.55,111.93) = 0.40, *p* = .784, η_p_^2^ _=_ .013  (*BF*_incl_ = 0.06) – strong^nd^ | Time: *F*(1.78,111.93) = 4.78, *p* = .013, η_p_^2^ _=_ .071*  (*BF*_incl_ = 3.19) – substantial^d^  Group: *F*(2,63) = 0.05, *p* = .954, η_p_^2^ _=_ .002  (*BF*_incl_ = 0.32) – substantial^nd^ |
| **CASP-19** | | |
| Total | Time×Group: *F*(3.53,111.14) = 0.59, *p* = .652, η_p_^2^ _=_ .018  (*BF*_incl_ = 0.07) – strong^nd^ | Time: *F*(1.76,111.14) = 13.75, *p* < .001, η_p_^2^ _=_ .179*  (*BF*_incl_ = 3.42×10^3^) – decisive^d^  Group: *F*(2,63) = 0.38, *p* = .689, η_p_^2^ _=_ .012  (*BF*_incl_ = 0.31) – substantial^nd^ |
| **GDS-15** | | |
| Total | Time×Group: *F*(3.45,108.75) = 0.93, *p* = .439, η_p_^2^ _=_ .029  (*BF*_incl_ = 0.12) – substantial^nd^ | Time: *F*(1.73,108.75) = 1.84, *p* = .169, η_p_^2^ _=_ .028  (*BF*_incl_ = 0.26) – substantial^nd^  Group: *F*(2,63) = 1.58, *p* = .213, η_p_^2^ _=_ .048  (*BF*_incl_ = 0.64) – ambiguous |

*Note.* Null hypothesis significance testing statistics (*F*-test, p-value) and Bayes Factors (*BF*_incl_) presented for each interaction and main effect. **p* < .05 (indicating significant interaction or main effect). ^d^*BF*_incl_ > 3 (indicating at least substantial evidence for interaction or main effect), ^nd^*BF*_incl_ < 0.33 (indicating at least substantial evidence for no interaction or main effect).
